# Supercomputing Multi-Ligand Modeling, Simulation, Wavelet Analysis and Surface Plasmon Resonance to Develop Novel Combination Drugs: A Case Study of Arbidol and Baicalein Against Main Protease of SARS-CoV-2

**DOI:** 10.3390/ph18071054

**Published:** 2025-07-17

**Authors:** Hong Li, Hailong Su, Akari Komori, Shuxuan Yang, Hailang Luo, Angela Wei Hong Yang, Xiaomin Sun, Hongwei Li, Andrew Hung, Xiaoshan Zhao

**Affiliations:** 1 Department of Traditional Chinese Medicine, Zhujiang Hospital, Southern Medical University, Guangzhou 510280, China; gdsyiceman@smu.edu.cn; 2 School of Traditional Chinese Medicine, Southern Medical University, Guangzhou 510515, China; ywwy@smu.edu.cn (S.Y.); min1980@smu.edu.cn (X.S.); 3 School of Science, STEM College, RMIT University, Melbourne, VIC 3000, Australia; s3172209@student.rmit.edu.au; 4 School of Laboratory Medicine and Biotechnology, Southern Medical University, Guangzhou 510515, China; yzdxshl@126.com; 5 School of Health and Biomedical Sciences, STEM College, RMIT University, Bundoora, VIC 3083, Australia; angela.yang@rmit.edu.au

**Keywords:** synergism, structure-based multi-ligand molecular modeling, umifenovir, Toujie Quwen Granules, COVID-19

## Abstract

**Background/Objectives**: Combination therapies using traditional Chinese medicine and Western drugs have gained attention for their enhanced therapeutic effects and reduced side effects. Toujie Quwen Granules (TQG), known for its antiviral properties, particularly against respiratory viruses, could offer new treatment strategies when combined with antiviral drugs like arbidol, especially for diseases such as Coronavirus disease. This study investigates the synergistic mechanisms between arbidol and components from TQG against the severe acute respiratory syndrome coronavirus 2 (SARS-CoV-2) main protease (M^pro^). **Methods**: We identified compounds from TQG via existing data. Multi-ligand molecular docking, pharmacokinetic/toxicity screening, and preliminary simulations were performed to assess potential synergistic compounds with arbidol. UPLC-Q-Exactive Orbitrap-MS verified the presence of these compounds. Extended simulations and in vitro assays, including Luciferase and surface plasmon resonance, validated the findings. **Results**: Five compounds interacted with arbidol in synergy based on docking and preliminary dynamics simulation results. Only Baicalein (HQA004) could be identified in the herbal remedy by untargeted metabolomics, with ideal pharmacokinetic properties, and as a non-toxic compound. Extended simulations revealed that HQA004 enhanced arbidol’s antiviral activity via a *“Far” Addition Mechanism #2*, with an optimal 2:1 arbidol:HQA004 ratio. The movements of arbidol (diffusion and intramolecular conformational shifts) in the system were significantly reduced by HQA004, which may be the main reason for the synergism that occurred. In vitro experiments confirmed an increased inhibition of M^pro^ by the combination. **Conclusions**: HQA004 demonstrated synergistic potential with arbidol in inhibiting M^pro^. The development of combination therapies integrating Western and herbal medicine is supported by these findings for effective antiviral treatments.

## 1. Introduction

A combination drug, commonly used to treat various diseases over an extended period, refers to a medication containing multiple active ingredients blended into a single dosage form [1]. The main rationale behind the utilization of combination drugs is their potential to enhance therapeutic outcomes by leveraging the favorable interaction and synergistic effects of the ingredients [2]. Developing combination drugs serves additional objectives, such as mitigating the toxicity of the ingredients to minimize the occurrence of adverse effects [3], as well as addressing the drug resistance challenge in the management of microbial infections [4]. In clinical practice, several combination drugs have been widely administered to patients [5,6,7]. Nevertheless, the task of identifying chemical components that exhibit synergistic mechanisms and possess the potential to be combined into a single medication remains a formidable challenge [8].

The integration of traditional Chinese and Western medicine combines two different medical theories and the efficacy of traditional Chinese medicine (TCM) and Western medicine to highlight the benefits of both medical systems effectively [9]. In China, it is common to integrate TCM with Western medications for the purpose of increasing effectiveness or relieving side effects [10]. The reason to integrate is to enhance the effectiveness of Western medicine by leveraging the therapeutic properties of traditional herbs, or to minimize the adverse effects of Western medications by utilizing the detoxifying and regulating effects of traditional herbs [11]. Furthermore, there is a trend to develop novel drugs with unique healing effects via combining TCM and Western medicines, which incorporates the drug development techniques from both medical systems [12]. These drugs can be tailored according to the TCM principle of syndrome differentiation and treatment, therefore, providing a more precise and personalized treatment plan [13,14].

In recent years, there have been attempts to explore the cooperativity of two ligands targeting the same protein to enhance the overall therapeutic effects, and all of these studies used the structure-based multi-ligand molecular modeling (SMMM) approach [8,15,16]. The SMMM approach, including multi-ligand molecular docking and multi-ligand molecular dynamics (MD) simulation, can predict interactions (synergy or competition) between two monomers for a particular protein [17]. However, this technique is seldom applied in the investigation of the synergistic effects between herbal agents and Western medications. Furthermore, the application of a supercomputer could not only greatly reduce the calculation time (4.5 × 10^5^ times), but also improve the docking accuracy and precision by at least 6 times greater than that of ordinary computers [18]. Our previously published paper also applied wavelet analysis to seek synergistic effects between two small molecules [8]. Wavelet analysis facilitated the identification of cyclic patterns in the dynamics of residue interactions, enhancing the understanding of compound synergy [19]. It is important to note that there is a trend to develop novel combination drugs by adding a small molecule to an approved drug, for example, nirmatrelvir/ritonavir (Paxlovid) for COVID-19 [7]. However, developing a novel combination drug presents challenges due to the complexity of understanding the synergistic effects of multiple compounds in the human body. Consequently, identifying suitable candidates for such a combination drug is time-consuming and labor-intensive. Here, we used an example of arbidol (ARB) and baicalein against COVID-19 to propose a new method for developing novel combination drugs. This method is based on the theory of integration of traditional Chinese and Western medicine, as well as our comprehensive approach, including supercomputing multi-ligand modeling, simulation, and wavelet analysis, and then validated by enzyme assay and surface plasmon resonance.

The severe acute respiratory syndrome coronavirus 2 (SARS-CoV-2) is the main reason leading to COVID-19, which is an infectious disease [20], leading to more than 778,000,000 cases reported and around 7,100,000 deaths [21]. Clinical observations showed that symptoms, for example, fever, sore throat, diarrhea, and shortness of breath, are common to see [22]. Flu-like symptoms, such as rhinorrhea, nasal congestion, headache, and sneeze, or asymptomatic infection, were reported in some people [22]. For the prognosis of COVID-19, patients, especially older patients or patients who have underlying chronic health conditions, such as high blood pressure or diabetes, may be at a higher risk of developing a severe illness than others [23]. For the management of COVID-19, some investigational medicines, which are currently tested in silico, in vitro, in vivo, and in clinical studies, have been used in clinical practice, such as ARB, Paxlovid, remdesivir, ribavirin, and lopinavir–ritonavir [24,25]. Among all the investigational drugs, ARB, also called umifenovir, is a broad-spectrum antiviral drug that was initially made by scientists from Russia for certain enveloped and non-enveloped viruses, such as influenza A, influenza B, and hepatitis C viruses [26,27]. Since February 2020, ARB has been recommended as one of the routine antiviral therapies for COVID-19 by the Chinese health authority [28], as it leads to a better therapeutic outcome. For example, it significantly reduced the period and severity of fever in the first five days, compared to the one without ARB or other anti-viral drugs (oseltamivir and ribavirin) [29]. It also distinctly decreased the duration of hospitalization, compared to lopinavir/ritonavir [30]. It cleared 75.7% of the virus in patients’ respiratory tissues 14 days after oral intake, compared to oseltamivir (clearance rate: 61.5%) [31]. However, the therapeutic effects of ARB are still limited [32]. Therefore, if the clinical efficacy of ARB for COVID-19 could be improved, it would be greatly beneficial for hundreds of millions of patients.

Different from the traditional method of screening compounds from small-molecule databases, this study used the 16 herbs from Toujie Quwen Granules (TQG) (Table S1) [33] as the source of a small molecular library to identify herbal compounds with potential synergistic effects with ARB against the main protease (M^pro^) of SARS-CoV-2. It is significant to know that M^pro^ plays an essential role during virus replication. This protease is a dimeric protein with two subunits. Each subunit has three domains, and the active site lies between domains I and II. Additionally, in the active binding pockets, key residues (Cys145 and His41) assist the enzyme in cutting viral polyproteins into functional parts [34]. During replication, M^pro^ processes pp1a and pp1ab into non-structural proteins, including RdRp for RNA synthesis. Without M^pro^, the virus cannot make new copies [34]. TQG is a novel Chinese patent medicine specifically developed for COVID-19 based on the Chinese medicine treatment experience of severe acute respiratory syndrome, with evidence to support its treatment effects [33,35]. A recently published randomized controlled trial reported that the total effective rate of the TQG plus ARB group was 89.2% which is higher than the ARB-only group (69.44%), with statistical significance (*p* < 0.05) [36]. Specifically, the combination group presented better symptom scores, expression of T cell counts, as well as immune function recovery, compared to ARB monotherapy [36]. Our recently published work also reported that compounds from TQG may inhibit the biological activities of M^pro^ of SARS-CoV-2 [8]. It is reasonable to assume that compounds present in TQG may enhance the therapeutic effects of ARB in the treatment of COVID-19. Consequently, this study aimed to screen compounds from TQG that may enhance ARB’s ability to target the M^pro^ of SARS-CoV-2, which is a known drug target and has been proven as a highly efficient target against COVID-19 in our previous publications. This study also elucidated the possible mechanisms of action of TQG combined with ARB on COVID-19. Our investigation, moreover, determined whether our strategy to identify novel combination drugs based on the theory of integration of TCM and Western medicine through the SMMM approach is a suitable and creative strategy.

## 2. Results

### 2.1. Five Herbal Compounds Identified from Toujie Quwen Granule Through Multi-Ligand Docking May Have Synergistic Effects with Arbidol

**Figure 1:**
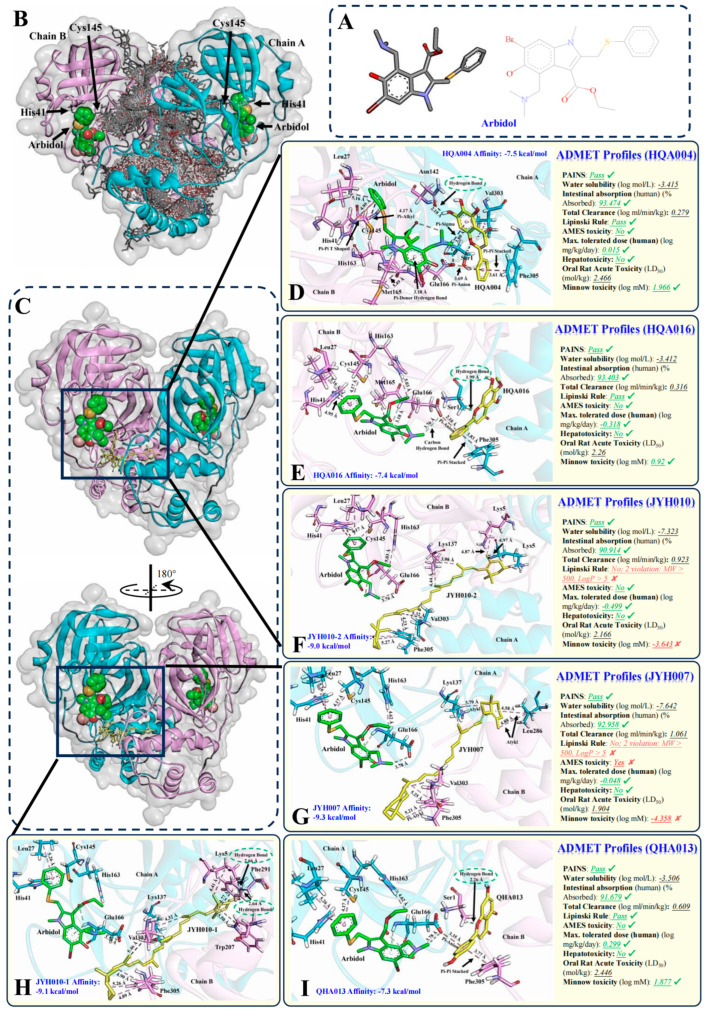
Synergistic binding modes of the five compounds in six conformations directly connected to arbidol against the main protease of SARS-CoV-2 and their pharmacokinetic and toxicity properties. (**A**) 3D/2D structures of arbidol. (**B**) Overview of the docking results of all compounds from Toujie Quwen Granules against the complex of arbidol-M^pro^. (**C**) Overview of the binding positions of the five compounds in six conformations. (**D**–**I**) 3D binding poses of specific compounds with their detailed pharmacokinetic and toxicity properties. Hydrogen bonds are represented by green dashed lines, while hydrophobic bonds are in pink. Corresponding compound names are provided in Table S2.

We retrieved the compound structures of all 238 TQG compounds from our previously published study (Tables S2 and S3) and then processed them for multi-ligand molecular docking to identify compounds with potential synergistic effects with the initial inhibitor, ARB (Figure 1A). We predicted 238 docking results with an average binding affinity of −7.85 kcal/mol (ranging from −4.8 to −10.3 kcal/mol) (Tables S4 and S5). Among all docking results, high binding affinity compounds (<−7 kcal/mol) [37] accounted for more than 90% (215 out of 238 compounds). The top 10 compounds included CBM011 (−10.3 kcal/mol), CBM012 (−10.1 kcal/mol), JYH011 (−10.1 kcal/mol), CBM010 (−10 kcal/mol), TZS003 (−10 kcal/mol), CBM004 (−9.9 kcal/mol), ZBM003 (−9.9 kcal/mol), JYH007 (−9.8 kcal/mol), CBM005 (−9.7 kcal/mol), and DQY003 (−9.7 kcal/mol). In our previous publication, we concluded that compounds located at the same binding pocket as the initial inhibitor, as well as physically close to it, may have synergistic effects with the inhibitor [8]. However, as we observed the docking poses, there are no compounds directly close to ARB, neither the top 10 compounds nor other compounds. Considering that clinical evidence has proved the synergistic mechanisms existing between the granule and ARB, it is reasonable to assume that synergistic ligands exist in the herbal mixture. Having nine confirmations being predicted for each of the dockings by PyRx, we also considered the other eight confirmations, rather than the conventional procedure of considering only the top 1 confirmation. Consequently, a total of 2142 binding poses were considered for physical observation (Figure 1B). Based on our observation, five herbal compounds in six conformations showed potential synergistic effects with ARB, including HQA004 (conformation 6; −7.5 kcal/mol), HQA016 (conformation 5; −7.4 kcal/mol), JYH010-2 (conformation 2; −9 kcal/mol), JYH007 (conformation 5; −9.3 kcal/mol), JYH010-1 (conformation 1; −9.1 kcal/mol) and QHA013 (conformation 4; 7.3 kcal/mol) (Figure 1C–I). Except for JYH007 and JYH010-2, H-bonds with a short distance of less than 3 Å were detected between herbal compounds and M^pro^.

Several phenomena were observed after further analysis of the binding conformations. Firstly, HQA004 was the only ligand that directly connected to ARB at the active binding pocket via a hydrophobic interaction (Pi-sigma) with a distance of 3.8 Å. According to our previously published work, when a ligand is directly connected to the initial ligand, they are more likely to exhibit synergistic behavior against the protein. Based on the binding poses, a hydrogen bond (H-bond) was also noted between HQA004 and Asn142 (2.18 Å). Additionally, large numbers of hydrophobic interactions were found with short distances to neighboring residues within the complex of HQA004-M^pro^-ARB. These residues included Ser1, leu27, His41, Cys145, Met165, Glu166, Val303 and Phe305. The involvement of diverse binding interactions throughout all the domains of the protease in this compactly structured complex may contribute to the stabilization of M^pro^.

Secondly, Glu166 was found to act as a “sharing” residue between ARB and the three herbal compounds, including HQA004, HQA016, and QHA013. The distances from Glu166 to ARB and the herbal compounds were within 4 Å with various interaction modes that typically involved hydrophobic bonds and carbon-hydrogen bonds. This sharing residue may provide forces from two different directions to maintain the proposed positions of both ARB and herbal ligands. This unique structure may be the source of the synergistic effects and better stability of the protein than when either of the ligands was inserted into the protein. Thirdly, all three conformations from two long-chain herbal ligands, JYH007 and JYH010, showed a high binding affinity (≥−9 kcal/mol). However, neither of them directly connected to ARB nor interacted with a shared residue of the protease with ARB. The existence of synergistic effects is unclear from these ligands, and further studies are required to verify the effects.

### 2.2. HQA004, HQA016 and QHA013 Passed the Pharmacokinetic and Toxicity Screening

To ensure the candidate compounds fulfilled the characteristics of medicinal chemistry, we used two online platforms to predict the absorption, distribution, metabolism, excretion, and toxicity (ADME/T) properties of the aforementioned five compounds (Figure 1D–I). Firstly, we used the online Pan-Assay Interference Compounds (PAINS) platform to check whether false positives in biological examinations may happen in some chemical components [38]. All five compounds passed the PAINS filter, meaning a high probability of false positives would not occur. Then, we used pkCSM, a machine-learning platform, to evaluate other pharmacokinetics and toxicity properties. Specifically, three of the compounds, HQA004, HQA016, and QHA013, were aqueously soluble (Log solubility in mol/L between −4 and −2), whereas the other two compounds, JYH007 and JYH010, were poorly soluble (Log solubility in mol/L between −10 and −6). However, all the compounds received a good intestinal absorption rate (>90%), which means that the water solubility of the five compounds, especially the two poorly soluble ligands, may not affect their biological absorption rate in the human body. In terms of the total clearance, our findings demonstrate elevated total clearance values (Log total clearance) for HQA004 (0.279), HQA016 (0.316), QHA013 (0.609), JYH007 (1.061), and JYH010 (0.923), signifying that these drugs have the potential to be eliminated from the tissues. When the total clearance value is higher, it indicates that a greater number of drugs are effectively removed from both the tissues and the bloodstream [39]. This capability is particularly valuable if potentially toxic molecules are generated during transportation and metabolism, preventing their accumulation in tissues and the subsequent occurrence of cytotoxic effects [40]. For drug-likeness evaluation, the Lipinski rule was applied. Among the five compounds, HQA004, HQA016, and QHA013 passed all the rules. Nevertheless, JYH007 and JYH010 violated two of the rules, including molecular weight of less than 500 Da and lipophilicity (LogP values) of less than 5, which means these two compounds may not fulfill the criteria for being suitable as drugs.

The toxicity properties of a ligand are important to assess, since they can enhance the success rate of developing a safe drug; it is significant to understand the toxicity properties of the ligand [41]. Thus, we employed five parameters on the pkCSM server. Specifically, except for JYH007, the other four compounds did not have any Ames toxicity, which is an indicator of carcinogens and mutagens. Additionally, the maximum tolerated dose is generally used for predicting the toxic dose threshold of compounds in the human body, and phase I clinical trials often utilize it as a reference for the starting dose. The maximum tolerated dose of the five compounds was estimated as “high” (>0.477 log(mg/kg/day)). Thirdly, all five compounds presented no hepatotoxicity, which is the primary safety parameter for drug discovery. Furthermore, for the acute toxic prediction, HQA004 received the highest oral rat lethal dosage values (2.466 mol/kg), followed by QHA013 (2.446 mol/kg), HQA016 (2.26 mol/kg), JYH010 (2.166 mol/kg), and JYH007 (1.904 mol/kg). Finally, pkCSM also utilized the lethal concentration values for acute toxicity prediction. The results showed that JYH007 and JYH010 may have high acute toxicity as their lethal concentration values are lower than 0.5 mM (log lethal concentration values <−0.3), whereas the other three compounds have low acute toxicity. Based on the ADME/T properties of the five candidate compounds, we selected HQA004, HQA016, and QHA013 for subsequent analyses, since they have good medical chemistry characteristics and low toxic properties. For JYH007 and JYH010, further compound modifications may be required since they were predicted to be highly toxic compounds with poor pharmacokinetics.

### 2.3. Preliminary Molecular Dynamics Simulations Indicated Potential Synergistic Effects of HQA004, HQA016, and QHA013 with Arbidol Within 50 ns

**Figure 2:**
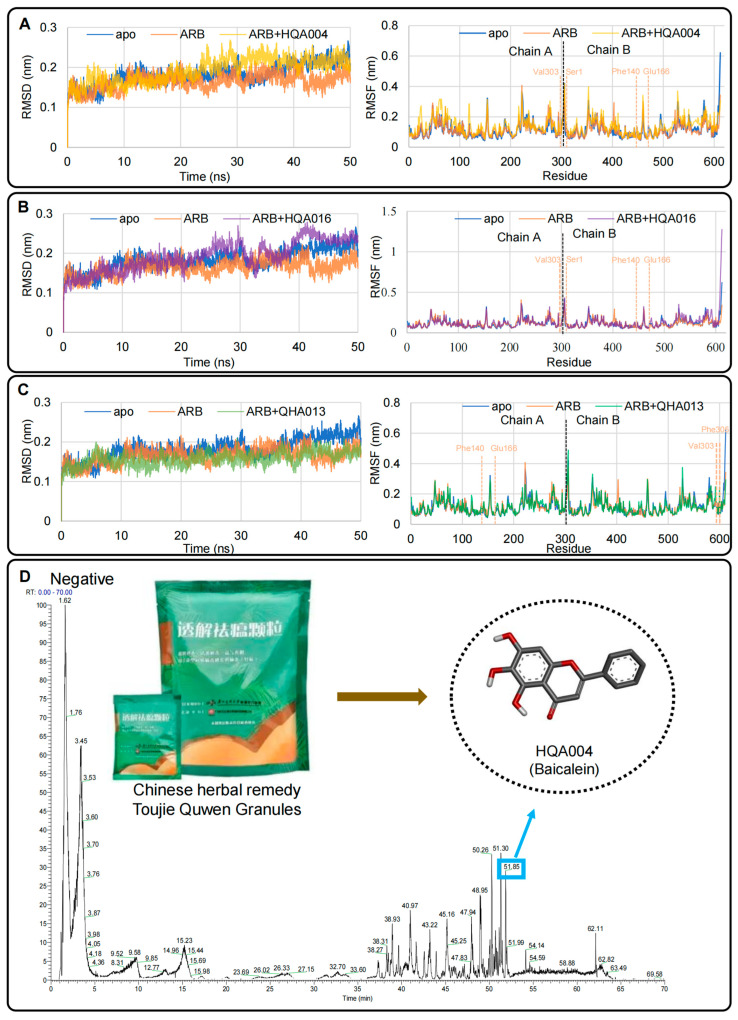
Preliminary molecular dynamics simulations of HQA004, HQA016, and QHA013, and metabolomics profiling for the Toujie Quwen Granules. (**A**–**C**) Root mean square deviation of backbone Cɑ atoms (RMSD) and root mean square fluctuation (RMSF) values of the three compounds. (**D**) Negative mode of untargeted metabolomics and the peak of HQA004 (baicalein). Corresponding compound names are provided in Table S2.

We conducted 50 ns all-atom molecular dynamics simulations on the SiBioLead server for the five complexes, including M^pro^ *apo*, ARB-M^pro^, HQA004-ARB-M^pro^, HQA016-ARB-M^pro^, and QHA013-ARB-M^pro^, to develop a preliminary idea of whether these three candidate compounds may have synergistic effects with ARB. Figure 2A–C shows the Root mean square deviation of backbone Cɑ atoms (RMSD) data and root mean square fluctuation (RMSF) results of all the complexes. We observed that all simulation structures reached equilibration at around 10 ns. For RMSD, major displacements of more than 0.3 nm in all the systems were not observed. Although small fluctuations can be detected after 10 ns, all the complexes can be considered as equilibrated. In terms of the average RMSD from 10 to 50 ns, HQA016-ARB-M^pro^ received the highest values (0.204 nm), followed by HQA004-ARB-M^pro^ (0.202 nm), M^pro^ *apo* (0.19 nm), ARB-M^pro^ (0.17 nm), and QHA013-ARB-M^pro^ (0.16 nm). Specifically, except for QHA013, when the herbal compounds were added to the ARB-M^pro^ structure, their RMSD was slightly higher than that of ARB-M^pro^. The average RMSD of these two herbal compounds, HQA004 and HQA016, was closer to M^pro^ *apo* (0.012 nm and 0.014 nm, respectively), compared to the ARB (0.02 nm) and QHA013 (0.03 nm), which means that HQA004 and HQA016 complexes seem to be more stable than the ARB-M^pro^ complex. However, it is important to note that the differences in RMSD between these three compounds and ARB monotherapy were smaller than 0.05 nm.

Regarding the RMSF, we closely observed the active binding sites (Ser1, Phe140-Glu166, Val303-Phe305) (Figure 2A–C). In the corresponding areas in either chains A or B indicated in the figures, the peaks of the three herbal compound systems were similar to ARB and *apo*. Most of the peaks in these regions can also be considered to overlap with each other, which means there are no major displacements found among those structures. Concurrently, most of the time, when the herbal compounds were added to the ARB-M^pro^ complex, the whole system appeared more likely to display the same behavior as the *apo* one, which can be physically observed from Figure 2A–C. Based on the 50 ns simulation data, we believe that these candidate compounds may have synergistic effects with ARB for M^pro^. Nevertheless, it is worth noting that it is still too early to conclude, as 50 ns simulations were not enough to build the whole story.

### 2.4. Untargeted Metabolomics Confirmed HQA004 (Baicalein) Was the Only Active Component Identified from the Mixture of Toujie Quwen Granules Among the Three Candidates

To explore the actual compounds of TQG, we employed the ultra-performance liquid chromatography-quadrupole-exactive-mass-spectrometry (UPLC-Q-Exactive Orbitrap-MS) combined with the data-dependent acquisition method. As a result, we identified 155 components from the negative ion peak and 319 components from the positive ion peak (Figure 2D, and Supplementary Material Figure S1 and Data S1). We then compared the mass spectrometry results with TQG data in the Traditional Chinese Medicine Systems Pharmacology Database and Analysis Platform (TCMSP) [42]. We found that 10 components in the positive ion peak were identified, namely kaempferol (LQA019, PubChem CID: 5280863), luteolin (LQA021, JYH022, QHA010 and TZS005, PubChem CID: 5280445), formononetin (HQB012, PubChem CID: 5280378), quercetin (LQA023, JYH023, CHA017, QHA022, QHB024, WMA008 and HQB020, PubChem CID: 5280343), indigo (DQY002, PubChem CID: 10215), skullcapflavone II (HQA015, PubChem CID:124211), isovitexin (DQY009, PubChem CID: 162350), artemetin (DQY009, PubChem CID:5320351), xyclopamine (CBM005, PubChem CID: 442972), and artemisinin (QHA019, PubChem CID:68827). Four components in the negative ion peak were found, including luteolin, quercetin, baicalein (HQA004, PubChem CID: 5281605), and formononetin. This helps explain the substance basis of the drug effect, and we therefore focused more on the compounds identified by this step in the next studies.

### 2.5. Extended Molecular Dynamics Simulations Elucidated Synergism Between Herbal Compound HQA004 (Baicalein) and Arbidol: Proposed Synergistic Mechanisms

Since HQA004 (baicalein) was the sole compound identified as present in TQG using metabolomics, we focused on this compound because it is the most likely to be capable of acting as a synergistic partner for ARB among the identified components. Extended MD simulations were performed to provide further insights into whether HQA004 may enhance ARB interactions with M^pro^ over a longer period. Specifically, we examined the influence of different stoichiometric ratios and proposed binding mechanisms of HQA004 to the ARB-M^pro^ complex on both the *ARB ligand* (its binding affinity and motions within the binding pocket) and the effects on M^pro^ itself in terms of structure and motions.

**Figure 3:**
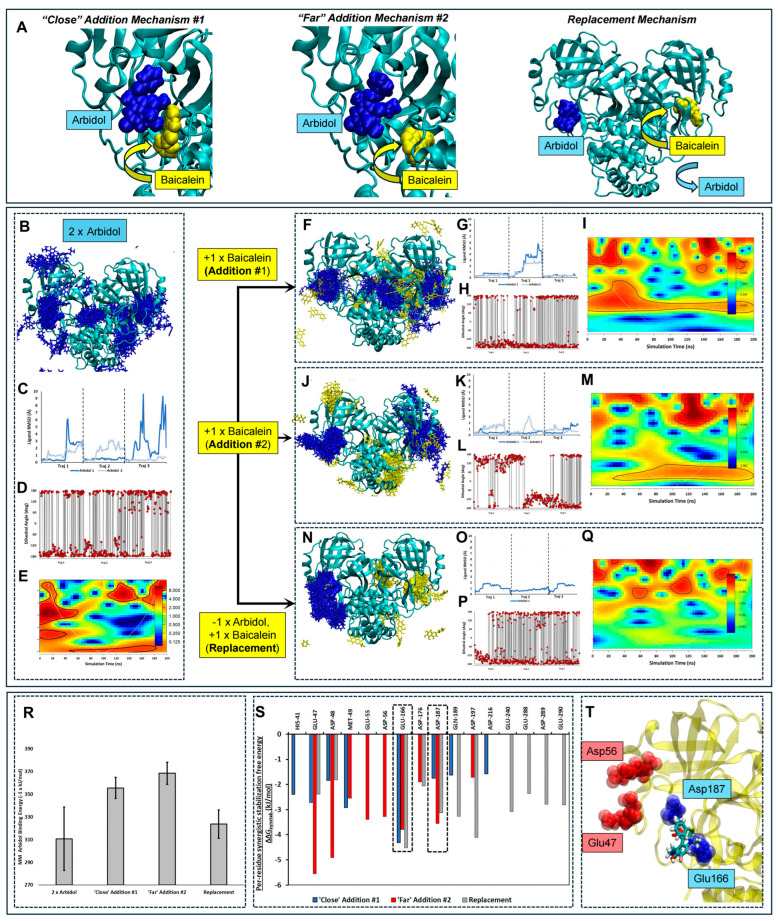
Extended molecular dynamics simulations of M^pro^ bound with Arbidol alone and in the presence of HQA004. (**A**) Initial proposed synergistic binding mechanisms of HQA004 (yellow spheres) to arbidol (blue spheres). (Left) *“Close” Addition Mechanism #1*, where HQA004 is initially docked in close proximity to ARB, forming several inter-atomic contacts. This pose is adapted directly from the automated docking study described above. (Middle) *“Far” Addition Mechanism #2*, where HQA004 is initially docked at a slightly more distant position compared to the first proposed mechanism, and (Right) *Replacement Mechanism,* where ARB and HQA004 bind to the two known active sites on separate monomers. (**B**–**E**) Analyses for the Arbidol-only simulations, showing a graphical representation of the diffusional behavior of arbidol (blue) (**B**) with snapshots taken over all MD trajectories; Arbidol ligand RMSD (**C**), Arbidol dihedral angle with respect to time and trajectory (**D**), and wavelet power spectrum of inter-monomer contacts for a single representative trajectory (**E**). Similar datasets are shown for the *“Close” Addition* (**F**–**I**), *“Far” Addition* (**J**–**M**), and *Replacement* (**N**–**Q**) mechanisms of HQA004-mediated synergy. (**R**) Molecular mechanics binding energy for Arbidol under the four stoichiometries modelled. (**S**) Per-residue synergistic stabilization free energy (ΔΔGsynstab) for residues in the vicinity of Arbidol, and (**T**) graphical representation of the main residues involved in the HQA004-mediated stabilization of Arbidol.

M^pro^ is a dimeric complex and offers two possible nominally identical active site pockets that may bind ligands. Here, we examined the binding of ARB alone to the available M^pro^ binding pockets, as well as the addition of the proposed synergistic ligand, HQA004, to support ARB. We explored three possible synergistic mechanisms as follows:

- Figure 3A (left) shows *“Close” Addition Mechanism #1*, where HQA004 is initially docked in close proximity to ARB, forming several inter-atomic contacts. This pose is adapted directly from the automated docking study described above. This pose corresponds to a 2:1 stoichiometric ratio of ARB to HQA004.
- Figure 3A (center) shows *“Far” Addition Mechanism #2*, where HQA004 is initially docked at a slightly more distant position compared to the first proposed mechanism. This mechanism was explored to predict whether a greater synergism could be achieved if a supporting ligand was close enough to produce additional stability to the binding site while avoiding direct close-contact atomic clashes, which may paradoxically destabilize the main ARB ligand. Our previous manuscript on a similar approach suggested that a nominally synergistic ligand may interact repulsively with the main ligand [8,17]. This pose also corresponds to a 2:1 stoichiometric ratio of ARB to HQA004.
- Figure 3A (right) shows the *Replacement Mechanism* where ARB and HQA004 bind to the two known active sites on separate monomers; thus, the supporting ligand is proposed to displace a previously bound ARB. This pose enables the exploration of the impact of HQA004 binding at an opposing monomer on the stability of ARB. This pose corresponds to a 1:1 stoichiometric ratio of ARB to HQA004.

In the following, we focus on comparing the behaviors of ARB and M^pro^ in the absence and presence of HQA004 under the three proposed synergistic mechanisms described.

### 2.6. HQA004 (Baicalein) Stabilizes the Binding of Arbidol by Reducing Its Diffusion and Intramolecular Conformational Shifts

Several lines of computational evidence from previous steps strongly suggest that the presence of HQA004 increases the binding stability of ARB. Figure 3C,G,K,O show the RMSD plots of ARB with respect to time, fitted to the entire M^pro^ complex, which enables the visualization of the diffusional behavior of the ligand. In the *absence* of HQA004 (molecular representation shown in Figure 3B), ARB undergoes substantial diffusional motion, as shown by the spread of positional clusters (overlays of positions where ARB was located during the simulations) over the entire M^pro^ structure. The RMSD plot (Figure 3C) also indicates sharp peaks, showing high, abrupt movements related to unbinding and re-binding of the two ARB ligands. In contrast, the addition of HQA004 close to ARB (Figure 3F) results in partial stabilization, with only a single RMSD peak (Figure 3G) indicative of a single unbinding and rebinding event during the trajectories. There are also fewer, and more densely packed, positional clusters, indicative of higher ARB stability. Even more remarkably, both the *“Far” Addition Mechanism #2* (Figure 3J) and *Replacement Mechanisms* (Figure 3N) result in substantial stabilization of ARB, with both mechanisms resulting in low ligand RMSD values (Figure 3K and Figure 3O, respectively), and the formation of only a single positional cluster centered at the M^pro^ active site, indicative of persistent binding. Based on the relatively low RMSD value, the *“Far” Addition Mechanism #2* (Figure 3K, dark blue curve) is likely to lead to the most effective stabilization of ARB binding. The presence of HQA004 marginally reduces the conformational flexibility of ARB within the ARB-M^pro^ complex, which is not unexpected, as the supporting ligand was already shown to restrict its diffusion. The variation time series are shown for the central C-C-C-S dihedral angle for ARB in the absence (Figure 3D) and presence (Figure 3H,L,P). ARB alone (Figure 3D) exhibits frequent and rapid rotation and interconversion between 180 and −180°. The presence of HQA004 in the *“Close” Addition Mechanism #1* and *Replacement Mechanisms* (Figure 3H and Figure 3P, respectively) results in a moderate reduction in dihedral angle switching, with most of the conformations at −180°. Most striking of all is the *“Far” Addition mechanism #2* (Figure 3L), which almost fully restricts the dihedral rotation of ARB, with the angle being almost exclusively 180° in half of the series of trajectories, and then undergoing only a single conformational switch to −180° during these simulations. Therefore, consistent with the diffusional RMSD data described above, the *“Far” Addition Mechanism #2* is most capable of stabilizing ARB in terms of diffusional motions, as well as constraining its internal dynamics. This latter point may mean that when HQA004 is present at a moderate distance, ARB is more capable of retaining a structure that enables it to form persistent contact with the surrounding M^pro^ residues.

### 2.7. A 1:1 Ratio of HQA004 and Arbidol Decouples the Movements Between Monomers and Results in Higher Frequency Structural Oscillation

To determine whether the addition of HQA004 affects the overall structure and dynamics of the M^pro^ dimer (which may have implications on its function as well), we calculated the total number of inter-atomic interactions between each monomer with respect to time and performed wavelet transform analysis to highlight any periodic behaviors that differ between the HQA004-free and HQA004-bound M^pro^. Figure 3E,I,M,Q show the wavelet power spectra for all four systems. In the *absence* of HQA004, the ARB-M^pro^ complex exhibits substantial low-frequency (high amplitude) motion with respect to inter-subunit contacts (Figure 3E) throughout the simulation timeframe; the distance between the two monomers oscillates slowly in a “breathing”-like motion, suggestive of tight dynamical coupling between the monomers. The addition of HQA004 on or near ARB results in a moderate increase in the inter-monomer oscillation frequency (Figure 3I,M), suggestive of a slight disruption in this inter-monomer motional coupling. In stark contrast, the Replacement mechanism results in the complete loss of low-frequency inter-monomer oscillation (Figure 3Q), with only high-frequency periodic motion present in scattered segments of the trajectory, suggestive of almost full uncoupling between the movements of the two monomers. Overall, the addition of HQA004 disrupts the coupling of inter-subunit motions in a manner that depends sensitively on the supporting ligand’s binding position and stoichiometry.

### 2.8. HQA004 (Baicalein) Promotes Arbidol Interaction with Acidic M^pro^ Residues in a Binding Position-Dependent Manner

HQA004 increases the binding free energy of ARB to M^pro^ via specific acidic residues. Figure 3R shows a bar chart comparing the molecular mechanics binding free energy of ARB by itself and in the presence of HQA004 under the three different mechanisms proposed. The mere presence of HQA004 results in an increase in binding affinity, with the *Replacement mechanism* weakly enhancing the interaction energy (by ~3%), not unexpected as this is a long-distance effect. Consistent with the observations above, the *“Far” Addition Mechanism #2* results in the highest increase in binding energy relative to the simulations of the HQA004-free system (by 20%). The specific residues that contribute to this additional stabilization are shown in the binding free energy decomposition chart shown in Figure 3S. To more clearly facilitate comparison with the “baseline” HQA004-free simulation, we calculate the change in binding free energy of ARB as a result of adding baicalein, which we term the “synergistic stabilization energy” (ΔΔG_synstab_). A more negative value at a given residue indicates a greater increase in binding strength to ARB due to the presence of HQA004. Each synergistic mechanism produces a distinct ΔΔG_synstab_ profile. However, the two residues that show significantly increased binding affinity to ARB regardless of synergistic mechanism are Glu166 and Asp187, shown as blue spheres in Figure 3T. These acidic residues are proposed to be key to HQA004’s synergistic enhancement properties, at both ARB:HQA004 stoichiometries explored. Also consistent with the foregoing discussion, the *“Far” Addition Mechanism #2* results in the highest per-residue contribution to ARB stabilization at Glu47 and Asp48 (red spheres in Figure 3T). These residues are near the main active site, providing further evidence that the presence of a synergistic supporting ligand may play its role by shifting or distributing the burden of providing binding free energy towards other residues in the neighborhood of the main active site. Interestingly, while both “Addition” mechanisms tend to enhance ΔΔG_synstab_ within the more N-terminal segments His41-Asp56 and Glu166-Asp216, the *Replacement Mechanism* distributes the free energy contribution amongst the more C-terminal residues from Asp197-Glu290. Therefore, the specific residues involved in enhanced binding to ARB depend strongly on the binding site of the supporting ligand HQA004.

### 2.9. Principal Component Analysis and Free Energy Landscape Analyses Reveal HQA004 (Baicalein) Binding Enhances M^pro^ Dimer Rigidity and Stability

**Figure 4:**
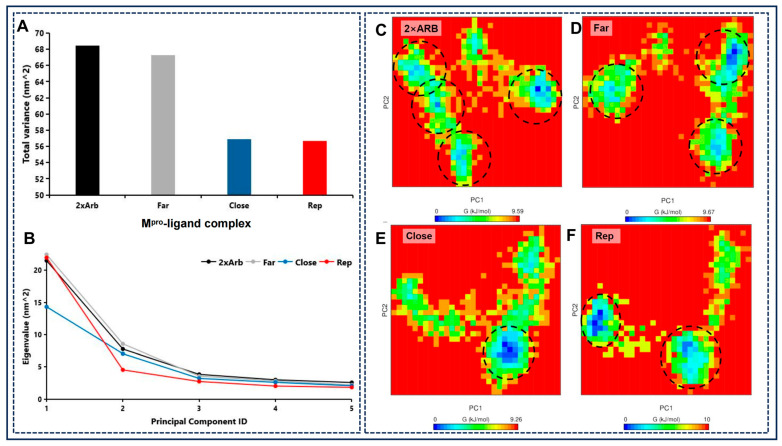
Principal component analysis and free energy landscape analyses of M^pro^ bound with Arbidol alone and in the presence of HQA004. (**A**) Total structural variance of the four complexes. (**B**) Eigenvalues of the first five principal component vectors. (**C**–**F**) Free energy landscape analyses plots of the four complexes.

In order to decrease the dimensionality of the data, identify key vibrational modes, and visualize the main conformational transitions during the simulations, we conducted a principal component analysis (PCA)-based free energy landscape (FEL) analysis of the simulation trajectories (Figure 4).

The total structural variance for each of the four complexes is shown in Figure 4A. The 2× ARB-only and *“Far” Addition Mechanism #2* ARB+HQA004 M^pro^ complexes exhibit the highest variance values (68 and 67 nm^2^, respectively), indicating that both have high conformational flexibility. In stark contrast, both the *“Close” Addition Mechanism #1* ARB+HQA004 and *Replacement Mechanism* ARB+HQA004 complexes exhibit relatively low variance (both ~56 nm^2^, approximately 17% lower), indicating substantially lower flexibility.

Eigenvalues with respect to the *“Far” Addition Mechanism #2* to each of the first five PC vectors are shown in Figure 4B, while PCs above five contain increasingly negligible contributions. In all cases, PC1 captures most of the structural variation, while PC2 represents the second largest variation. Consistent with the total variance described above, the *“Close” Addition Mechanism #1* M^pro^ complex has the lowest PC1 eigenvalue (14 nm^2^), indicating the lowest amplitude major concerted motion amongst the complexes, while the *Replacement Mechanism* M^pro^ complex has the lowest PC2 eigenvalue. Furthermore, the PC2 eigenvalue for the *“Close” Addition Mechanism #1* M^pro^ complex is still substantially high (~7 nm^2^) relative to PC1, indicating that the addition of Baicalein in a close position to ARB tends to induce more complex, multimodal dynamics more evenly spread over the principal components.

To support the eigenvalue analysis and gain further insights, Free Energy Landscape (FEL) representations enable an intuitive visualization of the flexibility and number of distinct conformations sampled during the simulations by mapping the Gibbs free energy as a function of PC1 and PC2, which, together, capture most of the structural variations in the simulations. The FEL plots for each of the four systems are shown in Figure 4C–F. Consistent with the variance and eigenvalue analyses, the 2× ARB (Figure 4C) and *“Far” Addition Mechanism #2* (Figure 4D) complexes exhibit up to four distinct conformational clusters, highlighted by the deep blue regions corresponding to deep free energy minimum wells. In contrast, the *“Close” Addition Mechanism #1* complex (Figure 4E) exhibits only a single, well-defined deep free energy well. Therefore, the binding of HQA004 near ARB stabilizes M^pro^ in essentially a single well-sampled conformation. Similarly, the *Replacement Mechanism* complex (Figure 4F) exhibits only a small number (two) of well-defined free energy wells.

### 2.10. Luciferase Assays and Surface Plasmon Resonance Approach Clearly Reflected the Synergistic Effects of Arbidol and HQA004 (Baicalein) for Multiple Combination Ratios

**Figure 5:**
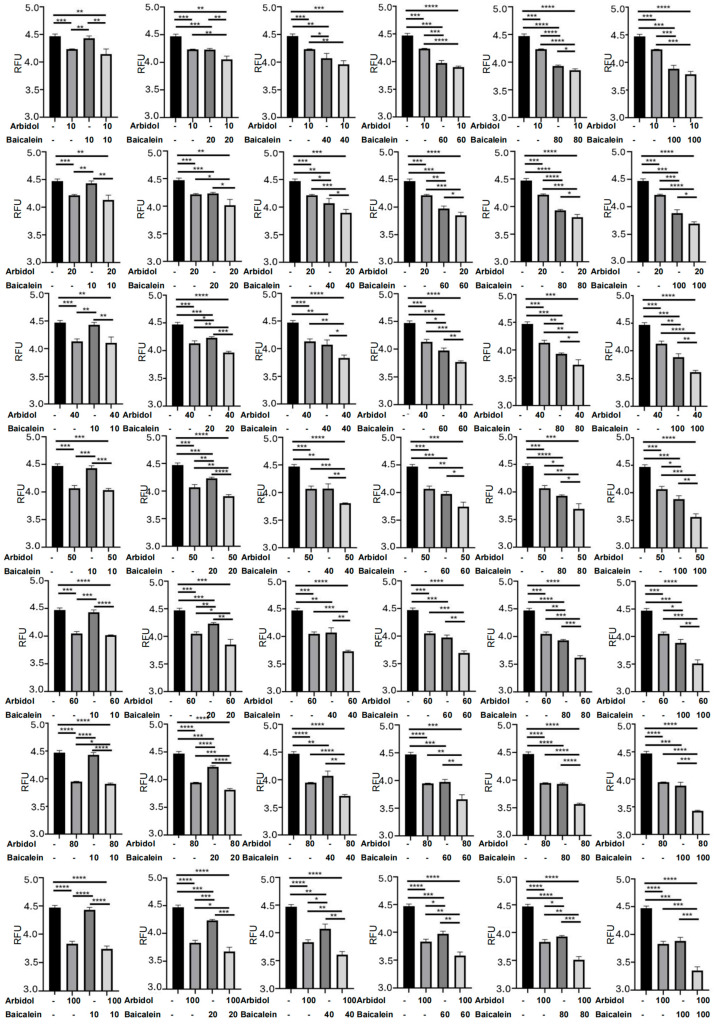
Luciferase assays of the 42-combination ratio between arbidol and HQA004 (baicalein). RFU: Relative Fluorescence Unit. The units on the *x*-axis are IC_50_, representing the concentrations of arbidol and HQA004 (baicalein). The concentrations for ARB were 10, 20, 40, 50, 60, 80, and 100 times IC_50_ values and were equal to 160 μM, 320 μM, 640 μM, 800 μM, 960 μM, 1280 μM, and 1600 μM. In the meantime, 10, 20, 40, 60, 80, and 100 times IC_50_ values of HQA004 were set and equal to 31.2 μM, 62.4 μM, 124.8 μM, 187.2 μM, 249.6 μM, and 312 μM. *: *p* < 0.05; **: *p* < 0.01; ***: *p* < 0.001; ****: *p* < 0.0001.

**Figure 6:**
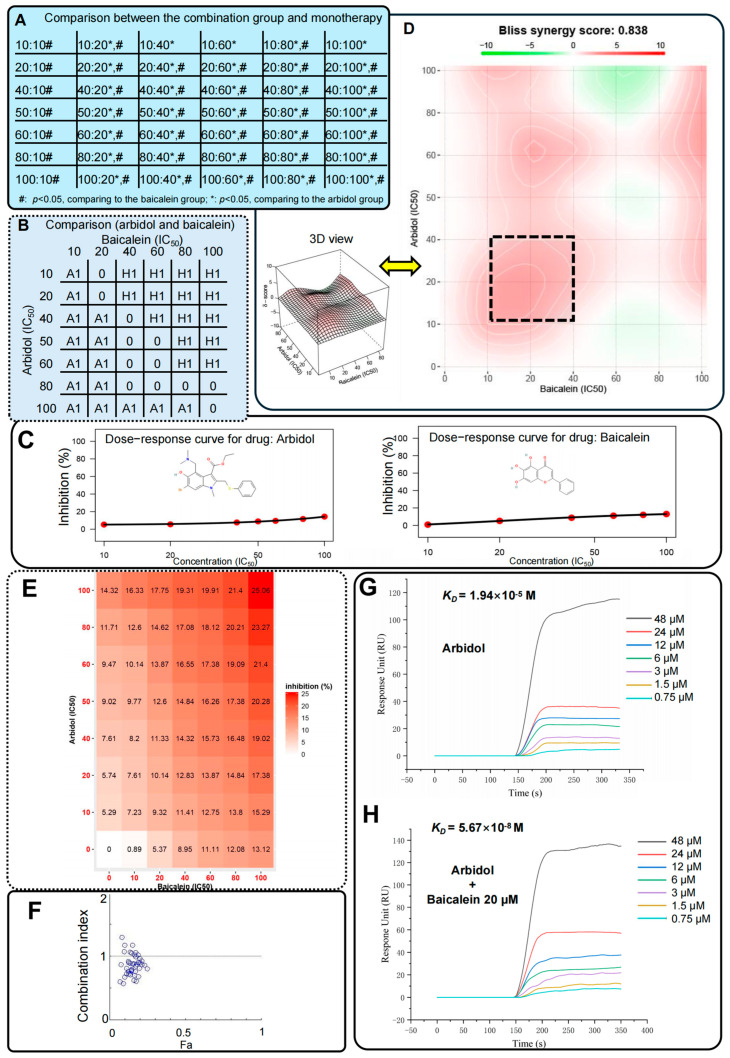
Synergistic effects evaluation and interaction verification of arbidol and HQA004 (baicalein). (**A**) Statistical calculations for the 42-combination ratio (comparison between the combination group and monotherapy). #: *p* <0.05, comparing to the HQA004 group; *: *p* <0.05, comparing to the arbidol group. (**B**) Statistical calculations for the 42-combination ratio (comparison between arbidol and HQA004 monotherapy). A1: The arbidol group received better inhibition effects than the HQA004; H1: The HQA004 group received better inhibition effects than the arbidol group. (**C**) Dose-response curve for arbidol and HQA004 monotherapy. (**D**) Synergistic map and most synergistic areas for the combination of arbidol and HQA004. (**E**) Heatmap of the inhibition rate for monotherapy and combination therapy. The darker the red, the higher the inhibition rate. (**F**) Combination index of the combination of arbidol and HQA004. (**G**,**H**) The surface plasmon resonance approach validated the synergistic interactions between arbidol and HQA004.

In order to validate our computational calculations, we employed luciferase approaches to investigate the synergistic mechanisms between ARB and HQA004 (Figure 5 and Figure 6A–C, and Table S6). All data generated by the assays met the requirements for using One-way ANOVA and unpaired Student’s *t*-test. Firstly, dose-dependent responses were detected for both of the monotherapy groups, and both of the drugs reduced the enzyme activities (Figure 6C). Specifically, in comparison to the control group (i.e., the ARB-only group), statistical significance was detected even at the lowest dosage of ARB (10 IC_50_, 160 μM), whereas statistical significance was observed from 20 IC_50_ (62.4 μM) to the highest dosage (100 IC_50_, 312 μM) in the HQA004 group. Additionally, we compared the treatment effects between the monotherapy groups with various concentrations (Figure 6B). As we can see in Figure 6B, “A1” represents that the ARB group received better inhibition effects than the HQA004, while “H1” stands for better treatments of the HQA004 group, compared to the ARB group. The “0” in the figure indicates that there was no difference between the two monotherapy groups for the specific dosage.

We intended to see whether ARB and HQA004 produced synergistic effects using multiple combination ratios, and therefore, we set up a total of 42 combination ratios between ARB and HQA004 (Figure 5). Based on the data, we found that all the combination groups significantly reduced the enzyme activity, compared to the control group, even at the lowest combination dosage (ARB: 10 IC_50_, 160 µM; HQA004: 10 IC_50_, 31.2 µM). We also compared the treatment effects of the combination group to the two monotherapy groups, which used the same treatment dosage as the corresponding drugs in the combination group (Figure 6A). We observed that the combination group produced better treatment effects than the HQA004-only groups, except for three of the combinations (ARB-HQA004: 10 IC_50_-40 IC_50_; 10 IC_50_-60 IC_50_; 10 IC_50_-100 IC_50_), whereas compared to the ARB-only groups, almost all of the combination groups significantly increased the inhibition rate, except for the one utilizing 10 IC_50_ ARB in their combinations.

To investigate whether ARB and HQA004 have synergistic effects, two popular calculation models, the Bliss synergy score (SynergyFinder) and combination index (CI) (CompuSyn), were utilized (Figure 6D–F and Table 1). In terms of the Bliss synergy score, a total of 33 combination groups (78.6%) showed synergism between two drugs, and only 9 groups may have antagonistic actions (ARB-HQA004: 10 IC_50_-60 IC_50_; 10 IC_50_-80 IC_50_; 50 IC_50_-60 IC_50_; 50 IC_50_-80 IC_50_; 80 IC_50_-40 IC_50_; 80 IC_50_-60 IC_50_; 100 IC_50_-40 IC_50_; 100 IC_50_-60 IC_50_; 100 IC_50_-80 IC_50_). Concurrently, for the CI calculations (Figure 6F), there were also 33 combination groups (78.6%) that had a CI lower than 1, which means that synergistic effects may show in these groups. In contrast, only nine groups may present antagonistic effects since their CI values are higher than 1 (ARB-HQA004: 10 IC_50_-80 IC_50_; 10 IC_50_-100 IC_50_; 20 IC_50_-80 IC_50_; 20 IC_50_-100 IC_50_; 40 IC_50_-10 IC_50_; 40 IC_50_-80 IC_50_; 40 IC_50_-100 IC_50_; 50 IC_50_-10 IC_50_; 60 IC_50_-10 IC_50_). Furthermore, 25 combination groups (59.5%) may have synergistic effects with strong evidence since they all received a value that indicated synergism for both of the models (Bliss synergy score >0 and CI <1). Additionally, SynergyFinder indicated the most synergistic area. As Figure 6D shows, the most synergistic area (MSA) covered four of the combinations (ARB-HQA004: 20 IC_50_-20 IC_50_; 20 IC_50_-40 IC_50_; 40 IC_50_-20 IC_50_; 40 IC_50_-40 IC_50_). These four groups also produced a synergistic value in both the Bliss synergy score and CI.

To produce more evidence for the synergism between ARB and HQA004 against the main protease, we used SPR sensors for validation. In this study, we fixed the M^pro^ protein in the chip, and then we flowed ARB and ARB plus HQA004 through the chip to test the binding affinity, respectively. The results of SPR are shown in Figure 6G–H. The *K_D_* value of ARB monotherapy was 1.94 × 10^−5^ M. Encouragingly, when we combined ARB with HQA004, the *K_D_* value decreased significantly to 5.67 × 10^−8^ M. Based on the data from the SPR approach, we can confirm the essential role of the herbal compound HQA004, since it significantly increased the binding affinity of the complex structure, compared to the model without HQA004. The SPR results also validated the findings from the luciferase assays. When the number of molecules of ARB is higher than that of HQA004, it will, to a certain extent, produce better therapeutic effects.

## 3. Discussion

For a considerable time, the traditional pharmaceutical approach to medication and treatment strategies has been characterized by the “one drug, one target, one disease” paradigm [43]. With the development of pharmaceutical sciences, this conventional approach has progressively changed to combination therapies over the last decade. Due to the recently identified disadvantages of monotherapy, for example, the limited therapeutic effects of monotherapy against chronic health conditions, the appearance of drug resistance, and the side effects of synthetic mono-medications, combination therapy is gradually gaining attention [44]. Increasing evidence suggested that some diseases that are difficult to treat via a monotherapy approach, for example, cancer, diabetes, and acquired immune deficiency syndrome, could be better treated using combination therapy [45,46]. A strong recent example is Pfizer’s highly regarded antiviral agent, Paxlovid (nirmatrelvir+ritonavir), for patients who are suffering from mild to moderate symptoms of COVID-19. The main ingredient in this drug is nirmatrelvir, and its effects were simulated by ritonavir, which is a small molecule. Clinical evidence also supports the success of the development of the drug, since it can lead to a more positive outcome for those at high risk of severe COVID-19 [47].

In China, the integration of TCM and Western medicine began in the early 20th century and has progressed to become an important therapeutic method in the national health system since the 1970s [48]. This combination treatment model has been developed based on the rich history and knowledge of TCM, as well as the scientific rigor and technological advancements of Western medicine [12]. A significant amount of evidence has shown that the integration of TCM and Western medicine will lead to greater therapeutic benefits than a monotherapeutic approach of Western medicine, for example, by better therapeutic effects with fewer adverse events, especially in COVID-19 treatment [49]. The previously published randomized controlled trial indicated the therapeutic effects of ARB combined with TQG, which can significantly increase the total effective rate, compared to ARB monotherapy [36]. Therefore, we employed this strategy to focus on the synergy between Western medications and herbal compounds for developing combination drugs.

The current research proposed to seek the synergy between Western medication ARB and herbal compounds from TQG, which is a recommended herbal remedy designed for the management of COVID-19 treatment. We also aimed to investigate whether the herbal compounds can enhance the treatment effects of ARB. Previously, we utilized a comprehensive approach called the SMMM approach for the synergy study [8,17], and at this point, we extended the applications of this technique for the development of novel combination drugs, which include approved drugs and herbal compounds. Firstly, we identified five herbal compounds, HQA004, HQA016, JYH010, JYH007, and QHA013, in six conformations. These herbal compounds were observed to bind physically close to ARB. Based on our synergy theory from our previously published paper, if a compound physically interacts and is located close to the proposed ligand, this compound may produce synergistic effects with the proposed ligand [8,17]. Thus, docking results suggested that these five compounds may have synergistic effects with ARB. Subsequently, we identified that only HQA004, HQA016, and QHA013 appeared to have good pharmacokinetic properties in certain parameters, including PAINS, solubility, intestinal absorption rate, total clearance, and drug-likeness evaluation. Additionally, these three compounds also passed our toxicity filters, including the Ames toxicity, maximum tolerated dose, hepatotoxicity, oral rat lethal dosage, and acute toxicity prediction. Pharmacokinetic and toxicity screening is an important step for drug development since it can increase the success rate for developing a drug. Considering that these three compounds passed these parameters, it is reasonable to allocate them to the priority group for subsequent study. Although JYH010 and JYH007 failed the test, these compounds could also be considered after compound modifications. Next, we conducted a 50 ns simulation for these three compounds combined with ARB and M^pro^ and found that the HQA004-ARB-M^pro^ complex and HQA016-ARB-M^pro^ were more stable than the ARB-M^pro^ complex, since the RMSD and RMSF behaviors of these herbal compound-drug-protein systems were closer to the behavior of the protein *apo* structure, compared to the ARB-M^pro^ model. To see whether these three compounds existed in the herbal remedy, we conducted a UPLC-Q-Exactive Orbitrap-MS for the TQG mixture. Specifically, we only found HQA004 in the herbal remedy TQG via the untargeted metabolomics. Subsequently, we selected HQA004 as our priority for further simulations and analyses. The SPR approach supported this finding since the *K_D_* value of the combination of ARB and HQA004 was much lower than the *K_D_* value of ARB monotherapy, indicating that synergism existed between ARB and HQA004, reflecting the high binding affinity. For HQA016 and QHA013, these two compounds still have potential for the development of a combination drug with ARB; however, considering they could not be found in the mixture, we will investigate their effects with ARB, respectively, in the future.

To understand how the synergy occurred between ARB and HQA004 in M^pro^, we extended the simulation to a longer period. Because M^pro^ is a dimer with one active binding pocket in each of the chains, we also considered the impacts of different stoichiometric ratios of ARB and HQA004 in the protein. Based on the docking results, we hypothesized that there were three synergistic mechanisms, including the *“Close” Addition Mechanism #1*, the *“Far” Addition Mechanism #2*, and the *Replacement Mechanism*. Among the three potential mechanisms, we believed that the most effective stabilized system of ABR binding is the *“Far” Addition Mechanism #2* because of its low RMSD value. We suggested that the reason behind this result is that HQA004 can stabilize ARB by restricting ARB diffusion and limiting its internal dynamics. Additionally, we calculated the ΔΔG_synstab_ value to gain further insights into synergism and the MM-PBSA results, recommending that the reason why HQA004 can enhance the interactions of ARB with the protein is due to the specific residues of M^pro^. Wavelet analysis also supported the *“Far” Addition Mechanism #2*. Results showed that synergism may occur when the stoichiometric number of ARB is higher than HQA004, as only a moderate rise in the inter-monomer oscillation frequency was detected. On the contrary, when the stoichiometric number of ARB is equal to HQA004, the phenomenon of full uncoupling between the movements of the two chains was found.

Looking at the data generated from the luciferase assays, it is clear that the combination groups significantly reduced the enzyme activity, compared to the control group and monotherapy groups. It is also essential that two-thirds of the combinations appeared synergistic in this analysis. It should be noted that the calculation in these models was based on mathematical algorithms, meaning that if a value is in the synergistic area, there is a higher probability of having synergistic treatment effects for the specific combination. Furthermore, the purpose of the MSA calculations is to identify an area with ideal synergism and a lower drug dosage, as a lower dosage may have a lower probability of the incidence of drug adverse events. It is encouraging that the MSA indicated that the number of molecules of ARB was higher than the HQA004 in the specific groups (3:1; 5:1; 10:1), which validated our computational calculations (2:1 stoichiometric ratio of ARB to HQA004). It should be noted that when a drug enters the cells or the human body, it is not necessarily all absorbed and distributed by the cells or the human body, which means that although the ratios identified by the luciferase assays may not be completely the same as our in silico findings, it can still validate our calculations at molecular levels. In other words, synergistic effects may appear only if the stoichiometric number of ARB is higher than HQA004 in the protein structure.

Our work has demonstrated the synergistic mechanisms between Western medicine, ARB, and a herbal compound, HQA004, from a herbal remedy, TQG, against the main protease. This study could be seen as a comprehensive example of the implications of our SMMM techniques for a synergy study, to develop new combination drugs via the theory of integrative TCM and Western medicine. Furthermore, M^pro^ is relatively conservative among coronaviruses, for example, severe acute respiratory syndrome coronavirus and Middle East respiratory virus coronavirus [50], and therefore, the combination of ARB and HQA004 may also be used for other conditions caused by other coronaviruses, which means that they would be developed as pan-inhibitors in the future. Additionally, our research stands out from other ARB combination studies in four main ways. First, we were the first to test theoretically how different ligands work together on the same protein (SARS-CoV-2 M^pro^). Most previous studies only focused on single ligands; nonetheless, we used multi-ligand modeling to design new combination drugs. This is a novel approach for ARB-based therapies. Second, we decoded why these ligands work better together at the molecular level. Using long-term molecular dynamics simulations and wavelet analysis, we observed how baicalein stabilizes ARB by reducing its movement and changing the protein’s shape. Many previous studies did not explain these binding dynamics in detail. Third, our method combines supercomputer-powered multi-ligand docking, free energy calculations, and surface plasmon resonance. Using supercomputers (450,000 times faster than regular ones), we predict results more accurately than ever. Most ARB studies have not used such powerful computational tools. Fourth, we analyzed drug synergy from theoretical chemistry and biophysics angles. We explained how H-bonds, hydrophobic interactions, and protein movements drive synergy, unlike studies that only focus on drug effects. To our knowledge, no prior research has employed this integrated computational model combining TCM-Western medicine theory with multi-ligand biophysics for ARB combination development. Finally, the SMMM approach implicated in this study can also be used to develop novel combination drugs for other diseases other than COVID-19 [17], as well as to explore nanoparticle delivery systems.

However, the present study is inevitably limited by some challenges of computational calculations, for example, result discrepancies in lab tests. It should be noted that a compound with high binding affinity and ideal molecular behaviors may not indicate positive biological activities. For the herbal compound in this study, HQA004 (baicalein), its pharmacological activity for the M^pro^ has been reported many times after the outbreak of COVID-19 [51,52,53]; however, the synergistic effects between HQA004 and other compounds against M^pro^ are unknown. Our previous computational work demonstrated that HQA004 (baicalein) may produce synergistic effects with another compound, cubebin, from the TQG remedy, via allosteric crosstalk between two ligands at a protein target [8]. Secondly, regarding the virtual ADME/T screen, we utilized industry-recognized platforms, for example, False Positive Remover (for PAINS screening) and pkCSM (for ADME/T profiling); nevertheless, computational models rely on training data that may not fully capture rare metabolic pathways or transporter interactions. Therefore, further pharmacokinetics and toxicity studies via animal models are recommended. Thirdly, although we conducted several in vitro experiments to validate our computational calculations, further animal studies are still required to test the synergistic effects of ARB, for example, focusing on viral replication, or using patient-derived cells or organoids, in a biosafety level 3 laboratory. Finally, our study is limited to providing insights into the investigation of synergistic effects, focusing on a single protein. Of course, synergistic effects between two drugs may arise across multiple proteins in the human body, for example, Paxlovid, which combines nirmatrelvir (a direct M^pro^ inhibitor) with ritonavir (which modulates human liver enzymes to prolong drug action). Considering that synergism is complicated, we only provided a solution, based on the SMMM approach, the clinical evidence, and the theory of integration of TCM and Western medicine, for the development of novel combination drugs against the same protein. We also propose that the use of the data generated from this work as the training set to develop an artificial intelligence or machine learning model to thoroughly yet easily investigate the synergism between Western medicine and herbal compounds against M^pro^.

## 4. Materials and Methods

### 4.1. Database Search to Identify Compounds from Toujie Quwen Granules

To establish an herbal compound library for subsequent in silico calculations, we retrieved compounds from our previously published paper that focused on TQG against SARS-CoV-2 [8]. Specifically, all the compounds from TQG from our previous work were retrieved from the TCMSP (https://tcmsp-e.com/) [42]. The TCMSP database is a widely used platform for investigating the systems pharmacology of a herbal formula or a herb for a specific condition. In recent years, this platform has received over 300 citations and involves information on all 499 medicinal herbs listed in the Pharmacopoeia of the People’s Republic of China, including 12,144 herbal compounds [42]. This database also provides data on the pharmacokinetic properties of these compounds, which were manually collected and updated by the developers and maintainers of the platform. The platform was searched utilizing the Chinese pinyin names of the 16 ingredients of TQG as keywords. For the compound selection criteria, oral bioavailability and drug-likeness were set at “≥30%” and “≥0.18”, respectively, based on recommendations from the designers of TCMSP [35]. These pharmacokinetic criteria are chosen since compounds with these characteristics are more likely to possess drug-like properties with significant oral bioactivities. Additionally, duplicate compounds are retained to enable a better understanding of the compound’s mechanisms of action in relation to the herbs from which it originated.

### 4.2. Acquisition of Structures of Identified Ligands

We searched ARB (PubChem CID 131411) and each compound from TQG in the PubChem database (https://pubchem.ncbi.nlm.nih.gov) based on its PubChem CID/SID number to download its 3D structures in standard Simplified Molecular-Input Line-Entry System (SMILES) (SDF file) format [54]. For those without any records in the PubChem database, we downloaded their structures in MOL2 file format from the TCMSP database [42]. Then, we utilized Discovery Studio Visualizer 2019 to convert all compound structures to PDB file format for subsequent calculations [43].

### 4.3. Structural Preparation of the Main Protease of SARS-CoV-2

M^pro^ plays a crucial role in facilitating viral replication and transcription in SARS-CoV-2 [55]. From our previously published work, we believe that M^pro^ is a potential drug target of SARS-CoV-2, because of its functional significance for the virus [56,57]. Moreover, our work also demonstrated the reasons why TQG can be used for the management of COVID-19 because there may be several compound pairs in the formula that could act on the protease of SARS-CoV-2 synergistically [8]. Consequently, we decided to focus on M^pro^ for this study, as an example, to present our novel supercomputing method. We downloaded the structures of M^pro^ (PDB ID: 6LU7; structural resolution 2.16 Å) from the RCSB PDB Protein Data Bank (www.rcsb.org) in PDB file format [55]. Next, we utilized the protein visualization and analysis software Visual Molecular Dynamics (version 1.9.4) to examine and compare the protein structure [58]. We, then, used PyRx GUI (v0.8) to pre-process the protease 3D structure for subsequent docking studies on supercomputers [59].

### 4.4. Multiple Ligands Docking to Investigate Cooperativity Between Arbidol and Compounds from Toujie Quwen Granule

Docking was performed by the National Computational Infrastructure high-performance computing system “Gadi” (24-core Intel Xeon Scalable “Cascade Lake” processors, Canberra, Australia). Potential synergistic interactions were sought between ARB and all ligands from the herbal formula TQG. Since there is no crystal structure of ARB-M^pro^ complex in the RCSB PDB database, we used a conventional single ligand docking approach via AutoDock Vina (v1.1.2) and PyRx GUI (v0.8) on the “Gadi” supercomputer to establish the initial ARB-M^pro^ complex for subsequent calculations. As M^pro^ is a dimer with two chains in it, a “focused” docking on monomer A and monomer B on the protease was performed, respectively. The process enabled the probability of receiving the predicted binding poses of ARB in both chains A and B of M^pro^. Then, Discovery Studio Visualizer 2019 was used to establish a complex of M^pro^ protein with two ARB molecules inserted into the active binding pocket in both chains [43]. Thirdly, to convert the ARB-ARB-M^pro^ complex to the “receptor.PDBQT” files, MGLTools 1.5.6 was utilized [60]. Lastly, Autodock Vina in command line mode was used to conduct dockings between all compounds from TQG to the receptor.PDBQT files [61].

In this docking iteration, the anticipated positions and orientations of the second ligands were projected onto those of the initial ligands (ARB). For the protease, a grid box was established (Supplementary Material Data S2). The dimensions of this box were determined to envelop the entire protein structure and encompass the compounds derived from the botanical constituents earmarked for blind docking experiments. This task was accomplished by utilizing the “maximize box” feature embedded within PyRx. The proteins were maintained in rigid conformations, while the ligands were allowed flexibility. All parameters were maintained at their default settings, with an exhaustiveness value of 48 chosen for all docking runs. The ligands were permitted to adopt various conformations around rotatable bonds, whereas the receptors were held in an inflexible arrangement. Using the molecular docking scoring functions within Autodock Vina, we made predictions about the interaction energy between the primary ligand and the second ligand, together with the protease. This was carried out to foresee and pinpoint supplementary ligands capable of substantially amplifying the binding affinity of ARB [40]. Simultaneously, we opted for the structural examination of second ligands with forecasted binding sites closely aligned with ARB. This structural analysis was performed using Discovery Studio Visualizer 2019, facilitating the visualization of both the 3D structures and 2D representations of interactions between ligands and residues [35]. The count of H-bonds between the second ligands and M^pro^ was computed. Second, ligands that exhibit physical proximity to ARB and establish H-bonds with M^pro^, while also significantly ameliorating the binding affinity of ARB, have the potential to act as collaborative compounds. These compounds could play a role in enhancing the effectiveness of ARB.

### 4.5. Virtual Pharmacokinetic and Toxicity Prediction

The successful advancement of a drug hinges significantly upon comprehending its pharmacokinetics, potential toxicity, and the risk of generating false positive signals as bioactive entities [44]. Herbal compounds physically situated close to the ARB in both chains of the protease were chosen for evaluation through absorption, distribution, metabolism, excretion, and toxicity (ADME/T) predictions. To scrutinize the herbal compounds for the possibility of exhibiting Pan-Assay Interference Compounds (PAINS), which can lead to misleading outcomes in biological screening assays, an online platform called False Positive Remover (http://www.cbligand.org/PAINS/; accessed on 28 August 2023) was employed [45]. Afterward, herbal compounds that exhibited potential as suitable candidates for therapeutic applications underwent an assessment of their ADME/T characteristics using pkCSM (http://biosig.unimelb.edu.au/pkcsm/prediction; accessed on 28 August 2023), as in our previous work [62]. pkCSM is a machine-learning platform designed to predict the pharmacokinetic properties of small molecules, based on 28 regression and classification models that have been trained and tested on various experimental datasets [63]. The ADME/T properties of each identified herbal compound were quantified to assess the compound’s behavior within the human body. We employed ADME/T descriptor functions to predict properties, including aspects such as aqueous solubility, human intestinal absorption, drug-likeness (Lipinski rule), Ames toxicity, human maximum recommended tolerated dose, hepatotoxicity, oral rat acute toxicity (LD_50_), and minnow toxicity.

### 4.6. Metabolomics Profiling to Analyze the Active Components of Toujie Quwen Granules

To investigate whether the candidate herbal compounds identified through database searching and multiple ligand docking are physically present in the herbal mixture of TQG, UPLC-Q-Exactive Orbitrap-MS was employed for the analysis of the active components in TQG. This analysis was conducted in conjunction with a data-dependent acquisition method [37]. The TQG powder (Lot Number: L22A002; Hutchison Whampoa Guangzhou Baiyunshan Chinese Medicine Co., Ltd., Guangzhou, China; Figure S2) was purchased from the Guangzhou Eighth People’s Hospital of Guangzhou Medical University (Guangzhou, China) and was subsequently subjected to UPLC-MS/MS analysis. The sample preparation, extract analysis, metabolite identification, and quantification were conducted at Shanghai Bioprofile Technology Co., Ltd. (Shanghai, China). The sample extracts were analyzed using a SHIMADZU-LC30 UHPLC system (UPLC, Nexera X2 LC-30AD; Kyiv, Ukraine) with a Waters ACQUITY UPLC^®^ HSS T3 chromatographic column (2.1 × 100 mm, 1.8 µm; Prague, Czech Republic). The injection volume was 5 μL, and the column oven was maintained at 40 °C. The flow rate was set to 0.2 mL/min. The mobile phase consisted of solvent A (pure water with 0.1% formic acid) and solvent B (acetonitrile with 0.1% formic acid). The chromatographic gradient elution procedure was as follows: 0–8 min, 0% B; 8–45 min, 0–40% B; 45–50 min, 40–100% B; 50–60 min, 100% B; 60–60.1 min, 100–0% B; 60.1–70 min, 0% B.

Each sample was detected by electrospray ionization in positive ion (+) and negative ion (−) modes, respectively. The samples were separated by UPLC and analyzed by mass spectrometry with a QE Plus mass spectrometer (Thermo Scientific, Waltham, MA, USA; Q Exactive Plus). The ionization conditions were as follows: Spray Voltage: 3.8 kv (+) and 3.2 kv (−); Capillary Temperature: 320 (±); Sheath Gas: 30 (±); Aux Gas: 5 (±); Probe Heater Temp: 350 (±); S-Lens RF Level: 50. MS acquisition was set as follows: Mass spectrum acquisition time: 70 min. The scanning range of the parent ion was 80–1200 *m*/*z*, and the resolution of the first-order mass spectrometry was 70,000 at *m*/*z* 200, AGC target: 3 × 10^6^, Level 1 Maximum IT: 100 ms. Secondary mass spectrometry was acquired as follows: the acquisition of secondary mass of the 10 highest intensity parent ions was triggered after each full scanSpectra (MS2 scan), secondary MS resolution: 17,500 at *m*/*z* 200, AGC target: 1 × 10^5^, two Maximum IT: 50 ms, MS2 Activation Type: HCD, Isolation window: 2 *m*/*z*, Normalized collision energy (stepped): 20, 30, 40.

Raw data were obtained using MS-DIAL software (version 68) for peak alignment, retention time correction, and peak area extraction. The metabolite structures were identified by exact mass number matching (mass tolerance <10 ppm), primary spectral matching (mass deviation masstolerance <0.01 Da) and secondary spectral matching (mass deviation masstolerance <0.02 Da), MS2 fragment similarity score greater than 0.70, search database of TCMSP (https://tcmsp-e.com/; accessed on 23 May 2023), HERB (http://herb.ac.cn/Download/; accessed on 23 May 2023) and SymMap (http://www.symmap.org/; accessed on 23 May 2023) [37].

### 4.7. Molecular Dynamics Simulations and Analyses

To receive an initial understanding of the synergistic interactions between ARB and the three candidate ligands including HQA004, HQA016 and QHA103 identified from previous calculations, we used the MD simulation module on the SiBioLead online molecular dynamics simulation platform (https://sibiolead.com/) (GPU-based high-performance cluster system, Ubuntu OS, NVIDIA GeForce RTX3050 GPUs) to perform a 50 ns preliminary simulations [37]. The PDB files of the *apo*-protein, ARB-M^pro^ complex, and herbal ligand-ARB-M^pro^ complex were uploaded to the server, which was run on GROMACS [64]. We used the default setting on the server for all preliminary simulations. Specifically, the server utilized AMBERTOOLS [65] and ACPYPE [66] to generate the ligand topology automatically. The CHARMM27 force field was used [67,68]. The *apo*- and ligand-protein complexes were prepared for simulation by placing them within a triclinic box and surrounding them with the simple point charge water model to solvate the system. To balance the charges within the solvated setup, an equivalent quantity of Na^+^ and Cl^−^ counterions was introduced at a concentration of 150 mM. The server used the steepest descent integrator to minimize energy at a maximum of 5000 steps. The temperature and pressure were set at 300 K and 1.0 bar, respectively, for a period of 100 ps in the equilibration runs. Following the equilibration phase, the system underwent a production run of 50 ns using the leapfrog integrator. The SiBioLead simulation module generated the trajectory from the production runs and analyzed it automatically. RMSD and root mean square fluctuation (RMSF) were applied in the preliminary simulation calculations.

After the preliminary 50 ns simulations, we then performed a longer simulation (200 ns) on the National Supercomputing Center in Wuxi supercomputer “Sunway TaihuLight” (12-core Chinese-designed SW26010 manycore 64-bit RISC processors, Wuxi, China) for the herbal ligands with the greatest potential of synergistic mechanisms with ARB and could be detected via the metabolomics profiling in the herbal mixture. GROMACS 2018.2 was used [56,69,70] with the CHARMM27 force field [67,68]. Ligand topology for ARB and HQA004 was generated by SwissParam [71]. The ARB-herbal ligand-M^pro^ complex was solved in a dodecahedral box filled with TIP3P water, and a minimum of 2.0 nm distance was set between any M^pro^ atoms and the closest box edge [72]. Sodium and chloride ions were also added to the box to neutralize the charge and provide an approximate 150 mM salt concentration. A maximum of 500,000 steps was set to minimize energy via a steepest-descent gradient approach. Both isothermal-isochloric and isothermal-isobaric ensembles were employed to restrain each complex for 100 ps. Temperature and pressure in the system were maintained at 310 K and 1.0 bar, respectively, via a modified Berendsen thermostat [73] and a Parrinello-Rahman barostat [74]. The bond lengths were restricted via the LINCS algorithm [75]. Long-range electrostatic forces were calculated using the particle-mesh Ewald scheme with a 0.16 nm grid space [76], whereas a cutoff radius of 1.2 nm (Coulomb and van der Waals potentials) was used for short-range nonbonded interactions. A time-step of 2 fs was used; 200 ns production simulations were performed in triplicate, resulting in 600 ns per system, and thus a total of 2.4 µs worth of trajectories for the four arbidol-baicalein stoichiometries were studied. The Maxwell distribution was used for the generation of initial atomic velocities. Visual Molecular Dynamics 1.9.3 was applied to analyze and visualize the simulation trajectories [58,77].

Subsequently, the Molecular Mechanics Poisson-Boltzmann Surface Area (MM-PBSA) was calculated using the g_mmpbsa tool [78] (1 ns segments of the triplicate stabilized trajectories [79]) to quantify the free energy calculations [80]. Energy contributions from electrostatic, van der Waals, and polar solvation terms were predicted via the Adaptive Poisson-Boltzmann Solver [81]. Corresponding values for grid spacing (0.05 nm), solvent dielectric constant (80), and solute dielectric constant (2) were set on the system. The solvent-accessible surface area was employed to calculate the non-polar energy contribution, setting the probe radius to 0.14 nm. Nonetheless, the entropic energy terms were not considered for this study. We also analyzed RMSD, RMSF, radius of gyration, and inter-residue contact order via the suite of analysis tools provided by GROMACS.

We then performed wavelet analysis on the inter-subunit contact number time series to gain further insights into the effects of cooperative ligand binding on M^pro^ dynamics and intramolecular contacts [19]. By estimating the contributions of different periodic functions within the frequency range, this analysis allows for the determination of the periodic behavior of dynamics or residual contacts at each position [19]. Wavelet power spectra were computed utilizing R-4.2.1 [65] along with version 0.20.21 of the biwavelet library (available at https://github.com/tgouhier/biwavelet, accessed on 10 April 2025). The Morlet wavelet and its corresponding default parameters were employed in this process.

A PCA was performed to reduce the dimensionality of the data and to extract the high-amplitude, and most significant, concerted motions of M^pro^ complexes obtained from MD simulations [82,83]. For each M^pro^-ligand complex, PCA was performed on a single trajectory, which combines all three triplicate runs. The covar analysis module within GROMACS was used to calculate the mass-weighted covariance matrix of the backbone atoms of the M^pro^ dimer. The module anaeig was used to diagonalize the covariance matrix and extract the eigenvectors and eigenvalues, while the sham module was used to calculate the FEL over the first two principal components of each system, PC1 and PC2.

### 4.8. Luciferase Assays

To validate the data generated from our novel computational calculation, we used luciferase assays, which is a novel method to identify potential inhibitors of M^pro^ at the cellular level without a biosafety level 3 laboratory, to assess the synergistic effects of ARB and HQA004 [53]. HEK 293T cells (ATCC CRL-11268) were purchased from the American Type Culture Collection and cultured in Dulbecco’s Modified Eagle Medium (Gibco, Miami, FL, USA) supplemented with 10% fetal bovine serum, 100 U/mL of penicillin (Gibco, Miami, USA), and 100 μg/mL of streptomycin (Gibco, Miami, USA). We seeded 293T cells into solid black 96-well plates (10,000 cells in 0.1 mL medium per well). We then transfected the cells after 24 h with 40 ng SARS-CoV-2 M^pro^ plasmid. We utilized the 50% inhibitory concentration (IC_50_) values of each compound mentioned in its product descriptions as a reference to set up different concentrations of ARB (Lot Number: 142078, TargetMol Chemicals Inc. (Boston, MA, USA), IC_50_ value: 16 μM) and HQA004 (Lot Number: 163442, TargetMol Chemicals Inc. (Boston, USA), IC_50_ value: 3.12 μM), to investigate the potential synergistic ratios for the two compounds. The concentrations for ARB were 10, 20, 40, 50, 60, 80, and 100 times IC_50_ values and were equal to 160 μM, 320 μM, 640 μM, 800 μM, 960 μM, 1280 μM, and 1600 μM. In the meantime, 10, 20, 40, 60, 80, and 100 times IC_50_ values of HQA004 were set and equal to 31.2 μM, 62.4 μM, 124.8 μM, 187.2 μM, 249.6 μM, and 312 μM. Briefly, in Corning^®^ black 96-well plates (Corning, NY, USA), a total of 48 μL of liquid containing 5 μg of SARS-CoV-2 M^pro^ was added to the plates. Gradient dilutions of baicalein and abidol were made with Assay Buffer (PBS buffer containing 5% DMSO, pH = 7.2), and then they were mixed and incubated at room temperature for 1 h. A 2 μL fluorescent substrate containing 1 μM of Dabcyl-KTSAVLQSGFRKME-Edans (Genewiz, South Plainfield, NJ, USA) was added to the hole, and fluorescence determination was performed using BioTek Cytation 5 Cell Imaging Multimode Reader (Winooski, VT, USA) at 37 °C for 5 min away from light, with an excitation wavelength of 340 nm and emission wavelength of 490 nm. The experiment was repeated four times with three holes each time. The inhibition rate was calculated using the following formula: inhibition rate = (1 − data of the experimental group/data of the control group) × 100%. When data passed the normality of distribution within each group (verified by the Shapiro-Wilk test) and homogeneity of variances across groups (checked via the Brown-Forsythe test), we used the One-way ANOVA or unpaired Student’s *t*-test to evaluate the data. If data failed the normality test or showed heterogeneous variances, we applied the non-parametric Kruskal–Wallis test instead. All statistical analyses were performed using GraphPad Prism 9.0 (San Diego, CA, USA), and results were presented as mean ± standard deviation to ensure clarity and consistency. We considered the data statistically significant when the *p*-values were less than 0.05.

### 4.9. Synergistic Effects Evaluation

We used the Bliss synergy score and CI to evaluate synergism between ARB and HQA004, based on data from luciferase assays [84]. For the Bliss synergy score, we utilized the Bliss independence model in SynergyFinder 3.0 (https://synergyfinder.fimm.fi/; accessed on 22 February 2024), which is a publicly available online platform to calculate the expected drug combination responses [85]. The positive and negative deviations between the observed and expected responses indicate synergistic and antagonistic effects, respectively [86]. The platform also employs the cNMF algorithm to estimate outlier measurements [87]. When the score is equal to 0 means additive, less than 0 means antagonistic, or larger than 0 means synergistic. The most synergistic area was also provided by the platform. In terms of CI, we used CompuSyn 1.0, which was designed to quantify synergistic and antagonistic effects between drugs based on the Chou-Talalay theory, to calculate any value of the fraction affected (fa) [4]. When the CI is equal to 1 means additive, less than 1 means antagonists, or larger than 1 means synergism.

### 4.10. Surface Plasmon Resonance Approaches

We used the surface plasmon resonance approaches via a Biacore T200 to evaluate the binding affinity between M^pro^ and ABR or ARB+HQA004, to see whether any synergistic effects between ARB and HQA004 appeared [88]. We immobilized the prepared main protease to the surface of the CM5 chip as the stationary phase. We diluted the M^pro^ protein (20 μg/mL, containing immobilization buffer), and subsequently, we adhered to the manufacturer’s protocol to covalently couple it on channel 2 of the chip through an amine coupling kit. We utilized channel 1 as a blank control that did not contain cross-linked protein. For the ARB-only group, we diluted ARB with gradient concentrations (0.75 μΜ, 1.5 μM, 3 μM, 6 μM, 12 μM, 24 μM, 48 μM) to the running buffer (PBS, with 0.1% (*v*/*v*) DMSO, pH = 7.4) and then added to both channels 1 and 2 (30 μL/min flow rate). For the combination group, we set a fixed contraction of HQA004 to 20 μM, and the gradient concentrations of ARB were the same as those of the monotherapy group. We set up the contact time and dissociation time to 60 s and 300 s, respectively, with a 50% DMSO extra wash. To correct the effect caused by the solvent, we also employed an analytical buffer, which did not contain any drugs. We retrieved and analyzed all SPR data via Biacore T200 Evaluation Software (version 4.1; steady-state affinity analysis model).

## 5. Conclusions

In this study, we aimed to develop novel combination drugs by integrating TCM and Western antiviral agents against SARS-CoV-2, guided by clinical evidence and the theory of TCM-Western medicine integration. We utilized the SMMM approach and surface plasmon resonance approach to identify potential herbal components from a herbal remedy with synergistic effects to an anti-viral drug, ARB. This research revealed that our comprehensive strategy is suitable for new combination drug development. We conducted virtual screening and preliminary dynamics simulations and found that five compounds interacted with ARB in synergy. Only HQA004 could be identified in the herbal remedy by untargeted metabolomics, with ideal pharmacokinetic properties, and as a non-toxic compound. We extended the simulations to a longer period and found that the synergistic mechanism of HQA004 may be related to the *“Far” Addition Mechanism #2* with a 2:1 stoichiometric ratio of ARB to HQA004. We believe that the main reason synergism occurred is that the movements of ARB (diffusion and intramolecular conformational shifts) in the system were significantly reduced by HQA004. Moreover, this phenomenon is largely dependent on the binding position of HQA004. Additionally, we observed that only if the stoichiometric ratio of ABR and HQA004 is equal to 1:1, higher frequency structural oscillation occurs, which means that a higher number of molecules of ABR is recommended, compared to the number of HQA004, to generate a greater benefit. In vitro experiments also validated the findings of our computational calculations. Our work demonstrated that synergy exists and HQA004 acts as a supporter of ARB, co-partnering to inhibit M^pro^. For future work, we suggest testing the other four candidate compounds (HQA016, QHA013, JYH007, and JYH010) and conducting animal studies in a biosafety level 3 laboratory to explore therapeutic mechanisms and safety, highlighting that integrating TCM compounds with Western drugs via multi-ligand modeling offers a promising pathway for anti-coronaviral drug discovery.

## Figures and Tables

**Table 1:** Synergistic effects between arbidol and HQA004 (baicalein) based on fluorescence determination data.

Drug A	Drug B	Drug A Dosage (IC_50_)	Drug B Dosage (IC_50_)	Drug A Dosage (μM)	Drug B Dosage (μM)	Effects	Chou-Talalay CI	Bliss Synergy Score	Ratio (IC_50_)	Ratio (μM)		
Arbidol	Baicalein	10	10	160	31.2	0.072	0.60062	2.000854	1:1	200:39	≈	5:1
Arbidol	Baicalein	10	20	160	62.4	0.093	0.56821	1.810456	1:2	100:39	≈	3:1
Arbidol	Baicalein	10	40	160	124.8	0.114	0.72318	0.359348	1:4	50:39	≈	1:1
Arbidol	Baicalein	10	60	160	187.2	0.128	0.8929	−0.43471	1:6	100:117	≈	1:1
Arbidol	Baicalein	10	80	160	249.6	0.138	1.06439	−0.329	1:8	25:39	≈	1:1
Arbidol	Baicalein	10	100	160	312	0.153	1.16796	0.159993	1:10	20:39	≈	1:1
Arbidol	Baicalein	20	10	320	31.2	0.076	0.86929	1.928658	2:1	400:39	≈	10:1
Arbidol	Baicalein	20	20	320	62.4	0.101	0.67028	2.203209	1:1	200:39	≈	5:1
Arbidol	Baicalein	20	40	320	124.8	0.128	0.72071	1.388058	1:2	100:39	≈	3:1
Arbidol	Baicalein	20	60	320	187.2	0.139	0.88779	0.29493	1:3	200:117	≈	2:1
Arbidol	Baicalein	20	80	320	249.6	0.148	1.04871	0.322774	1:4	50:39	≈	1:1
Arbidol	Baicalein	20	100	320	312	0.174	1.05344	1.89968	1:5	40:39	≈	1:1
Arbidol	Baicalein	40	10	640	31.2	0.082	1.29173	0.608507	4:1	800:39	≈	21:1
Arbidol	Baicalein	40	20	640	62.4	0.113	0.81097	1.548198	2:1	400:39	≈	10:1
Arbidol	Baicalein	40	40	640	124.8	0.143	0.76168	1.113669	1:1	200:39	≈	5:1
Arbidol	Baicalein	40	60	640	187.2	0.157	0.87332	0.445113	2:3	400:117	≈	3:1
Arbidol	Baicalein	40	80	640	249.6	0.165	1.01788	0.265352	1:2	100:39	≈	3:1
Arbidol	Baicalein	40	100	640	312	0.19	1.01695	1.862945	2:5	80:39	≈	2:1
Arbidol	Baicalein	50	10	800	31.2	0.098	1.06719	0.773526	5:1	1000:39	≈	26:1
Arbidol	Baicalein	50	20	800	62.4	0.126	0.75942	1.438732	5:2	500:39	≈	13:1
Arbidol	Baicalein	50	40	800	124.8	0.148	0.7872	0.284372	5:4	250:39	≈	6:1
Arbidol	Baicalein	50	60	800	187.2	0.163	0.87914	−0.34147	5:6	500:117	≈	4:1
Arbidol	Baicalein	50	80	800	249.6	0.174	0.99087	−0.12508	5:8	125:39	≈	3:1
Arbidol	Baicalein	50	100	800	312	0.203	0.9618	1.859403	1:2	100:39	≈	3:1
Arbidol	Baicalein	60	10	960	31.2	0.101	1.16692	0.691017	6:1	400:13	≈	31:1
Arbidol	Baicalein	60	20	960	62.4	0.139	0.7056	2.2956	3:1	200:13	≈	15:1
Arbidol	Baicalein	60	40	960	124.8	0.166	0.70144	1.612178	3:2	100:13	≈	8:1
Arbidol	Baicalein	60	60	960	187.2	0.174	0.84117	0.388165	1:1	200:39	≈	5:1
Arbidol	Baicalein	60	80	960	249.6	0.191	0.90367	1.217708	3:4	50:13	≈	4:1
Arbidol	Baicalein	60	100	960	312	0.214	0.92087	2.598663	3:5	40:13	≈	3:1
Arbidol	Baicalein	80	10	1280	31.2	0.126	0.91745	0.917917	8:1	1600:39	≈	41:1
Arbidol	Baicalein	80	20	1280	62.4	0.146	0.77646	0.814347	4:1	800:39	≈	21:1
Arbidol	Baicalein	80	40	1280	124.8	0.171	0.75828	−0.01067	2:1	400:39	≈	10:1
Arbidol	Baicalein	80	60	1280	187.2	0.181	0.87029	−0.96663	4:3	800:117	≈	7:1
Arbidol	Baicalein	80	80	1280	249.6	0.202	0.89239	0.277942	1:1	200:39	≈	5:1
Arbidol	Baicalein	80	100	1280	312	0.233	0.85803	2.457203	4:5	160:39	≈	4:1
Arbidol	Baicalein	100	10	1600	31.2	0.163	0.61863	2.07305	10:1	2000:39	≈	51:1
Arbidol	Baicalein	100	20	1600	62.4	0.177	0.60655	1.415308	5:1	1000:39	≈	26:1
Arbidol	Baicalein	100	40	1600	124.8	0.193	0.67776	−0.23855	5:2	500:39	≈	13:1
Arbidol	Baicalein	100	60	1600	187.2	0.199	0.80732	−1.58835	5:3	1000:117	≈	9:1
Arbidol	Baicalein	100	80	1600	249.6	0.214	0.86781	−0.93566	5:4	250:39	≈	6:1
Arbidol	Baicalein	100	100	1600	312	0.251	0.80203	1.891295	1:1	200:39	≈	5:1

Note: CI: Combination index. Effects represent the inhibition rate. For the Chou-Talalay combination index, <1 represents synergistic effects, =1 represents additive effects, and >1 represents antagonism. For the Bliss synergy score, >0 represents synergism, =0 represents additive effects, and <0 represents antagonism.

## Data Availability

The original contributions presented in this study are included in the article/Supplementary Material. Further inquiries can be directed to the corresponding authors.

## Supplementary Materials

The following supporting information can be downloaded at https://www.mdpi.com/article/10.3390/ph18071054/s1.

## Abbreviations

The following abbreviations are used in this manuscript:

ADME/T	Absorption, distribution, metabolism, excretion, and toxicity
ARB	Arbidol
COVID-19	Coronavirus disease
CI	Combination index
fa	Fraction affected
ΔΔGsynstab	Synergistic stabilization energy
H-bonds	Hydrogen bonds
MD	Molecular dynamics
MM-PBSA	Molecular Mechanics Poisson-Boltzmann Surface Area
Mpro	Main protease
PAINS	Pan-Assay Interference Compounds
RMSD	Root mean square deviation of backbone Cɑ atoms
RMSF	Root mean square fluctuation
SARS-CoV-2	Severe acute respiratory syndrome coronavirus 2
SMMM	Structure-based multi-ligand molecular modeling
TCM	Traditional Chinese medicine
TCMSP	Traditional Chinese Medicine Systems Pharmacology Database and Analysis Platform
TQG	Toujie Quwen Granules
