# Supplementary material for: Supercomputing Multi-Ligand Modeling, Simulation, Wavelet Analysis and Surface Plasmon Resonance to Develop Novel Combination Drugs: A Case Study of Arbidol and Baicalein Against Main Protease of SARS-CoV-2
Source: Pharmaceuticals (Basel). 2025 Jul 17;18(7):1054. doi: 10.3390/ph18071054 (PMC12300969; doi:10.3390/ph18071054)
Supplement: Supplementary file 1 [file pharmaceuticals-18-01054-s001.zip › Li et al_Supplementary Materials_17Jul2025.pdf]

Supplementary Materials for

Supercomputing multi-ligand modeling, simulation, wavelet analysis and surface plasmon resonance to develop novel combination drugs: a case study of arbidol and baicalein against main protease of SARS-CoV-2

Hong Li [1,2,3,†], Hailong Su [4,5,†], Akari Komori [3], Shuxuan Yang [2], Hailang Luo [2], Angela Wei Hong Yang [6], Xiaomin Sun [2], Hongwei Li [5,], Andrew Hung [3,*] and Xiaoshan Zhao [1,2,*]*

Correspondence to: Prof Hongwei Li lihw@smu.edu.cn;
Dr Andrew Hung andrew.hung@rmit.edu.au;
Prof Xiaoshan Zhao zhaoxs@smu.edu.cn

Contents

Figure S1 Sample of Toujie Quwen Granules for metabolomics profiling. 2
Figure S2 Positive mode of metabolomics profiling of Tougjie Quwen Granules......................... 3
Table S1. Detailed information on the ingredients of Toujie Quwen Granules and numbers of compounds identified from the TCMSP database. 4
Table S2. Characteristics of the 238 compounds identified from Toujie Quwen Granules. 5
Table S3. Simplified Molecular-input Line-entry System formats of 238 compounds identified from Toujie Quwen Granules. 16
Table S4. Details of the normal distribution test of the docking results (kcal/mol). 27
Table S5. Raw data of multiple ligand molecular docking results (kcal/mol)............................. 28
Table S6. Raw data of Luciferase assays... 30
Data S1. The grid box parameters for blind docking.. 32
Data S2. Data of metabolomics profiling of Toujie Quwen Granules (positive and negetive modes)... 32

Figure S1 Sample of Toujie Quwen Granules for metabolomics profiling.

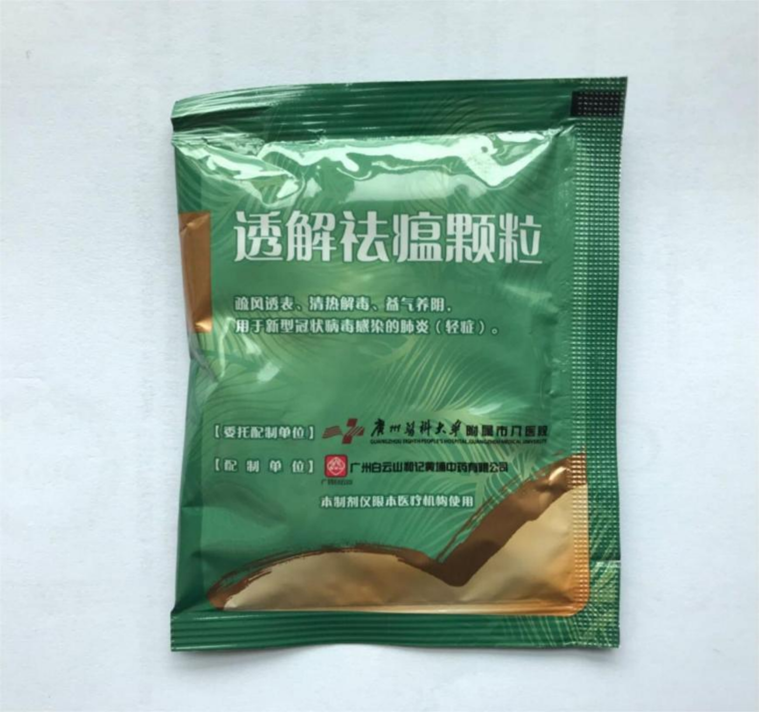

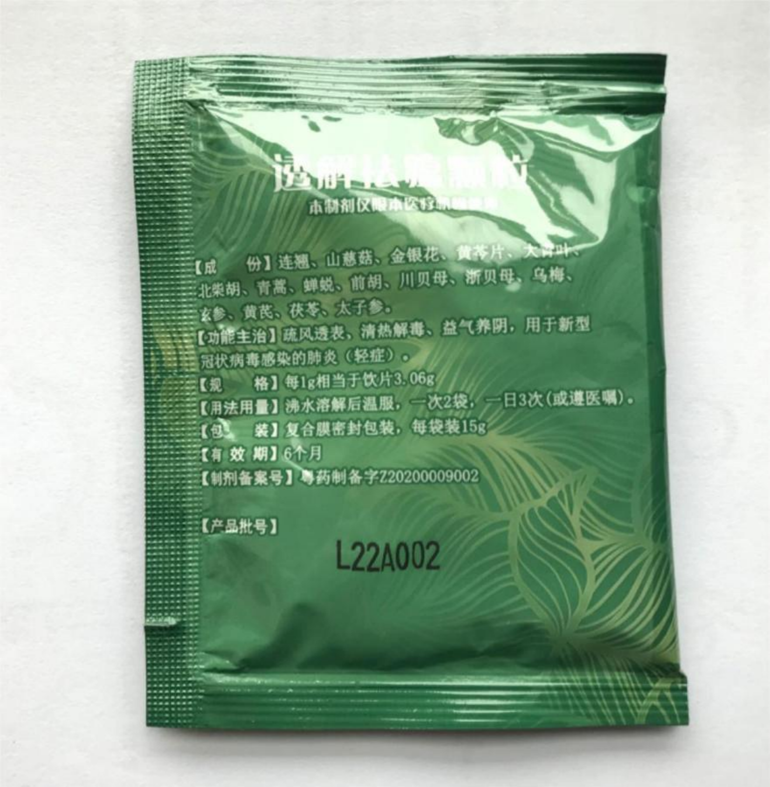

Figure S2 Positive mode of metabolomics profiling of Tougjie Quwen Granules

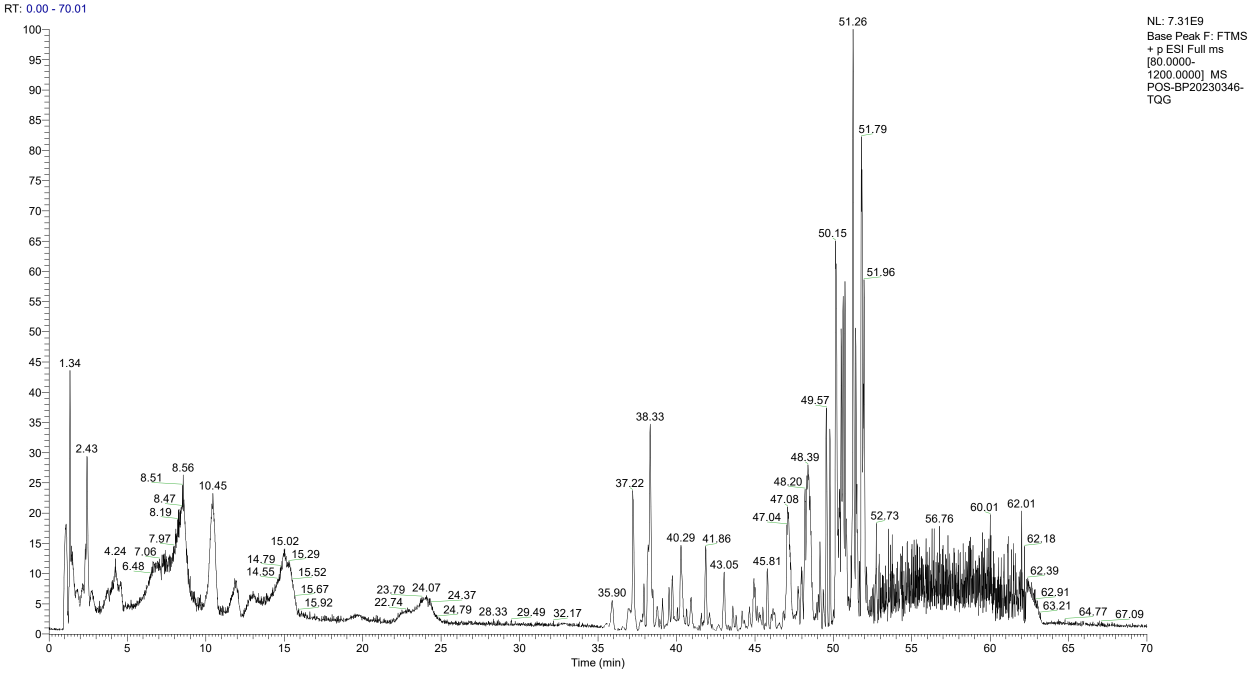

Table S1. Detailed information on the ingredients of Toujie Quwen Granules and numbers of compounds identified from the TCMSP database.

Chinese *Pin Yin* names	Abbreviation	Pharmaceutical names	Chinese names	Dosages (g)	Numbers of search results	Numbers of included compounds
Lian qiao	LQA	Forsythiae Fructus	连翘	30	150	23
Shan ci gu	SCG	Pseudobulbus Cremastrae Seu Pleiones	山慈菇	20	18	3
Jin yin hua	JYH	Lonicerae Japonicae Flos	金银花	15	236	23
Huang qin	HQA	Scutellariae Radix	黄芩	10	143	36
Da qing ye	DQY	Isatidis Folium	大青叶	10	45	10
Chai hu	CHA	Bupleuri Radix	柴胡	5	349	17
Qing hao	QHA	Artemisiae Annuae Herba	青蒿	10	126	22
Chan tui	N/A	Cicadae Periostracum	蝉蜕	10	8	0
Qian hu	QHB	Peucedani Radix	前胡	5	101	24
Chuan bei mu	CBM	Fritillariae Cirrhosae Bulbus	川贝	10	63	13
Zhe bei mu	ZBM	Fritillariae Thunbergii Bulbus	浙贝	10	17	7
Wu mei	WMA	Mume Fructus	乌梅	30	40	8
Xuan shen	XSA	Scrophulariae Radix	玄参	10	47	9
Huang qi	HQB	Scutellariae Radix	黄芪	45	87	20
Fu ling	FLA	Poria	茯苓	30	34	15
Tai zi shen	TZS	Pseudostellariae Radix	太子参	15	25	8

Note: Abbr.: Abbreviations. Data was retrieved from the Traditional Chinese Medicine Systems Pharmacology Database and Analysis Platform (TCMSP; https://tcmsp-e.com/).

Table S2. Characteristics of the 238 compounds identified from Toujie Quwen Granules.

Compound code	Molecular name	Mol ID	PubChem CID/SID	MW	AlogP	Hdon	Hacc	OB (%)	Caco-2	BBB	DL	FASA-	HL
**1. Lian qiao (Forsythiae Fructus; n = 23)**													
LQA001	wogonin	MOL000173	5281703	284.28	2.59	2	5	30.68	0.79	0.04	0.23	0.32	17.75
LQA002	20(S)-dammar-24-ene-3β,20-diol-3-acetate	MOL003281	N/A	486.86	7.34	1	3	40.23	0.93	0.28	0.82	0.23	9.14
LQA003	(2R,3R,4S)-4-(4-hydroxy-3-methoxy-phenyl)-7-methoxy-2,3-dimethylol-tetralin-6-ol	MOL003283	160521	360.44	2.25	4	6	66.51	-0.2	-1.17	0.39	0.23	1.26
LQA004	(3R,4R)-3,4-bis[(3,4-dimethoxyphenyl)methyl]oxolan-2-one	MOL003290	384877	386.48	3.97	0	6	52.3	0.78	0.17	0.48	0.23	3.07
LQA005	(+)-pinoresinol monomethyl ether	MOL003295	N/A	372.45	2.38	1	6	53.08	0.69	0	0.57	0	3.02
LQA006	PHILLYRIN	MOL003305	101712	534.61	0.48	4	11	36.4	-0.79	-1.25	0.86	0.2	3.12
LQA007	ACon1_001697	MOL003306	21722915	372.45	2.38	1	6	85.12	0.76	0	0.57	0	2.12
LQA008	(+)-pinoresinol monomethyl ether-4-D-beta-glucoside_qt	MOL003308	N/A	372.45	2.38	1	6	61.2	0.7	0.12	0.57	0	2.9
LQA009	3beta-Acetyl-20,25-epoxydammarane-24alpha-ol	MOL003315	80510599	502.86	6.04	1	4	33.07	0.75	0.24	0.79	0.22	7.82
LQA010	Mairin	MOL000211	64971	456.78	6.52	2	3	55.38	0.73	0.22	0.78	0.26	8.87
LQA011	FORSYTHINOL	MOL003322	N/A	372.45	2.38	1	6	81.25	0.59	-0.08	0.57	0.23	2.72
LQA012	(-)-Phillygenin	MOL003330	3083590	372.45	2.38	1	6	95.04	0.75	0.07	0.57	0.2	1.97
LQA013	β-amyrin acetate	MOL003344	92156	468.84	7.68	0	2	42.06	1.36	1.1	0.74	0	1.98
LQA014	hyperforin	MOL003347	441298	536.87	8.62	1	4	44.03	0.87	0.4	0.6	0	2.15
LQA015	adhyperforin	MOL003348	9963735	550.9	9.07	1	4	44.03	0.93	0.58	0.61	0	0.84
LQA016	Lactucasterol	MOL003365	50226759	426.75	6.72	1	2	40.99	0.88	0.5	0.85	0.26	5.53
LQA017	Onjixanthone I	MOL003370	5320290	302.3	2.65	1	6	79.16	0.84	0.04	0.3	0.2	14.86
LQA018	beta-sitosterol	MOL000358	222284	414.79	8.08	1	1	36.91	1.32	0.99	0.75	0.23	5.36
LQA019	kaempferol	MOL000422	5280863	286.25	1.77	4	6	41.88	0.26	-0.55	0.24	0	14.74
LQA020	arctiin	MOL000522	100528	534.61	1.81	4	11	34.45	-0.73	-1.5	0.84	0.24	1.41
LQA021	luteolin	MOL000006	5280445	286.25	2.07	4	6	36.16	0.19	-0.84	0.25	0.39	15.94
LQA022	bicuculline	MOL000791	10237	367.38	2.83	0	7	69.67	0.72	0.02	0.88	0.33	15.83

Compound code	Molecular name	Mol ID	PubChem CID/SID	MW	AlogP	Hdon	Hacc	OB (%)	Caco-2	BBB	DL	FASA-	HL
LQA023	quercetin	MOL000098	5280343	302.25	1.5	5	7	46.43	0.05	-0.77	0.28	0.38	14.4
**2. Shan ci gu (Pseudobulbus Cremastrae Seu Pleiones; n = 3)**													
SCG001	beta-sitosterol	MOL000358	222284	414.79	8.08	1	1	36.91	1.32	0.99	0.75	0.23	5.36
SCG002	Stigmasterol	MOL000449	5280794	412.77	7.64	1	1	43.83	1.44	1	0.76	0.22	5.57
SCG003	2-methoxy-9,10-dihydrophenanthrene-4,5-diol	MOL007991	11506999	242.29	3.4	2	3	44.97	1.1	0.61	0.18	0.31	-0.81
**3. Jin yin hua (Lonicerae Japonicae Flos; n = 23)**													
JYH001	Mandenol	MOL001494	5282184	308.56	6.99	0	2	42	1.46	1.14	0.19	0.25	5.39
JYH002	Ethyl linolenate	MOL001495	6371716	306.54	6.55	0	2	46.1	1.54	1.12	0.2	0.25	6.2
JYH003	phytofluene	MOL002707	6436722	543.02	14.1	0	0	43.18	2.29	1.76	0.5	0.3	2.6
JYH004	Eriodyctiol (flavanone)	MOL002914	373261	288.27	2.03	4	6	41.35	0.05	-0.66	0.24	0.39	15.88
JYH005	(-)-(3R,8S,9R,9aS,10aS)-9-ethenyl-8-(beta-D-glucopyranosyloxy)-2,3,9,9a,10,10a-hexahydro-5-oxo-5H,8H-pyrano[4,3-d]oxazolo[3,2-a]pyridine-3-carboxylic acid_qt	MOL003006	N/A	281.29	-0.96	2	7	87.47	-0.55	-0.89	0.23	0	5.5
JYH006	secologanic dibutylacetal_qt	MOL003014	N/A	384.57	3.58	1	6	53.65	0.34	-0.26	0.29	0	2.79
JYH007	beta-carotene	MOL002773	5280489	536.96	12	0	0	37.18	2.25	1.52	0.58	0.33	4.36
JYH008	ZINC03978781	MOL003036	11870462	412.77	7.64	1	1	43.83	1.32	0.96	0.76	0	5.79
JYH009	Chryseriol	MOL003044	5280666	300.28	2.32	3	6	35.85	0.39	-0.53	0.27	0.32	16.31
JYH010	kryptoxanthin	MOL003059	1.62E+08	552.96	10.76	1	1	47.25	1.69	0.05	0.57	0.33	4.37
JYH011	4,5'-Retro-.beta.,.beta.-Carotene-3,3'-dione, 4',5'-didehydro-	MOL003062	5380108	562.9	9.27	0	2	31.22	1.17	-0.69	0.55	0.39	5.39
JYH012	5-hydroxy-7-methoxy-2-(3,4,5-trimethoxyphenyl)chromone	MOL003095	10970376	358.37	2.8	1	7	51.96	0.88	-0.21	0.41	0.17	15.98
JYH013	7-epi-Vogeloside	MOL003101	N/A	432.47	-0.19	4	11	46.13	-1.3	-1.73	0.58	0.23	2.77
JYH014	Caeruloside C	MOL003108	N/A	550.57	-3.7	7	15	55.64	-2.91	-3.6	0.73	0.28	2.85
JYH015	Centauroside_qt	MOL003111	N/A	434.48	0.38	2	9	55.79	-0.84	-1.51	0.5	0	5.18
JYH016	Ioniceracetalides B_qt	MOL003117	N/A	314.37	1	1	7	61.19	-0.09	-0.37	0.19	0	2.85
JYH017	XYLOSTOSIDINE	MOL003124	14466553	415.51	-1.66	4	9	43.17	-1.07	-1.32	0.64	0.27	9.15
JYH018	dinethylsecologanoside	MOL003128	N/A	434.44	-0.79	4	12	48.46	-1.37	-1.69	0.48	0.24	3.18
JYH019	beta-sitosterol	MOL000358	222284	414.79	8.08	1	1	36.91	1.32	0.99	0.75	0.23	5.36

Compound code	Molecular name	Mol ID	PubChem CID/SID	MW	AlogP	Hdon	Hacc	OB (%)	Caco-2	BBB	DL	FASA-	HL
JYH020	kaempferol	MOL000422	5280863	286.25	1.77	4	6	41.88	0.26	-0.55	0.24	0	14.74
JYH021	Stigmasterol	MOL000449	5280794	412.77	7.64	1	1	43.83	1.44	1	0.76	0.22	5.57
JYH022	luteolin	MOL000006	5280445	286.25	2.07	4	6	36.16	0.19	-0.84	0.25	0.39	15.94
JYH023	quercetin	MOL000098	5280343	302.25	1.5	5	7	46.43	0.05	-0.77	0.28	0.38	14.4
**4. Huang qin (Scutellariae Radix; n = 36)**													
HQA001	acacetin	MOL001689	5280442	284.28	2.59	2	5	34.97	0.67	-0.05	0.24	0.35	17.25
HQA002	wogonin	MOL000173	5281703	284.28	2.59	2	5	30.68	0.79	0.04	0.23	0.32	17.75
HQA003	(2R)-7-hydroxy-5-methoxy-2-phenylchroman-4-one	MOL000228	821279	270.3	2.82	1	4	55.23	0.87	0.26	0.2	0.34	17.02
HQA004	baicalein	MOL002714	5281605	270.25	2.33	3	5	33.52	0.63	-0.05	0.21	0.36	16.25
HQA005	5,8,2'-Trihydroxy-7-methoxyflavone	MOL002908	156992	300.28	2.32	3	6	37.01	0.76	-0.07	0.27	0.28	16.17
HQA006	5,7,2,5-tetrahydroxy-8,6-dimethoxyflavone	MOL002909	44258628	376.34	2.02	4	9	33.82	0.35	-0.59	0.45	0.19	15.94
HQA007	Carthamidin	MOL002910	188308	288.27	2.03	4	6	41.15	0.16	-0.42	0.24	0.36	15.81
HQA008	2,6,2',4'-tetrahydroxy-6'-methoxychaleone	MOL002911	N/A	302.3	2.62	4	6	69.04	-0.07	-0.32	0.22	0.34	21.89
HQA009	Dihydrobaicalin_qt	MOL002913	14135323	272.27	2.3	3	5	40.04	0.56	0.18	0.21	0.36	16.13
HQA010	Eriodyctiol (flavanone)	MOL002914	373261	288.27	2.03	4	6	41.35	0.05	-0.66	0.24	0.39	15.88
HQA011	Salvigenin	MOL002915	161271	328.34	2.82	1	6	49.07	0.86	-0.03	0.33	0.21	15.87
HQA012	5,2',6'-Trihydroxy-7,8-dimethoxyflavone	MOL002917	5322059	330.31	2.3	3	7	45.05	0.48	-0.11	0.33	0.25	16.37
HQA013	5,7,2',6'-Tetrahydroxyflavone	MOL002925	5321865	286.25	2.07	4	6	37.01	0.18	-0.56	0.24	0.38	18
HQA014	dihydrooroxylin A	MOL002926	N/A	286.3	2.55	2	5	38.72	0.71	0.03	0.23	0.29	17.58
HQA015	Skullcapflavone II	MOL002927	124211	374.37	2.54	2	8	69.51	0.68	-0.07	0.44	0.2	16.14
HQA016	oroxylin a	MOL002928	5320315	284.28	2.59	2	5	41.37	0.76	0.13	0.23	0.29	17.15
HQA017	Panicolin	MOL002932	5320399	314.31	2.57	2	6	76.26	0.84	0.31	0.29	0.24	16.78
HQA018	5,7,4'-Trihydroxy-8-methoxyflavone	MOL002933	5322078	300.28	2.32	3	6	36.56	0.46	-0.4	0.27	0.31	16.93
HQA019	NEOBAICALEIN	MOL002934	N/A	374.37	2.54	2	8	104.34	0.74	-0.19	0.44	0.18	16.5
HQA020	DIHYDROOROXYLIN	MOL002937	25721350	286.3	2.55	2	5	66.06	0.67	0.13	0.23	0.31	17.17
HQA021	beta-sitosterol	MOL000358	222284	414.79	8.08	1	1	36.91	1.32	0.99	0.75	0.23	5.36
HQA022	sitosterol	MOL000359	12303645	414.79	8.08	1	1	36.91	1.32	0.87	0.75	0.22	5.37
HQA023	Norwogonin	MOL000525	5281674	270.25	2.33	3	5	39.4	0.6	-0.17	0.21	0.39	16.93

Compound code	Molecular name	Mol ID	PubChem CID/SID	MW	AlogP	Hdon	Hacc	OB (%)	Caco-2	BBB	DL	FASA-	HL
HQA024	5,2'-Dihydroxy-6,7,8-trimethoxyflavone	MOL000552	159029	344.34	2.55	2	7	31.71	0.93	0	0.35	0.22	16.47
HQA025	ent-Epicatechin	MOL000073	182232	290.29	1.92	5	6	48.96	0.02	-0.64	0.24	0.34	0.63
HQA026	Stigmasterol	MOL000449	5280794	412.77	7.64	1	1	43.83	1.44	1	0.76	0.22	5.57
HQA027	coptisine	MOL001458	72322	320.34	3.25	0	4	30.67	1.21	0.32	0.86	0.26	9.33
HQA028	bis[(2S)-2-ethylhexyl] benzene-1,2-dicarboxylate	MOL001490	7057920	390.62	7.57	0	4	43.59	0.98	0.68	0.35	0.28	3.02
HQA029	Supraene	MOL001506	638072	410.8	11.33	0	0	33.55	2.08	1.73	0.42	0.27	2.72
HQA030	Diop	MOL002879	33934	390.62	7.44	0	4	43.59	0.79	0.26	0.39	0.28	3.6
HQA031	epiberberine	MOL002897	160876	336.39	3.45	0	4	43.09	1.17	0.4	0.78	0.19	6.1
HQA032	Moslosooflavone	MOL008206	188316	298.31	2.84	1	5	44.09	1.01	0.54	0.25	0.26	17.02
HQA033	11,13-Eicosadienoic acid, methyl ester	MOL010415	5365674	322.59	7.55	0	2	39.28	1.46	1.24	0.23	0.21	5.44
HQA034	5,7,4'-trihydroxy-6-methoxyflavanone	MOL012245	26213330	302.3	2.28	3	6	36.63	0.43	-0.32	0.27	0.28	16.12
HQA035	5,7,4'-trihydroxy-8-methoxyflavanone	MOL012246	42608119	302.3	2.28	3	6	74.24	0.37	-0.43	0.26	0.31	16.85
HQA036	rivularin	MOL012266	13889022	344.34	2.55	2	7	37.94	0.65	-0.13	0.37	0.21	16.25
**5. Da qing ye (Isatidis Folium; n = 10)**													
DQY001	poriferast-5-en-3beta-ol	MOL001771	457801	414.79	8.08	1	1	36.91	1.45	1.14	0.75	0	5.07
DQY002	Indigo	MOL001781	5318432	262.28	2.11	2	4	38.2	0.83	0.02	0.26	0.44	77.01
DQY003	6-(3-oxoindolin-2-ylidene)indolo[2,1-b]quinazolin-12-one	MOL001810	3035728	363.39	3.54	1	5	45.28	1.19	0.48	0.89	0.44	47.91
DQY004	Indicaxanthin	MOL002308	6096870	308.32	-2.26	3	7	31.79	-0.92	-0.96	0.22	0.06	5.69
DQY005	indirubin	MOL002309	5359405	262.28	1.88	2	4	48.59	1.06	0.44	0.26	0.44	44.44
DQY006	Glycyrol	MOL002311	5320083	366.39	4.85	2	6	90.78	0.71	-0.2	0.67	0.28	9.85
DQY007	C05837	MOL002318	6602378	448.52	-0.28	6	10	66.02	-1.74	-2.39	0.48	0.24	2.47
DQY008	γ-sitosterol	MOL002320	457801	414.79	8.08	1	1	36.91	1.33	0.88	0.75	0.22	5.05
DQY009	isovitexin	MOL002322	162350	432.41	-0.06	7	10	31.29	-1.24	-2.01	0.72	0.33	16.45
DQY010	beta-sitosterol	MOL000358	222284	414.79	8.08	1	1	36.91	1.32	0.99	0.75	0.23	5.36
**6. Chai hu (Bupleuri Radix; n = 17)**													
CHA001	Linoleyl acetate	MOL001645	5319042	308.56	6.85	0	2	42.1	1.36	1.08	0.2	0.21	7.48
CHA002	Baicalin	MOL002776	64982	446.39	0.64	6	11	40.12	-0.85	-1.74	0.75	0.36	17.36
CHA003	Stigmasterol	MOL000449	5280794	412.77	7.64	1	1	43.83	1.44	1	0.76	0.22	5.57
CHA004	isorhamnetin	MOL000354	5281654	316.28	1.76	4	7	49.6	0.31	-0.54	0.31	0.32	14.34

Compound code	Molecular name	Mol ID	PubChem CID/SID	MW	AlogP	Hdon	Hacc	OB (%)	Caco-2	BBB	DL	FASA-	HL
CHA005	kaempferol	MOL000422	5280863	286.25	1.77	4	6	41.88	0.26	-0.55	0.24	0	14.74
CHA006	3,5,6,7-tetramethoxy-2-(3,4,5-trimethoxyphenyl)chromone	MOL004598	389001	432.46	2.54	0	9	31.97	0.75	0.08	0.59	0.13	15.54
CHA007	Areapillin	MOL004609	158311	360.34	2.29	3	8	48.96	0.6	-0.29	0.41	0.16	16.52
CHA008	Cubebin	MOL013187	117443	356.4	3.19	1	6	57.13	0.47	-0.41	0.64	0.31	12.4
CHA009	Longikaurin A	MOL004624	433636	348.48	1.16	3	5	47.72	0.08	0.09	0.53	0.27	1.71
CHA010	Octalupine	MOL004628	1.04E+08	264.41	-0.07	1	4	47.82	0.48	0.3	0.28	0.17	4.17
CHA011	Sainfuran	MOL004644	185034	286.3	3.38	2	5	79.91	0.9	0.23	0.23	0.22	8.58
CHA012	Troxerutin	MOL004648	N/A	346.56	5.89	3	3	31.6	0.35	-0.38	0.28	0.3	4.36
CHA013	(+)-Anomalin	MOL004653	5319252	426.5	5.05	0	7	46.06	0.46	0	0.66	0.36	1.03
CHA014	saikosaponin c_qt	MOL004702	N/A	472.78	3.71	3	4	30.5	0.03	-0.85	0.63	0.2	6.12
CHA015	α-spinasterol	MOL004718	5281331	412.77	7.64	1	1	42.98	1.28	0.79	0.76	0.22	6.46
CHA016	petunidin	MOL000490	441774	317.29	1.65	5	7	30.05	0.16	-0.64	0.31	0	1.21
CHA017	quercetin	MOL000098	5280343	302.25	1.5	5	7	46.43	0.05	-0.77	0.28	0.38	14.4
**7. Qing hao (Artemisiae Annuae Herba; n = 22)**													
QHA001	EUPATIN	MOL002235	5317287	360.34	1.99	3	8	50.8	0.53	-0.26	0.41	0.22	13.94
QHA002	isorhamnetin	MOL000354	5281654	316.28	1.76	4	7	49.6	0.31	-0.54	0.31	0.32	14.34
QHA003	sitosterol	MOL000359	12303645	414.79	8.08	1	1	36.91	1.32	0.87	0.75	0.22	5.37
QHA004	Tamarixetin	MOL004083	5281699	316.28	1.76	4	7	32.86	0.11	-0.44	0.31	0	14.59
QHA005	Patuletin	MOL004112	5281678	332.28	1.49	5	8	53.11	0.01	-0.71	0.34	0	14.31
QHA006	kaempferol	MOL000422	5280863	286.25	1.77	4	6	41.88	0.26	-0.55	0.24	0	14.74
QHA007	Stigmasterol	MOL000449	5280794	412.77	7.64	1	1	43.83	1.44	1	0.76	0.22	5.57
QHA008	Areapillin	MOL004609	158311	360.34	2.29	3	8	48.96	0.6	-0.29	0.41	0.16	16.52
QHA009	Artemetin	MOL005229	5320351	388.4	2.31	1	8	49.55	0.81	-0.09	0.48	0.15	15.01
QHA010	luteolin	MOL000006	5280445	286.25	2.07	4	6	36.16	0.19	-0.84	0.25	0.39	15.94
QHA011	Skrofulein	MOL007274	188323	314.31	2.57	2	6	30.35	0.72	-0.34	0.3	0.26	15.8
QHA012	artemisitene	MOL007389	11000442	280.35	3.15	0	5	54.36	0.54	0.59	0.31	0.33	-0.75
QHA013	vicenin-2_qt	MOL007400	N/A	270.25	2.82	3	5	45.84	0.25	-0.17	0.21	0.03	16.75
QHA014	Cirsiliol	MOL007401	160237	330.31	2.3	3	7	43.46	0.55	-0.51	0.34	0.25	15.04
QHA015	vitexin_qt	MOL007404	N/A	270.25	1.17	3	5	52.18	0.29	-0.4	0.21	0.03	3.4
QHA016	DMQT	MOL007412	5281603	346.31	1.55	4	8	42.6	0.31	-0.69	0.37	0.25	15.16

Compound code	Molecular name	Mol ID	PubChem CID/SID	MW	AlogP	Hdon	Hacc	OB (%)	Caco-2	BBB	DL	FASA-	HL
QHA017	[(2S)-2-[[(2S)-2-(benzoylamino)-3-phenylpropanoyl]amino]-3-phenylpropyl] acetate	MOL007415	10026486	444.57	4.02	2	6	58.02	0.32	-0.26	0.52	0.41	6.03
QHA018	6,8-di-c-glucosylapigenin_qt	MOL007423	N/A	270.25	1.17	3	5	59.85	0.38	-0.49	0.21	0.04	3.39
QHA019	artemisinin	MOL007424	68827	282.37	3.14	0	5	49.88	0.5	0.49	0.31	0.3	-0.59
QHA020	dihydroartemisinin	MOL007425	3000518	284.39	2.76	1	5	50.75	0.4	0.27	0.3	0.23	-0.4
QHA021	deoxyartemisinin	MOL007426	12814879	266.37	2.15	0	4	54.47	0.66	0.69	0.26	0.29	4.66
QHA022	quercetin	MOL000098	5280343	302.25	1.5	5	7	46.43	0.05	-0.77	0.28	0.38	14.4
**8. Qian hu (Peucedani Radix; n = 24)**													
QHB001	(8S,9R)-9-hydroxy-8-(2-hydroxypropan-2-yl)-8,9-dihydrofuro[2,3-h]chromen-2-one	MOL013076	11817856	262.28	1.2	2	5	37.3	0.1	-0.19	0.2	0.35	3
QHB002	Decursin	MOL013077	442126	328.39	3.96	0	5	39.27	0.77	0.25	0.38	0.29	-1.77
QHB003	praeruptorin E	MOL013078	6440581	428.52	4.88	0	7	51.22	0.37	-0.05	0.66	0.35	5.37
QHB004	dl-praeruptorin a	MOL013079	38347601	386.43	3.5	0	7	46.46	0.52	0.09	0.53	0.31	4.5
QHB005	Decussine	MOL013081	156336	301.42	3.43	0	2	39.83	1.74	1.12	0.65	0.2	9.29
QHB006	Skimmin (8CI)	MOL013083	99693	324.31	-0.27	4	8	38.35	-1.02	-1.57	0.32	0.3	3.57
QHB007	Peucedanocoumarin II	MOL013087	5320509	386.43	3.5	0	7	63.48	0.25	-0.26	0.53	0.38	3.17
QHB008	rubricauloside	MOL013093	196862	602.65	-1.5	7	15	58.36	-1.94	-2.52	0.71	0.24	1.28
QHB009	8-[(2R)-2,3-dihydroxy-3-methyl-butyl]-5,7-dimethoxy-coumarin	MOL013094	176970	308.36	1.66	2	6	48.57	-0.03	-0.5	0.21	0.24	2.05
QHB010	Sporidesmin	MOL013095	99596	474	1.35	2	9	58.31	-0.08	-0.54	0.76	0.38	15.31
QHB011	[(9R)-8,8-dimethyl-2-oxo-9,10-dihydropyrano[6,5-h]chromen-9-yl] (Z)-2-methylbut-2-enoate	MOL013098	668081	328.39	3.96	0	5	87.48	0.61	0.3	0.37	0.36	1.98
QHB012	(2S)-2-(1-hydroxy-1-methyl-ethyl)-9-[(2S,3R,4R,5S,6R)-3,4,5-trihydroxy-6-methylol-tetrahydropyran-2-yl]oxy-2,3-dihydrofuro[3,2-g]chromen-7-one	MOL013100	21733113	424.44	-0.14	5	10	45.33	-1.31	-1.65	0.73	0.27	5.23
QHB013	rutarin_qt	MOL013101	44146779	262.28	1.76	2	5	70.1	0.2	-0.28	0.2	0.32	3.11
QHB014	532-16-1	MOL013103	1781413	366.54	7	0	3	46.57	1.43	0.85	0.44	0.31	3.69
QHB015	Ammidin	MOL001941	10212	270.3	3.65	0	4	34.55	1.13	0.92	0.22	0.28	-1.35

Compound code	Molecular name	Mol ID	PubChem CID/SID	MW	AlogP	Hdon	Hacc	OB (%)	Caco-2	BBB	DL	FASA-	HL
QHB016	isoimperatorin	MOL001942	68081	270.3	3.65	0	4	45.46	0.97	0.66	0.23	0.27	-1.44
QHB017	Phellopterin	MOL002644	98608	300.33	3.64	0	5	40.19	0.98	0.48	0.28	0.24	-1.64
QHB018	beta-sitosterol	MOL000358	222284	414.79	8.08	1	1	36.91	1.32	0.99	0.75	0.23	5.36
QHB019	sitosterol	MOL000359	12303645	414.79	8.08	1	1	36.91	1.32	0.87	0.75	0.22	5.37
QHB020	(+)-Anomalin	MOL004653	5319252	426.5	5.05	0	7	46.06	0.46	0	0.66	0.36	1.03
QHB021	nodakenin	MOL004792	73191	408.44	0.28	4	9	57.12	-0.79	-1.42	0.69	0.27	7.16
QHB022	5,7-dihydroxy-2-(3-hydroxy-4-methoxyphenyl)chroman-4-one	MOL005100	676152	302.3	2.28	3	6	47.74	0.28	-0.3	0.27	0.31	16.51
QHB023	tanshinone iia	MOL007154	164676	294.37	4.66	0	3	49.89	1.05	0.7	0.4	0.31	23.56
QHB024	quercetin	MOL000098	5280343	302.25	1.5	5	7	46.43	0.05	-0.77	0.28	0.38	14.4
**9. Chuan bei mu (Fritillariae Cirrhosae Bulbus; n=13)**													
CBM001	ZINC03860434	MOL001749	7057921	390.62	7.57	0	4	43.59	1.04	0.6	0.35	0.3	3.97
CBM002	beta-sitosterol	MOL000358	222284	414.79	8.08	1	1	36.91	1.32	0.99	0.75	0.23	5.36
CBM003	sitosterol	MOL000359	12303645	414.79	8.08	1	1	36.91	1.32	0.87	0.75	0.22	5.37
CBM004	Peimisine	MOL004440	161294	427.69	3.16	2	4	57.4	0.18	-0.45	0.81	0.23	14.39
CBM005	Cyclopamine	MOL009027	442972	411.69	4.26	2	3	55.42	0.77	0	0.82	0.22	14.67
CBM006	Chuanbeinone	MOL009572	51603267	413.71	4.23	1	3	41.07	0.61	0.08	0.71	0.19	8.07
CBM007	ent-(16S)-atisan-13,17-oxide	MOL009579	50418337	288.52	4.27	0	1	47.74	1.53	1.62	0.43	0.18	8.22
CBM008	isoverticine	MOL009586	21573744	431.73	3.4	3	4	48.23	0.27	-0.44	0.67	0.16	7.9
CBM009	Korseveriline	MOL009588	16406745	431.73	3.33	3	4	35.16	0.25	-0.46	0.68	0.18	8.03
CBM010	Korseverinine	MOL009589	1.01E+08	413.71	4.36	2	3	53.51	0.34	-0.43	0.71	0.18	9.23
CBM011	verticinone	MOL009593	167691	429.71	3.21	2	4	60.07	0.42	-0.29	0.67	0.19	7.07
CBM012	sinpemine A	MOL009596	N/A	413.71	4.23	1	3	46.96	0.76	0.24	0.71	0.2	8.49
CBM013	songbeinone	MOL009599	N/A	413.71	4.23	1	3	45.35	0.63	0.02	0.71	0.19	7.64
**10. Zhe bei mu (Fritillariae Thunbergii Bulbus; n = 7)**													
ZBM001	pelargonidin	MOL001004	N/A	271.26	1.93	4	5	37.99	0.31	-0.33	0.21	0.38	0.48
ZBM002	beta-sitosterol	MOL000358	222284	414.79	8.08	1	1	36.91	1.32	0.99	0.75	0.23	5.36
ZBM003	Peimisine	MOL004440	161294	427.69	3.16	2	4	57.4	0.18	-0.45	0.81	0.23	14.39
ZBM004	Zhebeiresinol	MOL004443	N/A	280.3	0.83	1	6	58.72	0.53	0.06	0.19	0.23	3.32
ZBM005	Ziebeimine	MOL004444	3083151	413.71	4.42	2	3	64.25	0.81	0.2	0.7	0.17	7.81

Compound code	Molecular name	Mol ID	PubChem CID/SID	MW	AlogP	Hdon	Hacc	OB (%)	Caco-2	BBB	DL	FASA-	HL
ZBM006	6-Methoxyl-2-acetyl-3-methyl-1,4-naphthoquinone-8-O-beta-D-glucopyranoside	MOL004446	5319462	422.42	-0.43	4	10	33.31	-1.21	-1.67	0.57	0.3	31.01
ZBM007	Chaksine	MOL004450	120699	450.66	2.26	6	10	65.63	-0.01	-0.88	0.66	0.18	0.58
**11. Wu mei (Mume Fructus; n = 8)**													
WMA001	(2R)-5,7-dihydroxy-2-(4-hydroxyphenyl)chroman-4-one	MOL001040	667495	272.27	2.3	3	5	42.36	0.38	-0.48	0.21	0.41	16.83
WMA002	beta-sitosterol	MOL000358	222284	414.79	8.08	1	1	36.91	1.32	0.99	0.75	0.23	5.36
WMA003	kaempferol	MOL000422	5280863	286.25	1.77	4	6	41.88	0.26	-0.55	0.24	0	14.74
WMA004	Stigmasterol	MOL000449	5280794	412.77	7.64	1	1	43.83	1.44	1	0.76	0.22	5.57
WMA005	campest-5-en-3beta-ol	MOL005043	173183	400.76	7.63	1	1	37.58	1.32	0.94	0.71	0.23	4.43
WMA006	Methyl arachidonate	MOL008601	6421258	318.55	6.67	0	2	46.9	1.48	0.93	0.23	0.26	4.15
WMA007	CLR	MOL000953	5997	386.73	7.38	1	1	37.87	1.43	1.13	0.68	0.2	4.52
WMA008	quercetin	MOL000098	5280343	302.25	1.5	5	7	46.43	0.05	-0.77	0.28	0.38	14.4
**12. Xuan shen (Scrophulariae Radix; n = 9)**													
XSA001	paeoniflorin_qt	MOL001925	N/A	318.35	0.46	2	6	68.18	-0.34	-0.73	0.4	0.39	8.81
XSA002	sugiol	MOL002222	94162	300.48	4.99	1	2	36.11	1.14	0.7	0.28	0.27	14.62
XSA003	beta-sitosterol	MOL000358	222284	414.79	8.08	1	1	36.91	1.32	0.99	0.75	0.23	5.36
XSA004	sitosterol	MOL000359	12303645	414.79	8.08	1	1	36.91	1.32	0.87	0.75	0.22	5.37
XSA005	scropolioside A_qt	MOL007657	N/A	590.63	0.37	2	13	38.63	-0.99	-1.58	0.77	0.29	12.18
XSA006	14-deoxy-12(R)-sulfoandrographolide	MOL007658	N/A	414.57	1.5	3	7	62.57	-0.38	-1.1	0.42	0.3	5.16
XSA007	scropolioside D	MOL007659	6450157	722.76	-1.36	5	17	36.62	-1.97	-2.84	0.4	0.32	16.34
XSA008	scropolioside D_qt	MOL007660	N/A	560.6	0.39	2	12	33.17	-0.94	-1.58	0.82	0.34	12.27
XSA009	harpagoside_qt	MOL007662	N/A	332.38	0.17	3	6	122.87	-0.17	-0.56	0.32	0.32	10.55
**13. Huang qi (Astragali Radix; n = 20)**													
HQB001	Mairin	MOL000211	64971	456.78	6.52	2	3	55.38	0.73	0.22	0.78	0.26	8.87
HQB002	Jaranol	MOL000239	5318869	314.31	2.09	2	6	50.83	0.61	-0.22	0.29	0.29	15.5
HQB003	hederagenin	MOL000296	N/A	414.79	8.08	1	1	36.91	1.32	0.96	0.75	0	5.35
HQB004	(3S,8S,9S,10R,13R,14S,17R)-10,13-dimethyl-17-[(2R,5S)-5-propan-2-yloctan-2-yl]-	MOL000033	15976101	428.82	8.54	1	1	36.23	1.45	1.09	0.78	0	5.22

Compound code	Molecular name	Mol ID	PubChem CID/SID	MW	AlogP	Hdon	Hacc	OB (%)	Caco-2	BBB	DL	FASA-	HL
	2,3,4,7,8,9,11,12,14,15,16,17-dodecahydro-1H-cyclopenta[a]phenanthren-3-ol												
HQB005	isorhamnetin	MOL000354	5281654	316.28	1.76	4	7	49.6	0.31	-0.54	0.31	0.32	14.34
HQB006	3,9-di-O-methylnissolin	MOL000371	15689655	314.36	2.89	0	5	53.74	1.18	0.63	0.48	0	9
HQB007	5'-hydroxyiso-muronulatol-2',5'-di-O-glucoside	MOL000374	N/A	642.67	-0.95	9	16	41.72	-2.47	-3.62	0.69	0	2.52
HQB008	7-O-methylisomucronulatol	MOL000378	15689652	316.38	3.38	1	5	74.69	1.08	0.84	0.3	0	2.98
HQB009	9,10-dimethoxypterocarpan-3-O-β-D-glucoside	MOL000379	N/A	462.49	0.74	4	10	36.74	-0.63	-1.5	0.92	0	13.06
HQB010	(6aR,11aR)-9,10-dimethoxy-6a,11a-dihydro-6H-benzofurano[3,2-c]chromen-3-ol	MOL000380	14077830	300.33	2.64	1	5	64.26	0.93	0.55	0.42	0	8.49
HQB011	Bifendate	MOL000387	108213	418.38	2.56	0	10	31.1	0.15	-0.06	0.67	0	17.96
HQB012	formononetin	MOL000392	5280378	268.28	2.58	1	4	69.67	0.78	0.02	0.21	0	17.04
HQB013	isoflavanone	MOL000398	N/A	316.33	2.42	2	6	109.99	0.53	0.17	0.3	0	15.51
HQB014	Calycosin	MOL000417	5280448	284.28	2.32	2	5	47.75	0.52	-0.43	0.24	0	17.1
HQB015	kaempferol	MOL000422	5280863	286.25	1.77	4	6	41.88	0.26	-0.55	0.24	0	14.74
HQB016	FA	MOL000433	6037	441.45	0.01	7	13	68.96	-1.5	-2.59	0.71	0	24.81
HQB017	(3R)-3-(2-hydroxy-3,4-dimethoxyphenyl)chroman-7-ol	MOL000438	10380176	302.35	3.13	2	5	67.67	0.96	0.34	0.26	0	2.9
HQB018	isomucronulatol-7,2'-di-O-glucosiole	MOL000439	15689653	626.67	-0.68	8	15	49.28	-2.22	-3.36	0.62	0	0.93
HQB019	1,7-Dihydroxy-3,9-dimethoxy pterocarpene	MOL000442	5316760	314.31	3.11	2	6	39.05	0.89	-0.04	0.48	0	7.95
HQB020	quercetin	MOL000098	5280343	302.25	1.5	5	7	46.43	0.05	-0.77	0.28	0.38	14.4
**14. Fu lin (Poria; n = 15)**													
FLA001	(2R)-2-[(3S,5R,10S,13R,14R,16R,17R)-3,16-dihydroxy-4,4,10,13,14-pentamethyl-2,3,5,6,12,15,16,17-octahydro-1H-cyclopenta[a]phenanthren-17-yl]-6-methylhept-5-enoic acid	MOL000273	10743008	470.76	5.41	3	4	30.93	0.01	-0.76	0.81	0	6.81

Compound code	Molecular name	Mol ID	PubChem CID/SID	MW	AlogP	Hdon	Hacc	OB (%)	Caco-2	BBB	DL	FASA-	HL
FLA002	trametenolic acid	MOL000275	12309443	456.78	7.03	2	3	38.71	0.52	-0.14	0.8	0	7.78
FLA003	7,9(11)-dehydropachymic acid	MOL000276	15226717	526.83	6.1	2	5	35.11	0.03	-0.87	0.81	0	7.34
FLA004	Cerevisterol	MOL000279	10181133	430.74	5.15	3	3	37.96	0.28	-0.39	0.77	0	5.31
FLA005	(2R)-2-[(3S,5R,10S,13R,14R,16R,17R)-3,16-dihydroxy-4,4,10,13,14-pentamethyl-2,3,5,6,12,15,16,17-octahydro-1H-cyclopenta[a]phenanthren-17-yl]-5-isopropyl-hex-5-enoic acid	MOL000280	15225964	484.79	5.72	3	4	31.07	0.05	-0.7	0.82	0	7.42
FLA006	ergosta-7,22E-dien-3beta-ol	MOL000282	5283628	398.74	7.18	1	1	43.51	1.32	0.91	0.72	0	5.11
FLA007	Ergosterol peroxide	MOL000283	N/A	430.74	7.17	1	3	40.36	0.84	0.34	0.81	0	3.43
FLA008	(2R)-2-[(5R,10S,13R,14R,16R,17R)-16-hydroxy-3-keto-4,4,10,13,14-pentamethyl-1,2,5,6,12,15,16,17-octahydrocyclopenta[a]phenanthren-17-yl]-5-isopropyl-hex-5-enoic acid	MOL000285	9805290	482.77	5.68	2	4	38.26	0.12	-0.57	0.82	0	6.77
FLA009	3beta-Hydroxy-24-methylene-8-lanostene-21-oic acid	MOL000287	73402	470.81	7.33	2	3	38.7	0.61	-0.04	0.81	0	6.59
FLA010	pachymic acid	MOL000289	5484385	528.85	6.54	2	5	33.63	0.1	-0.57	0.81	0	9.27
FLA011	Poricoic acid A	MOL000290	5471851	498.77	5.94	3	5	30.61	-0.14	-0.93	0.76	0	8.26
FLA012	Poricoic acid B	MOL000291	5471852	484.74	5.64	3	5	30.52	-0.08	-0.87	0.75	0	8.67
FLA013	poricoic acid C	MOL000292	56668247	482.77	7.11	2	4	38.15	0.32	-0.41	0.75	0	7.73
FLA014	hederagenin	MOL000296	N/A	414.79	8.08	1	1	36.91	1.32	0.96	0.75	0	5.35
FLA015	dehydroeburicoic acid	MOL000300	15225964	453.75	6.35	1	3	44.17	0.38	-0.16	0.83	0.04	7.04
**15. Tai zi shen (Pseudostellariae Radix; n = 8)**													
TZS001	Supraene	MOL001506	638072	410.8	11.33	0	0	33.55	2.08	1.73	0.42	0.27	2.72
TZS002	acacetin	MOL001689	5280442	284.28	2.59	2	5	34.97	0.67	-0.05	0.24	0.35	17.25
TZS003	Linarin	MOL001790	5317025	592.6	-0.18	7	14	39.84	-1.68	-2.77	0.71	0.27	16.07
TZS004	beta-sitosterol	MOL000358	222284	414.79	8.08	1	1	36.91	1.32	0.99	0.75	0.23	5.36
TZS005	luteolin	MOL000006	5280445	286.25	2.07	4	6	36.16	0.19	-0.84	0.25	0.39	15.94
TZS006	Taraxerol	MOL006554	92097	426.8	7.3	1	1	38.4	1.37	1.18	0.77	0.22	2.07
TZS007	Schottenol	MOL006756	441837	414.79	8.08	1	1	37.42	1.33	0.91	0.75	0.22	5.63

Compound code	Molecular name	Mol ID	PubChem CID/SID	MW	AlogP	Hdon	Hacc	OB (%)	Caco-2	BBB	DL	FASA-	HL
TZS008	1-Monolinolein	MOL002464	6436630	354.59	5.59	2	4	37.18	0.32	-0.32	0.3	0.23	4.36

Note: AlogP: Partition coefficient between octanol and water; BBB: Blood-brain barrier; Caco-2: Human intestinal cell line Caco-2 permeability; DL: Drug-likeness; FASA-: Fractional water accessible surface area of all atoms with negative partial charge; Hacc: Hydrogen-bond acceptors; Hdon: Hydrogen-bond donors; HL: Drug half-life; MW: Molecular weight; OB: Oral bioavailability. All data was retrieved from the Traditional Chinese Medicine Systems Pharmacology Database and Analysis Platform (https://tcmsp-e.com/). This table was retrieved from the supplementary file of our previously published paper. Detailed information is provided as follow: Li, H., Komori, A., Li, M., Chen, X., Yang, A. W. H., Sun, X., ... & Zhou, L. (2023). Multi-ligand molecular docking, simulation, free energy calculations and wavelet analysis of the synergistic effects between natural compounds baicalein and cubebin for the inhibition of the main protease of SARS-CoV-2. *Journal of Molecular Liquids*, *374*, 121253.

Table S3. Simplified Molecular-input Line-entry System formats of 238 compounds identified from Toujie Quwen Granules.

Compounds	Simplified Molecular-input Line-entry System formats
CBM001	c1(c(cccc1)C(=O)OC[C@@H](CCCC)CC)C(=O)OC[C@@H](CCCC)CC
CBM002	[C@@]12([C@H]([C@H]3[C@@H]([C@@]4(C(=CC3)C[C@H](CC4)O)C)CC2)CC[C@@H]1[C@H](C)CC[C@H](C(C)C)CC)C
CBM003	[C@@]12([C@H]([C@H]3[C@@H]([C@@]4(C(=CC3)C[C@@H](CC4)O)C)CC2)CC[C@@H]1[C@H](C)CC[C@H](C(C)C)CC)C
CBM004	O1[C@@]2(C(=C3[C@H]([C@H]4[C@@H]([C@@]5([C@@H](C(=O)C4)C[C@H](CC5)O)C)C3)CC2)C)[C@@H]([C@@H]2NC[C@H](C[C@@H]12)C)C
CBM005	O1[C@@]2([C@@H]([C@@H]3NC[C@H](C[C@@H]13)C)C)CC[C@H]1[C@H]3[C@@H]([C@@]4(C(=CC3)C[C@H](CC4)O)C)CC1=C2C
CBM006	N12C[C@H]3[C@H]4[C@H]([C@H]5[C@H]([C@]6(CC[C@@H](C[C@@H]6C(=O)C5)O)C)C4)CC[C@H]3[C@H]([C@H]2CC[C@@H](C1)C)C
CBM007	O1[C@@H]2[C@H]3C[C@H]4[C@](C[C@H]3C1)(C2)CC[C@H]1[C@]4(CCCC1(C)C)C
CBM008	N12C[C@H]3[C@H]4[C@H]([C@H]5[C@@H]([C@@]6([C@@H]([C@@H](C5)O)C[C@H](CC6)O)C)C4)CC[C@@H]3[C@]([C@@H]1CC[C@@H](C2)C)(C)O
CBM009	N12C[C@H]3[C@H]4[C@]([C@H]5[C@@H]([C@@]6([C@@H]([C@@H](C5)O)C[C@@H](CC6)O)C)C4)(CC[C@@H]3[C@H]([C@@H]1CC[C@H](C2)C)C)O
CBM010	N12C[C@H]3[C@H]4[C@H](C5=C([C@@]6([C@@H]([C@H](C5)O)C[C@@H](CC6)O)C)C4)CC[C@@H]3[C@H]([C@@H]1CC[C@H](C2)C)C
CBM011	O=C1[C@@H]2[C@@]([C@@H]3[C@H]([C@H]4[C@H]([C@H]5[C@H](CC4)[C@]([C@H]4N(C5)C[C@H](CC4)C)(C)O)C3)C1)(CC[C@@H](C2)O)C
CBM012	O=C1[C@@H]2[C@@]([C@H]3[C@H]([C@H]4[C@H]([C@H]5[C@@H](CC4)[C@H]([C@H]4N(C5)C[C@H](CC4)C)C)C3)C1)(CC[C@@H](C2)O)C
CBM013	[C@@H]1(CC[C@]2([C@H](C1)C(=O)C[C@H]1[C@@H]3CC[C@H]4[C@H](C)[C@@H]5CC[C@H](C)CN5C[C@H]4[C@@H]3C[C@H]21)C)O
CHA001	C(CCCCCCOC(=O)C)C/C=C/C/C=C/CCCCC
CHA002	o1c2cc(c(c(c2c(=O)cc1c1ccccc1)O)O)O[C@@H]1O[C@@H]([C@H]([C@@H]([C@H]1O)O)O)C(=O)O
CHA003	[C@@]12([C@H]([C@H]3[C@@H]([C@@]4(C(=CC3)C[C@H](CC4)O)C)CC2)CC[C@@H]1[C@H](C)/C=C/[C@H](C(C)C)CC)C
CHA004	o1c(c(c(=O)c2c1cc(cc2O)O)O)c1cc(c(cc1)O)OC

Compounds	Simplified Molecular-input Line-entry System formats
CHA005	o1c2c(c(=O)c(c1c1ccc(cc1)O)O)c(cc(c2)O)O
CHA006	o1c2c(c(c(c(c2)OC)OC)OC)c(=O)c(c1c1cc(c(c(c1)OC)OC)OC)OC
CHA007	o1c2c(c(c(c(c2)OC)OC)O)c(=O)cc1c1c(cc(c(c1)OC)O)O
CHA008	O1[C@@H]([C@@H]([C@H](C1)Cc1cc2OCOc2cc1)Cc1cc2OCOc2cc1)O
CHA009	[C@@]123[C@H]([C@@H]([C@]([C@]45[C@H]1CC[C@H]([C@H]4O)C(=C)C5=O)(OC3)O)O)C(CCC2)(C)C
CHA010	C1CCC(=O)N2[C@@H]1[C@H]1C[C@@H](C2)[C@@H]2N(C1)CC[C@@H](C2)O
CHA011	o1c(cc2c1cc(c(c2)O)OC)c1c(cc(cc1)OC)O
CHA012	C(=C\C=C(\C)/C=C/C1=C(C)CCCC1(C)C)/C(=C\CCC(O)(O)O)/C
CHA013	O1C([C@@H]([C@@H](c2c1ccc1c2oc(=O)cc1)OC(=O)/C(=C/C)/C)OC(=O)/C(=C/C)/C)(C)C
CHA014	[C@]12([C@]3(C=C[C@@H]4[C@]1(CC[C@@H]1[C@@]4(CC[C@@H]([C@@]1(C)CO)O)C)C)[C@@H]1[C@]([C@H](C2)O)(CO3)CCC(C1)(C)C)C
CHA015	[C@@]12([C@H](C3=CC[C@H]4[C@@]([C@H]3CC2)(CC[C@@H](C4)O)C)CC[C@@H]1[C@H](C)/C=C/[C@@H](C(C)C)CC)C
CHA016	C1(=C(C=C2[C@H](O1)C=C(C=C2O)O)O)c1cc(c(c(c1)O)O)OC
CHA017	o1c2c(c(=O)c(c1c1cc(c(cc1)O)O)O)c(cc(c2)O)O
DQY001	[C@@]12([C@H]([C@H]3[C@@H]([C@@]4(C(=CC3)C[C@H](CC4)O)C)CC2)CC[C@@H]1[C@H](C)/C=C/[C@@H](C(C)C)CC)C
DQY002	O=C1/C(=C/2\Nc3c(C2=O)cccc3)/Nc2c1cccc2
DQY003	O=c1n2c([nH]c3c1cccc3)c(c1c2cccc1)[C@@H]1Nc2c(C1=O)cccc2
DQY004	C\1(=C\CN2CCC[C@H]2C(=O)O)/C[C@H](NC(=C1)C(=O)O)C(=O)O
DQY005	O=C1/C(=C\2/c3c(NC2=O)cccc3)/Nc2c1cccc2
DQY006	o1c2c3c(c(c(cc3oc(=O)c2c2c1cc(cc2)O)O)CC=C(C)C)OC
DQY007	N([C@@H](S[C@@H]1O[C@@H]([C@H]([C@@H]([C@H]1O)O)O)CO)Cc1c2c([nH]c1)cccc2)OS(=O)(=O)O
DQY008	C12=CC[C@@H]3[C@H]([C@@]1(CC[C@H](C2)O)C)CC[C@@]1([C@@H]3CC[C@H]1[C@@H](C)CC[C@@H](CC)C(C)C)C
DQY009	o1c2c(c(c(c(c2)O)[C@@H]2O[C@@H]([C@H]([C@@H]([C@H]2O)O)O)CO)O)c(=O)cc1c1ccc(cc1)O
DQY010	[C@@]12([C@H]([C@H]3[C@@H]([C@@]4(C(=CC3)C[C@H](CC4)O)C)CC2)CC[C@@H]1[C@H](C)CC[C@H](C(C)C)CC)C
FLA001	[C@@]12([C@@](C[C@H]([C@@H]1[C@@H](CCC=C(C)C)C(=O)O)O)(C1=CC[C@@H]3[C@@](C1=CC2)(CC[C@@H](C3(C)C)O)C)C)C
FLA002	[C@@]12([C@](C3=C([C@@]4([C@H](C([C@H](CC4)O)(C)C)CC3)C)CC2)(CC[C@@H]1[C@@H](CCC=C(C)C)C(=O)O)C)C

Compounds	Simplified Molecular-input Line-entry System formats
FLA003	[C@@]12([C@@](C[C@H]([C@@H]1[C@@H](CCC(=C)C(C)C)C(=O)O)O)(C1=CC[C@@H]3[C@@](C1=CC2)(CC[C@@H](C3(C)C)OC(=O)C)C)C)C
FLA004	[C@@]12([C@H](C3=C[C@H]([C@@]4([C@@]([C@H]3CC2)(CC[C@@H](C4)O)C)O)O)CC[C@@H]1[C@H](C)/C=C/[C@H](C)C(C)C)C
FLA005	[C@@]12([C@@](C[C@H]([C@@H]1[C@@H](CCC(=C)C(C)C)C(=O)O)O)(C1=CC[C@@H]3[C@@](C1=CC2)(CC[C@@H](C3(C)C)O)C)C)C
FLA006	[C@@]12([C@H](C3=CC[C@@H]4[C@@]([C@H]3CC2)(CC[C@@H](C4)O)C)CC[C@@H]1[C@H](C)/C=C/[C@H](C)C(C)C)C
FLA007	C1C[C@H]2[C@@]3(C[C@@H]1O)CC[C@@]1([C@@]2(CC[C@]2([C@H]1CC[C@@H]2[C@H](C)CC[C@H](C)C(C)C)C)C)OO3
FLA008	O=C1C([C@H]2[C@@](C3=CC[C@]4([C@@](C[C@H]([C@@H]4[C@@H](CCC(=C)C(C)C)C(=O)O)O)(C3=CC2)C)C)(CC1)C)(C)C
FLA009	[C@@]12([C@](C3=C([C@@]4([C@H](C([C@H](CC4)O)(C)C)CC3)C)CC2)(CC[C@@H]1[C@@H](CCC(=C)C(C)C)C(=O)O)C)C
FLA010	[C@@]12([C@](C3=C([C@@]4([C@H](C([C@H](CC4)OC(=O)C)(C)C)CC3)C)CC2)(C[C@H]([C@@H]1[C@@H](CCC(=C)C(C)C)C(=O)O)O)C)C
FLA011	[C@@]12([C@@](C[C@H]([C@@H]1[C@@H](CCC(=C)C(C)C)C(=O)O)O)(C1=CC[C@H]([C@](C1=CC2)(C)CCC(=O)O)C(=C)C)C)C
FLA012	[C@@]12([C@@](C[C@H]([C@@H]1[C@@H](CCC=C(C)C)C(=O)O)O)(C1=CC[C@H]([C@](C1=CC2)(C)CCC(=O)O)C(=C)C)C)C
FLA013	[C@@]12([C@@](CC[C@@H]1[C@@H](CCC(=C)C(C)C)C(=O)O)(C1=CC[C@H]([C@](C1=CC2)(C)CCC(=O)O)C(=C)C)C)C
FLA014	C1C[C@H](CC2=CC[C@@H]3[C@H]4CC[C@@H]([C@@]4(C)CC[C@H]3[C@@]12C)[C@H](C)/C=C/[C@@H](CC)C(C)C)O
FLA015	[C@@]12([C@@](C[C@H]([C@@H]1[C@@H](CCC(=C)C(C)C)C(=O)O)O)(C1=CC[C@@H]3[C@@](C1=CC2)(CC[C@@H](C3(C)C)O)C)C)C
HQA001	o1c2c(c(=O)cc1c1ccc(cc1)OC)c(cc(c2)O)O
HQA002	o1c2c(c(=O)cc1c1ccccc1)c(cc(c2OC)O)O
HQA003	O1[C@H](CC(=O)c2c1cc(cc2OC)O)c1ccccc1
HQA004	o1c2c(c(c(c(c2)O)O)O)c(=O)cc1c1ccccc1
HQA005	o1c2c(c(=O)cc1c1c(cccc1)O)c(cc(c2O)OC)O
HQA006	o1c2c(c(c(c(c2OC)O)OC)O)c(=O)cc1c1c(c(ccc1O)O)OC

Compounds	Simplified Molecular-input Line-entry System formats
HQA007	c12c(C(=O)C[C@@H](O1)c1ccc(cc1)O)c(c(c(c2)O)O)O
HQA008	C(=O)(/C=C\c1c(cccc1O)O)c1c(cc(cc1OC)O)O
HQA009	O1[C@@H](CC(=O)c2c1cc(c(c2O)O)O)c1ccccc1
HQA010	O1[C@H](CC(=O)c2c1cc(cc2O)O)c1cc(c(cc1)O)O
HQA011	o1c2c(c(c(c(c2)OC)OC)O)c(=O)cc1c1ccc(cc1)OC
HQA012	o1c2c(c(=O)cc1c1c(cccc1O)O)c(cc(c2OC)OC)O
HQA013	o1c2c(c(=O)cc1c1c(cccc1O)O)c(cc(c2)O)O
HQA014	c1(cc(c2c(c1O)C(=O)C[C@H](O2)c1ccccc1)O)OC
HQA015	o1c2c(c(c(c(c2OC)OC)OC)O)c(=O)cc1c1c(cccc1O)OC
HQA016	o1c2c(c(c(c(c2)O)OC)O)c(=O)cc1c1ccccc1
HQA017	o1c2c(c(cc(c2OC)OC)O)c(=O)cc1c1c(cccc1)O
HQA018	o1c2c(c(=O)cc1c1ccc(cc1)O)c(cc(c2OC)O)O
HQA019	c1(c(c(c2c(c1OC)oc(cc2=O)c1cc(ccc1O)OC)O)OC)OC
HQA020	O1[C@H](CC(=O)c2c1cc(c(c2O)OC)O)c1ccccc1
HQA021	[C@@]12([C@H]([C@H]3[C@@H]([C@@]4(C(=CC3)C[C@H](CC4)O)C)CC2)CC[C@@H]1[C@H](C)CC[C<br>@H](C(C)C)CC)C
HQA022	[C@@]12([C@H]([C@H]3[C@@H]([C@@]4(C(=CC3)C[C@@H](CC4)O)C)CC2)CC[C@@H]1[C@H](C)CC[<br>C@H](C(C)C)CC)C
HQA023	o1c2c(c(=O)cc1c1ccccc1)c(cc(c2O)O)O
HQA024	o1c2c(c(c(c(c2OC)OC)OC)O)c(=O)cc1c1c(cccc1)O
HQA025	O1[C@H]([C@H](Cc2c1cc(cc2O)O)O)c1cc(c(cc1)O)O
HQA026	[C@@]12([C@H]([C@H]3[C@@H]([C@@]4(C(=CC3)C[C@H](CC4)O)C)CC2)CC[C@@H]1[C@H](C)/C=C/[<br>C@H](C(C)C)CC)C
HQA027	O1c2cc3[C@H]4N(CCc3cc2OC1)C=c1c(=C4)ccc2OCOc12
HQA028	c1(c(cccc1)C(=O)OC[C@H](CCCC)CC)C(=O)OC[C@H](CCCC)CC
HQA029	C(C/C=C(\C)/CC/C=C(\C)/CCC=C(C)C)/C=C(\C)/CC/C=C(\C)/CCC=C(C)C
HQA030	c1(c(cccc1)C(=O)OCCCCCC(C)C)C(=O)OCCCCCC(C)C
HQA031	O1c2c3=CN4CCc5c([C@H]4C=c3ccc2OC1)cc(c(c5)OC)OC
HQA032	o1c2c(c(cc(c2OC)OC)O)c(=O)cc1c1ccccc1
HQA033	C(CCCCCCCC(=O)OC)C/C=C/C=C/CCCCCC
HQA034	O1[C@H](CC(=O)c2c1cc(c(c2O)OC)O)c1ccc(cc1)O
HQA035	[C@@H]1(CC(=O)c2c(cc(c(c2O1)OC)O)O)c1ccc(cc1)O

Compounds	Simplified Molecular-input Line-entry System formats
HQA036	o1c2c(c(=O)cc1c1c(cccc1O)OC)c(cc(c2OC)OC)O
HQB001	[C@@]12([C@]3([C@@H]([C@@H]4[C@@](CC3)(CC[C@H]4C(=C)C)C(=O)O)CC[C@@H]1[C@@]1([C@@H](CC2)C([C@H](CC1)O)(C)C)C)C)C
HQB002	o1c2c(c(=O)c(c1c1ccc(cc1)O)OC)c(cc(c2)OC)O
HQB003	C1C[C@H](CC2=CC[C@@H]3[C@H]4CC[C@@H]([C@@]4(C)CC[C@H]3[C@@]12C)[C@H](C)/C=C/[C@@H](CC)C(C)C)O
HQB004	[C@@]12([C@H]([C@H]3[C@@H]([C@@]4(C(=CC3)C[C@H](CC4)O)C)CC2)CC[C@@H]1[C@H](C)CC[C@@H](C(C)C)CCC)C
HQB005	o1c(c(c(=O)c2c1cc(cc2O)O)O)c1cc(c(cc1)O)OC
HQB006	O1[C@@H]2[C@H](c3c1c(c(cc3)OC)OC)COc1c2ccc(c1)OC
HQB007	c1(c(c(c(c(c1)O[C@@H]1O[C@@H]([C@H]([C@@H]([C@H]1O)O)O)CO)OC)OC)O[C@H]1O[C@@H]([C@H]([C@@H]([C@H]1O)O)O)CO)[C@H]1Cc2ccc(cc2OC1)O
HQB008	O1C[C@H](Cc2c1cc(cc2)OC)c1c(c(c(cc1)OC)OC)O
HQB009	C1[C@@H]2[C@H](c3c(O1)cc(cc3)O[C@H]1[C@@H]([C@H]([C@@H]([C@H](O1)CO)O)O)O)Oc1c2ccc(c1OC)OC
HQB010	O1[C@@H]2[C@H](c3c1c(c(cc3)OC)OC)COc1c2ccc(c1)O
HQB011	O1c2c(c(cc(c2OC1)OC)C(=O)OC)c1c2OCOc2c(cc1C(=O)OC)OC
HQB012	o1c2c(c(=O)c(c1)c1ccc(cc1)OC)ccc(c2)O
HQB013	C1[C@@H](C(=O)c2c(O1)ccc(c2OC)OC)c1ccc(cc1O)O
HQB014	o1c2c(c(=O)c(c1)c1cc(c(cc1)OC)O)ccc(c2)O
HQB015	o1c2c(c(=O)c(c1c1ccc(cc1)O)O)c(cc(c2)O)O
HQB016	c1(ccc(cc1)NCc1[nH]c2c(N[C@H](NC2=O)N)[nH]c1)C(=O)N[C@@H](CCC(=O)O)C(=O)O
HQB017	O1C[C@H](Cc2c1cc(cc2)O)c1c(c(c(cc1)OC)OC)O
HQB018	O1C[C@H](Cc2c1cc(cc2)O[C@@H]1O[C@@H]([C@H]([C@@H]([C@H]1O)O)O)CO)c1c(c(c(cc1)OC)OC)O[C@@H]1O[C@@H]([C@H]([C@@H]([C@H]1O)O)O)CO
HQB019	o1c2c(c3c1cc(cc3O)OC)CO[C@H]1C2=C(CC(=C1)OC)O
HQB020	o1c2c(c(=O)c(c1c1cc(c(cc1)O)O)O)c(cc(c2)O)O
JYH001	C(CCCCCC(=O)OCC)C/C=C\C/C=C\CCCCC
JYH002	C(CCCCCC(=O)OCC)C/C=C/C/C=C/C/C=C/CC
JYH003	C(=C\C=C(/C)\C=C\C=C(/C)\CC/C=C(\C)/CCC=C(C)C)/C=C(\C)/CC/C=C(\C)/CC=C=C(C)CCC=C(C)C
JYH004	O1[C@H](CC(=O)c2c1cc(cc2O)O)c1cc(c(cc1)O)O
JYH005	[C@H]12C[C@@H]3C(=CO[C@H]([C@@H]3C=C)O)C(=O)N1[C@H](CO2)C(=O)O

Compounds	Simplified Molecular-input Line-entry System formats
JYH006	[C@@H]1([C@@H]([C@@H](C(=CO1)C(=O)OC)CC(OCCCCC)OCCCCC)C=C)O
JYH007	C(=C\C=C(/C)\C=C\C=C(/C)\C=C\C1=C(CCCC1(C)C)C)/C=C(\C)/C=C/C=C(\C)/C=C/C1=C(CCCC1(C)C)C
JYH008	[C@@]12([C@H]([C@@H]3[C@H]([C@@]4(C(=CC3)C[C@H](CC4)O)C)CC2)CC[C@@H]1[C@H](C)/C=C/[C@H](C(C)C)CC)C
JYH009	o1c(cc(=O)c2c1cc(cc2O)O)c1cc(c(cc1)O)OC
JYH010	C(=C\C=C\C=C(/C)\C=C\C=C(/C)\C=C/C1=C(CCCC1(C)C)C)(\C)/C=C/C=C(\C)/C=C/C1=C(C[C@@H](CC1(C)C)O)C
JYH011	C(=C\C=C\C(=C\C=C\C(=C\C=C/1\C(CC(=O)C=C1C)(C)C)\C)\C)/C(=C/C=C/C(=C/C=C\1/C(CC(=O)C=C1C)(C)C)/C)/C
JYH012	o1c(cc(=O)c2c1cc(cc2O)OC)c1cc(c(c(c1)OC)OC)OC
JYH013	[C@@H]1(C[C@@]2(C(=CO[C@H]([C@@]2(C)C=C)OO[C@@H]2[C@@H]([C@H]([C@@H]([C@@H](O2)CO)O)O)O)C(=O)O1)C)OC
JYH014	[C@@H]1([C@@H]([C@@H](C(=CO1)C(=O)OC)C[C@H]1O[C@H]([C@@H](CO1)O)[C@@H]([C@@H](C=O)O)O)C=C)O[C@H]1[C@@H]([C@H]([C@@H]([C@H](O1)CO)O)O)O
JYH015	C(=C(\C=O)/[C@@H]1[C@H]([C@@H](OC=C1C(=O)OC)O)C=C)/C[C@H]1[C@@H]([C@H](OC=C1C(=O)OC)O)C=C
JYH016	[C@H]1([C@@H]([C@H](OC=C1C(=O)OC)OO)C=C)C[C@H]1O[C@@H]([C@H](O1)C)C
JYH017	S1[C@@H]2N(C(=O)C3=CO[C@@H]([C@H]([C@@H]3C2)C=C)O[C@@H]2O[C@H]([C@@H]([C@H]([C@H]2O)O)O)CO)CC1
JYH018	[C@@H]1([C@H]([C@@H](OC=C1C(=O)OC)OO[C@H]1[C@@H]([C@H]([C@@H]([C@H](O1)CO)O)O)O)C=C)CC(=O)OC
JYH019	[C@@]12([C@H]([C@H]3[C@@H]([C@@]4(C(=CC3)C[C@H](CC4)O)C)CC2)CC[C@@H]1[C@H](C)CC[C@H](C(C)C)CC)C
JYH020	o1c2c(c(=O)c(c1c1ccc(cc1)O)O)c(cc(c2)O)O
JYH021	[C@@]12([C@H]([C@H]3[C@@H]([C@@]4(C(=CC3)C[C@H](CC4)O)C)CC2)CC[C@@H]1[C@H](C)/C=C/[C@H](C(C)C)CC)C
JYH022	o1c2c(c(=O)cc1c1cc(c(cc1)O)O)c(cc(c2)O)O
JYH023	o1c2c(c(=O)c(c1c1cc(c(cc1)O)O)O)c(cc(c2)O)O
LQA001	o1c2c(c(=O)cc1c1ccccc1)c(cc(c2OC)O)O
LQA002	C1C[C@@H](C([C@@H]2[C@]1([C@@H]1[C@@](CC2)([C@]2([C@H](CC1)[C@H](CC2)[C@@](C)(O)CCC=C(C)C)C)C)C)(C)C)OC(=O)C
LQA003	[C@@H]1([C@@H](Cc2c([C@@H]1c1cc(c(cc1)O)OC)cc(c(c2)OC)O)CO)CO

Compounds	Simplified Molecular-input Line-entry System formats
LQA004	O1C[C@@H]([C@H](C1=O)Cc1cc(c(cc1)OC)OC)Cc1cc(c(cc1)OC)OC
LQA005	O1[C@@H]([C@H]2[C@@H](C1)[C@@H](OC2)c1cc(c(cc1)OC)OC)c1cc(c(cc1)O)OC
LQA006	c1(cc(c(cc1)O[C@H]1O[C@@H]([C@H]([C@@H]([C@H]1O)O)O)CO)OC)[C@@H]1[C@@H]2[C@H](CO1)[C@@H](OC2)c1cc(c(cc1)OC)OC
LQA007	O1[C@@H]([C@@H]2[C@@H]([C@H](OC2)c2cc(c(cc2)O)OC)C1)c1cc(c(cc1)OC)OC
LQA008	O1[C@@H]([C@H]2[C@@H](C1)[C@@H](OC2)c1cc(c(cc1)OC)O)c1cc(c(cc1)OC)OC
LQA009	C1C[C@@H](C([C@@H]2[C@]1([C@@H]1[C@@](CC2)([C@]2([C@H](CC1)[C@H](CC2)[C@]1(OC([C@H](CC1)O)(C)C)C)C)C)C)(C)C)OC(=O)C
LQA010	[C@@]12([C@]3([C@@H]([C@@H]4[C@@](CC3)(CC[C@H]4C(=C)C)C(=O)O)CC[C@@H]1[C@@]1([C@@H](CC2)C([C@H](CC1)O)(C)C)C)C)C
LQA011	[C@H]12[C@@H]([C@H](OC2)c2cc(c(cc2)OC)OC)CO[C@@H]1c1c(cc(cc1)O)OC
LQA012	[C@H]1(OC[C@@H]2[C@H]1CO[C@H]2c1cc(c(cc1)O)OC)c1ccc(c(c1)OC)OC
LQA013	[C@]12([C@H]([C@@]3([C@H](CC2)C([C@@H](CC3)OC(=O)C)(C)C)C)CC=C2[C@@]1(CC[C@]1([C@@H]2CC(CC1)(C)C)C)C)C
LQA014	O=C1[C@]2([C@]([C@H](C[C@@]1(C(=C(C2=O)CC=C(C)C)O)CC=C(C)C)CC=C(C)C)(C)CCC=C(C)C)C(=O)C(C)C
LQA015	O=C1[C@]2([C@]([C@H](C[C@@]1(C(=C(C2=O)CC=C(C)C)O)CC=C(C)C)CC=C(C)C)(C)CCC=C(C)C)C(=O)[C@@H](C)CC
LQA016	C1[C@H](C(=O)C2=CC[C@@H]3[C@@H]([C@@]2(C1)C)CC[C@@]1([C@@H]3CC[C@H]1[C@H](C)C[C@H]1[C@@H]([C@@H]1C(C)C)C)C)O
LQA017	o1c2c(c(c(c(c2)OC)OC)OC)c(=O)c2c1ccc(c2)O
LQA018	[C@@]12([C@H]([C@H]3[C@@H]([C@@]4(C(=CC3)C[C@H](CC4)O)C)CC2)CC[C@@H]1[C@H](C)CC[C@H](C(C)C)CC)C
LQA019	o1c2c(c(=O)c(c1c1ccc(cc1)O)O)c(cc(c2)O)O
LQA020	c1(cc(c(cc1)O[C@@H]1O[C@@H]([C@H]([C@@H]([C@H]1O)O)O)CO)OC)C[C@@H]1[C@H](COC1=O)Cc1cc(c(cc1)OC)OC
LQA021	o1c2c(c(=O)cc1c1cc(c(cc1)O)O)c(cc(c2)O)O
LQA022	O1c2cc3[C@H](N(CCc3cc2OC1)C)[C@@H]1OC(=O)c2c1ccc1c2OCO1
LQA023	o1c2c(c(=O)c(c1c1cc(c(cc1)O)O)O)c(cc(c2)O)O
QHA001	o1c2c(c(c(c(c2)OC)OC)O)c(=O)c(c1c1cc(c(cc1)OC)O)O
QHA002	o1c(c(c(=O)c2c1cc(cc2O)O)O)c1cc(c(cc1)O)OC

Compounds	Simplified Molecular-input Line-entry System formats
QHA003	[C@@]12([C@H]([C@H]3[C@@H]([C@@]4(C(=CC3)C[C@@H](CC4)O)C)CC2)CC[C@@H]1[C@H](C)CC[C@H](C(C)C)CC)C
QHA004	o1c(c(c(=O)c2c1cc(cc2O)O)O)c1cc(c(cc1)OC)O
QHA005	o1c2c(c(=O)c(c1c1cc(c(cc1)O)O)O)c(c(c(c2)O)OC)O
QHA006	o1c2c(c(=O)c(c1c1ccc(cc1)O)O)c(cc(c2)O)O
QHA007	[C@@]12([C@H]([C@H]3[C@@H]([C@@]4(C(=CC3)C[C@H](CC4)O)C)CC2)CC[C@@H]1[C@H](C)/C=C/[C@H](C(C)C)CC)C
QHA008	o1c2c(c(c(c(c2)OC)OC)O)c(=O)cc1c1c(cc(c(c1)OC)O)O
QHA009	o1c2c(c(=O)c(c1c1cc(c(cc1)OC)OC)OC)c(c(c(c2)OC)OC)O
QHA010	o1c2c(c(=O)cc1c1cc(c(cc1)O)O)c(cc(c2)O)O
QHA011	o1c2c(c(c(c(c2)OC)OC)O)c(=O)cc1c1ccc(cc1)O
QHA012	O1O[C@@]2(CC[C@@H]3[C@@]41[C@@H](CC[C@H]3C)C(=C)C(=O)O[C@@H]4O2)C
QHA013	c1c(cc2c(c1O)c(=O)cc(o2)c1ccc(cc1)O)O
QHA014	o1c2c(c(c(c(c2)OC)OC)O)c(=O)cc1c1cc(c(cc1)O)O
QHA015	c1(cc(=O)c2c(o1)cc(cc2O)O)c1ccc(cc1)O
QHA016	o1c2c(c(=O)c(c1c1cc(c(cc1)O)O)OC)c(c(c(c2)O)OC)O
QHA017	[C@H](NC(=O)c1ccccc1)(Cc1ccccc1)C(=O)N[C@@H](Cc1ccccc1)COC(=O)C
QHA018	c1(cc(=O)c2c(cc(cc2o1)O)O)c1ccc(cc1)O
QHA019	[C@@]123[C@H]4O[C@](OO3)(CC[C@H]2[C@@H](CC[C@H]1[C@H](C(=O)O4)C)C)C
QHA020	[C@@H]12O[C@@H]([C@@H]([C@H]3[C@@]41[C@@H]([C@@H](CC3)C)CC[C@](O2)(OO4)C)C)O
QHA021	O1[C@]2(CC[C@@H]3[C@@]4([C@@H]1OC(=O)[C@@H]([C@@H]4CC[C@H]3C)C)O2)C
QHA022	o1c2c(c(=O)c(c1c1cc(c(cc1)O)O)O)c(cc(c2)O)O
QHB001	O1[C@@H]([C@@H](c2c1ccc1c2oc(=O)cc1)O)C(C)(C)O
QHB002	O1C([C@H](Cc2c1cc1oc(=O)ccc1c2)OC(=O)C=C(C)C)(C)C
QHB003	O1C([C@H]([C@H](c2c1ccc1c2oc(=O)cc1)OC(=O)CC(C)C)OC(=O)/C(=C\C)/C)(C)C
QHB004	O1C([C@@H]([C@H](c2c1ccc1c2oc(=O)cc1)OC(=O)C)OC(=O)/C(=C\C)/C)(C)C
QHB005	n12c3c(CCN(C3=C[C@@H]3C(=CNC=C3)[C@H]1C)C)c1c2cccc1
QHB006	O1[C@@H]([C@H]([C@@H]([C@H]([C@@H]1Oc1cc2oc(=O)ccc2cc1)O)O)O)CO
QHB007	O1C([C@@H]([C@@H](c2c1ccc1c2oc(=O)cc1)OC(=O)/C(=C/C)/C)OC(=O)C)(C)C
QHB008	O1[C@@H]([C@@H]([C@@H]([C@H]([C@@H]1OC(C)(C)[C@H](O)Cc1c2oc(=O)ccc2c(cc1OC)OC)O)O)O)CO[C@H]1OC[C@]([C@@H]1O)(O)CO
QHB009	o1c2c(c(cc(c2ccc1=O)OC)OC)C[C@@H](O)C(C)(C)O

Compounds	Simplified Molecular-input Line-entry System formats
QHB010	Clc1cc2[C@@]3([C@@H](N4[C@@]5(SS[C@@](N(C5=O)C)(C4=O)C)[C@@H]3O)N(c2c(c1OC)OC)C)O
QHB011	O1C([C@@H](Cc2c1ccc1c2oc(=O)cc1)OC(=O)/C(=C\C)/C)(C)C
QHB012	O1[C@@H](Cc2c1c(c1oc(=O)ccc1c2)O[C@@H]1O[C@@H]([C@H]([C@H]([C@H]1O)O)O)CO)C(C)(C)O
QHB013	O1[C@@H](Cc2c1c(c1oc(=O)ccc1c2)O)C(C)(C)O
QHB014	C(=C\COc1cc2oc(=O)ccc2cc1)(\C)/CC/C=C(\C)/CCC=C(C)C
QHB015	o1c2c(c3oc(=O)ccc3cc2cc1)OCC=C(C)C
QHB016	o1c2c(c(c3c(oc(=O)cc3)c2)OCC=C(C)C)cc1
QHB017	o1c2c(c(c3c(oc(=O)cc3)c2OCC=C(C)C)OC)cc1
QHB018	[C@@]12([C@H]([C@H]3[C@@H]([C@@]4(C(=CC3)C[C@H](CC4)O)C)CC2)CC[C@@H]1[C@H](C)CC[C@H](C(C)C)CC)C
QHB019	[C@@]12([C@H]([C@H]3[C@@H]([C@@]4(C(=CC3)C[C@@H](CC4)O)C)CC2)CC[C@@H]1[C@H](C)CC[C@H](C(C)C)CC)C
QHB020	O1C([C@@H]([C@@H](c2c1ccc1c2oc(=O)cc1)OC(=O)/C(=C/C)/C)OC(=O)/C(=C/C)/C)(C)C
QHB021	C(C)(C)(O[C@@H]1O[C@@H]([C@H]([C@@H]([C@H]1O)O)O)CO)[C@@H]1Oc2c(C1)cc1c(c2)oc(=O)cc1
QHB022	O1[C@H](CC(=O)c2c1cc(cc2O)O)c1cc(c(cc1)OC)O
QHB023	o1c2c3c(c4c(C(CCC4)(C)C)cc3)C(=O)C(=O)c2c(c1)C
QHB024	o1c2c(c(=O)c(c1c1cc(c(cc1)O)O)O)c(cc(c2)O)O
SCG001	[C@@]12([C@H]([C@H]3[C@@H]([C@@]4(C(=CC3)C[C@H](CC4)O)C)CC2)CC[C@@H]1[C@H](C)CC[C@H](C(C)C)CC)C
SCG002	[C@@]12([C@H]([C@H]3[C@@H]([C@@]4(C(=CC3)C[C@H](CC4)O)C)CC2)CC[C@@H]1[C@H](C)/C=C/[C@H](C(C)C)CC)C
SCG003	C1Cc2c(c3c1cc(cc3O)OC)c(ccc2)O
TZS001	C(C/C=C(\C)/CC/C=C(\C)/CCC=C(C)C)/C=C(\C)/CC/C=C(\C)/CCC=C(C)C
TZS002	o1c2c(c(=O)cc1c1ccc(cc1)OC)c(cc(c2)O)O
TZS003	O1[C@@H]([C@H]([C@@H]([C@H]([C@@H]1Oc1cc2oc(cc(=O)c2c(c1)O)c1ccc(cc1)OC)O)O)O)CO[C@@H]1O[C@H]([C@@H]([C@H]([C@H]1O)O)O)C
TZS004	[C@@]12([C@H]([C@H]3[C@@H]([C@@]4(C(=CC3)C[C@H](CC4)O)C)CC2)CC[C@@H]1[C@H](C)CC[C@H](C(C)C)CC)C
TZS005	o1c2c(c(=O)cc1c1cc(c(cc1)O)O)c(cc(c2)O)O
TZS006	[C@@H]12[C@@]3([C@H](C([C@H](CC3)O)(C)C)CC[C@]1(C1=CC[C@@]3([C@H]([C@@]1(CC2)C)CC(CC3)(C)C)C)C)C

Compounds	Simplified Molecular-input Line-entry System formats
TZS007	[C@@]12([C@H](C3=CC[C@@H]4[C@@]([C@H]3CC2)(CC[C@@H](C4)O)C)CC[C@@H]1[C@H](C)CC[C@H](C(C)C)CC)C
TZS008	C(CCCC(=O)OC[C@@H](O)CO)CCC/C=C\C/C=C\CCCCC
WMA001	O1[C@H](CC(=O)c2c1cc(cc2O)O)c1ccc(cc1)O
WMA002	[C@@]12([C@H]([C@H]3[C@@H]([C@@]4(C(=CC3)C[C@H](CC4)O)C)CC2)CC[C@@H]1[C@H](C)CC[C@H](C(C)C)CC)C
WMA003	o1c2c(c(=O)c(c1c1ccc(cc1)O)O)c(cc(c2)O)O
WMA004	[C@@]12([C@H]([C@H]3[C@@H]([C@@]4(C(=CC3)C[C@H](CC4)O)C)CC2)CC[C@@H]1[C@H](C)/C=C/[C@H](C(C)C)CC)C
WMA005	[C@@]12([C@H]([C@H]3[C@@H]([C@@]4(C(=CC3)C[C@H](CC4)O)C)CC2)CC[C@@H]1[C@H](C)CC[C@@H](C)C(C)C)C
WMA006	C(=C\C/C=C\C/C=C\CCCCC)\C/C=C\CCCC(=O)OC
WMA007	[C@@]12([C@H]([C@H]3[C@@H]([C@@]4(C(=CC3)C[C@H](CC4)O)C)CC2)CC[C@@H]1[C@H](C)CCCC(C)C)C
WMA008	o1c2c(c(=O)c(c1c1cc(c(cc1)O)O)O)c(cc(c2)O)O
XSA001	O1[C@@]2([C@@]3([C@]4([C@@H](C3)[C@](O[C@@H]14)(C2)O)COC(=O)c1ccccc1)O)C
XSA002	O=C1C[C@@H]2[C@](CCCC2(C)C)(c2c1cc(c(c2)O)C(C)C)C
XSA003	[C@@]12([C@H]([C@H]3[C@@H]([C@@]4(C(=CC3)C[C@H](CC4)O)C)CC2)CC[C@@H]1[C@H](C)CC[C@H](C(C)C)CC)C
XSA004	[C@@]12([C@H]([C@H]3[C@@H]([C@@]4(C(=CC3)C[C@@H](CC4)O)C)CC2)CC[C@@H]1[C@H](C)CC[C@H](C(C)C)CC)C
XSA005	O1[C@H]([C@@H]([C@H]([C@@H]([C@H]1O[C@@H]1[C@@H]2O[C@@]2([C@@H]2[C@H]1C=CO[C@H]2O)CO)OC(=O)C)OC(=O)/C=C\c1ccc(cc1)OC)OC(=O)C)C
XSA006	C1C[C@H]([C@@]([C@@H]2[C@@]1([C@@H](C(=C)CC2)C[C@H](C1=CCOC1=O)S(=O)(=O)O)C)(C)CO)O
XSA007	O1[C@@]2([C@H]1[C@@H]([C@@H]1[C@H]2[C@H](OC=C1)O[C@@H]1O[C@H]([C@@H]([C@H]([C@H]1O)O)O)CO)O[C@@H]1O[C@H]([C@@H]([C@H]([C@H]1OC(=O)C)OC(=O)/C=C\c1ccccc1)OC(=O)C)C)CO
XSA008	O1[C@H]([C@@H]([C@H]([C@@H]([C@H]1O[C@H]1[C@H]2O[C@]2([C@H]2[C@@H]1C=CO[C@@H]2O)CO)OC(=O)C)OC(=O)/C=C\c1ccccc1)OC(=O)C)C
XSA009	[C@H]12[C@]([C@@H](C[C@]1(C)OC(=O)/C=C/c1ccccc1)O)(C=CO[C@@H]2O)O
ZBM001	C1(=C(C=C2C(=CC(=C[C@H]2O1)O)O)O)c1ccc(cc1)O

Compounds	Simplified Molecular-input Line-entry System formats
ZBM002	[C@@]12([C@H]([C@H]3[C@@H]([C@@]4(C(=CC3)C[C@H](CC4)O)C)CC2)CC[C@@H]1[C@H](C)CC[C@H](C(C)C)CC)C
ZBM003	O1[C@@]2(C(=C3[C@H]([C@H]4[C@@H]([C@@]5([C@@H](C(=O)C4)C[C@H](CC5)O)C)C3)CC2)C)[C@@H]([C@@H]2NC[C@H](C[C@@H]12)C)C
ZBM004	c1(c(cc(cc1OC)[C@H]1[C@H]2[C@@H](CO1)C(=O)OC2)OC)O
ZBM005	N12CC3=C(CC[C@H]4[C@H]5[C@@H]([C@@]6([C@@H]([C@@H](C5)O)C[C@@H](CC6)O)C)C[C@@H]34)[C@H]([C@@H]1CC[C@H](C2)C)C
ZBM006	O=C1c2c(cc(cc2C(=O)C(=C1C(=O)C)C)OC)O[C@@H]1O[C@@H]([C@H]([C@@H]([C@H]1O)O)O)CO
ZBM007	O1C(=O)[C@H](CCC[C@H](COC(=O)[C@H](CCC[C@H](C1)C)[C@H]1N[C@H](NC1)N)C)[C@H]1N[C@H](NC1)N

Note: Corresponding compounds names refer to Table S1. This table was retrieved from the supplementary file of our previously published paper. Detailed information is provided as follow: Li, H., Komori, A., Li, M., Chen, X., Yang, A. W. H., Sun, X., ... & Zhou, L. (2023). Multi-ligand molecular docking, simulation, free energy calculations and wavelet analysis of the synergistic effects between natural compounds baicalein and cubebin for the inhibition of the main protease of SARS-CoV-2. *Journal of Molecular Liquids*, *374*, 121253.

Table S4. Details of the normal distribution test of the docking results (kcal/mol).

Formula/Herbs	Skewness	Kurtosis	Mean	Standard deviation	Standard error of mean	95% Confidence interval of mean	
						Lower	Upper
TQG	1.23	1.33	-9.02	1.13	0.31	-9.64	-8.41
CBM	1.05	3.36	-7.72	0.93	0.23	-8.17	-7.28
CHA	-0.73	2.09	-8.38	0.58	0.18	-8.74	-8.02
DQY	-0.21	-1.04	-8.11	0.70	0.18	-8.47	-7.76
FLA	2.30	6.85	-7.42	0.66	0.11	-7.64	-7.20
HQA	-0.66	-0.75	-7.86	0.58	0.13	-8.11	-7.60
HQB	0.28	0.05	-7.65	1.31	0.27	-8.18	-7.11
JYH	-0.15	-0.16	-7.81	0.62	0.13	-8.07	-7.56
LQA	-2.04	4.44	-7.76	0.38	0.08	-7.92	-7.61
QHA	0.01	-1.19	-7.73	0.59	0.12	-7.97	-7.49
QHB	1.49	NA	-8.07	0.69	0.40	-8.85	-7.28
SCG	0.44	-0.66	-7.75	1.49	0.53	-8.78	-6.72
TZS	1.54	2.95	-7.65	0.89	0.32	-8.27	-7.03
WMA	-1.11	1.59	-8.20	0.45	0.15	-8.49	-7.91
XSA	-0.13	-1.28	-8.24	1.11	0.42	-9.07	-7.42
ZBM	1.23	1.33	-9.02	1.13	0.31	-9.64	-8.41

Note: CBM: Chuan bei mu (Fritillariae Cirrhosae Bulbus); CHA: Chai hu (Bupleuri Radix); DQY: Da qing ye (Isatidis Folium); FLA: Fu ling (Poria); HQA: Huang qin (Scutellariae Radix); HQB: Huang qi (Scutellariae Radix); JYH: Jin Yin hua (Lonicerae Japonicae Flos); LQA: Lian qiao (Forsythiae Fructus); QHA: Qing hao (Artemisiae Annuae Herba); QHB: Qian hu (Peucedani Radix); SCG: Shan ci gu (Pseudobulbus Cremastrae Seu Pleiones); TQG: Toujie Quwen Granules; TZS: Tai zi shen (Pseudostellariae Radix); WMA: Wu mei (Mume Fructus); XSA: Xuan shen (Scrophulariae Radix); ZBM: Zhe bei mu (Fritillariae Thunbergii Bulbus). It could be considered as normal distributions (absolute values of skewness < 3; absolute values of kurtosis < 8).

Table S5. Raw data of multiple ligand molecular docking results (kcal/mol).

Ligands	Binding affinity	Ligands	Binding affinity	Ligands	Binding affinity	Ligands	Binding affinity	Ligands	Binding affinity	Ligands	Binding affinity
CBM001	-6.2	CHA012	-7.2	FLA009	-7.7	HQA018	-7.6	HQB006	-7.3	JYH010	-9.1
CBM002	-8.2	CHA013	-8.3	FLA010	-9	HQA019	-7.7	HQB007	-9	JYH011	-10.1
CBM003	-8.3	CHA014	-8.7	FLA011	-7.2	HQA020	-7.5	HQB008	-7.3	JYH012	-7.5
CBM004	-9.9	CHA015	-7.9	FLA012	-7.4	HQA021	-7.7	HQB009	-8.3	JYH013	-8
CBM005	-9.7	CHA016	-7.5	FLA013	-7	HQA022	-7.6	HQB010	-7.4	JYH014	-8.2
CBM006	-9.4	CHA017	-7.8	FLA014	-7.6	HQA023	-7.6	HQB011	-7	JYH015	-7.1
CBM007	-7.7	DQY001	-8.2	FLA015	-8.2	HQA024	-7.6	HQB012	-7.7	JYH016	-6.7
CBM008	-9.6	DQY002	-8.3	HQA001	-7.5	HQA025	-7.5	HQB013	-7.3	JYH017	-8.3
CBM009	-8.6	DQY003	-9.7	HQA002	-7.5	HQA026	-8.6	HQB014	-7.8	JYH018	-6.3
CBM010	-10	DQY004	-7.4	HQA003	-7.4	HQA027	-8.3	HQB015	-7.5	JYH019	-8.7
CBM011	-10.3	DQY005	-8.1	HQA004	-7.6	HQA028	-6.5	HQB016	-8.9	JYH020	-7.5
CBM012	-10.1	DQY006	-8.7	HQA005	-7.8	HQA029	-5.1	HQB017	-7.5	JYH021	-8.5
CBM013	-9.3	DQY007	-7.8	HQA006	-7.7	HQA030	-6.8	HQB018	-8.7	JYH022	-7.7
CHA001	-5	DQY008	-8.4	HQA007	-7.6	HQA031	-8	HQB019	-8	JYH023	-7.8
CHA002	-9.4	DQY009	-8.6	HQA008	-6.9	HQA032	-7.5	HQB020	-7.8	LQA001	-7.5
CHA003	-8.6	DQY010	-8.6	HQA009	-7.7	HQA033	-5.1	JYH001	-5.2	LQA002	-8
CHA004	-7.6	FLA001	-8.7	HQA010	-7.8	HQA034	-7.4	JYH002	-4.8	LQA003	-7.2
CHA005	-7.6	FLA002	-7.9	HQA011	-7.3	HQA035	-7.4	JYH003	-7.4	LQA004	-6.9
CHA006	-7.1	FLA003	-8.3	HQA012	-7.4	HQA036	-7.5	JYH004	-7.6	LQA005	-7.7
CHA007	-7.7	FLA004	-9.3	HQA013	-7.6	HQB001	-8.4	JYH005	-6.8	LQA006	-8.8
CHA008	-7.7	FLA005	-8.2	HQA014	-7.9	HQB002	-7.3	JYH006	-5.8	LQA007	-7.7
CHA009	-8.8	FLA006	-9.2	HQA015	-7.5	HQB003	-7.7	JYH007	-9.8	LQA008	-8
CHA010	-7	FLA007	-8.5	HQA016	-7.5	HQB004	-8.6	JYH008	-9.3	LQA009	-8.8
CHA011	-7.4	FLA008	-7.5	HQA017	-7.4	HQB005	-7.6	JYH009	-7.7	LQA010	-8.4
LQA011	-7.8	QHA004	-7.6	QHA020	-7.7	QHB014	-6.9	TZS003	-10	XSA003	-8.2
LQA012	-7.8	QHA005	-7.7	QHA021	-7.5	QHB015	-7	TZS004	-8.6	XSA004	-7.8
LQA013	-8.6	QHA006	-7.5	QHA022	-7.8	QHB016	-6.9	TZS005	-7.7	XSA005	-8.6
LQA014	-6.6	QHA007	-8.5	QHB001	-7.2	QHB017	-7.1	TZS006	-8.6	XSA006	-7.6

Ligands	Binding affinity	Ligands	Binding affinity	Ligands	Binding affinity	Ligands	Binding affinity	Ligands	Binding affinity	Ligands	Binding affinity
LQA015	-6.9	QHA008	-7.6	QHB002	-7.9	QHB018	-8.5	TZS007	-8.7	XSA007	-8
LQA016	-8	QHA009	-7.5	QHB003	-7.1	QHB019	-8.3	TZS008	-5.5	XSA008	-9.2
LQA017	-7.1	QHA010	-7.7	QHB004	-7.4	QHB020	-7.8	WMA001	-7.8	XSA009	-8.3
LQA018	-7.9	QHA011	-7.5	QHB005	-8	QHB021	-8.8	WMA002	-7.2	ZBM001	-7.3
LQA019	-7.5	QHA012	-7.8	QHB006	-8	QHB022	-7.8	WMA003	-7.5	ZBM002	-7.5
LQA020	-7.9	QHA013	-7.4	QHB007	-8.1	QHB023	-8.4	WMA004	-8.5	ZBM003	-9.9
LQA021	-7.7	QHA014	-7.9	QHB008	-8.4	QHB024	-7.8	WMA005	-8.6	ZBM004	-6.6
LQA022	-9.1	QHA015	-7.4	QHB009	-6.8	SCG001	-8.7	WMA006	-5.6	ZBM005	-9.5
LQA023	-7.8	QHA016	-7.6	QHB010	-7.1	SCG002	-8.4	WMA007	-8.2	ZBM006	-8.2
QHA001	-7.8	QHA017	-9	QHB011	-7.8	SCG003	-7.1	WMA008	-7.8	ZBM007	-8.7
QHA002	-7.6	QHA018	-7.5	QHB012	-8.6	TZS001	-5.5	XSA001	-8.2		
QHA003	-8.3	QHA019	-7.9	QHB013	-7.8	TZS002	-7.4	XSA002	-7.9		

Note: Corresponding compounds names refer to Table S1.

Table S6. Raw data of Luciferase assays.

Baicalein / Arbidol	0			Mean	Stand deviation	95% Confidence interval of mean	
	Test 1	Test 2	Test 3			Lower	Upper
0	4.51	4.43	4.47	4.47	0.03	4.43	4.51
10 IC_{50}	4.23	4.24	4.23	4.23	0.00	4.23	4.24
20 IC_{50}	4.23	4.21	4.2	4.21	0.01	4.20	4.23
40 IC_{50}	4.17	4.14	4.08	4.13	0.04	4.09	4.17
50 IC_{50}	4.12	4.06	4.02	4.07	0.04	4.02	4.11
60 IC_{50}	4.08	4.05	4.01	4.05	0.03	4.01	4.08
80 IC_{50}	3.95	3.95	3.94	3.95	0.00	3.94	3.95
100 IC_{50}	3.87	3.84	3.78	3.83	0.04	3.79	3.87
**Baicalein / Arbidol**	**10 IC_{50}**			**Mean**	**Stand deviation**	**95% Confidence interval of mean**	
	**Test 1**	**Test 2**	**Test 3**			**Lower**	**Upper**
0	4.41	4.48	4.4	4.43	0.04	4.39	4.47
10 IC_{50}	4.24	4.14	4.06	4.15	0.07	4.06	4.23
20 IC_{50}	4.19	4.17	4.03	4.13	0.07	4.05	4.21
40 IC_{50}	4.22	4.08	4.01	4.10	0.09	4.00	4.20
50 IC_{50}	4.06	4.04	4	4.03	0.02	4.01	4.06
60 IC_{50}	4.02	4.02	4.01	4.02	0.00	4.01	4.02
80 IC_{50}	3.91	3.92	3.89	3.91	0.01	3.89	3.92
100 IC_{50}	3.72	3.7	3.8	3.74	0.04	3.69	3.79
**Baicalein / Arbidol**	**20 IC_{50}**			**Mean**	**Stand deviation**	**95% Confidence interval of mean**	
	**Test 1**	**Test 2**	**Test 3**			**Lower**	**Upper**
0	4.21	4.25	4.23	4.23	0.02	4.21	4.25
10 IC_{50}	4.12	4.03	4.01	4.05	0.05	4.00	4.11
20 IC_{50}	4.12	4.02	3.91	4.02	0.09	3.92	4.11
40 IC_{50}	3.95	3.99	3.95	3.96	0.02	3.94	3.98
50 IC_{50}	3.89	3.89	3.94	3.91	0.02	3.88	3.93
60 IC_{50}	3.74	3.89	3.92	3.85	0.08	3.76	3.94
80 IC_{50}	3.79	3.83	3.83	3.82	0.02	3.80	3.84
100 IC_{50}	3.61	3.66	3.76	3.68	0.06	3.61	3.75
**Baicalein / Arbidol**	**40 IC_{50}**			**Mean**	**Stand deviation**	**95% Confidence interval of mean**	
	**Test 1**	**Test 2**	**Test 3**			**Lower**	**Upper**
0	4.13	4.11	3.97	4.07	0.07	3.99	4.15
10 IC_{50}	4.03	3.95	3.9	3.96	0.05	3.90	4.02
20 IC_{50}	3.96	3.89	3.84	3.90	0.05	3.84	3.95
40 IC_{50}	3.89	3.81	3.79	3.83	0.04	3.78	3.88
50 IC_{50}	3.8	3.81	3.81	3.81	0.00	3.80	3.81
60 IC_{50}	3.75	3.73	3.71	3.73	0.02	3.71	3.75

80 IC_{50}	3.68	3.71	3.73	3.71	0.02	3.68	3.73
100 IC_{50}	3.56	3.67	3.59	3.61	0.05	3.55	3.66
**aicalein** / **Arbidol**	**60 IC_{50}**			**Mean**	**Stand deviation**	**95% Confidence interval of mean**	
	**Test 1**	**Test 2**	**Test 3**			**Lower**	**Upper**
0	4	4	3.92	3.97	0.04	3.93	4.02
10 IC_{50}	3.9	3.88	3.92	3.90	0.02	3.88	3.92
20 IC_{50}	3.91	3.84	3.8	3.85	0.05	3.80	3.90
40 IC_{50}	3.75	3.79	3.76	3.77	0.02	3.75	3.79
50 IC_{50}	3.8	3.78	3.65	3.74	0.07	3.67	3.82
60 IC_{50}	3.72	3.71	3.65	3.69	0.03	3.66	3.73
80 IC_{50}	3.75	3.59	3.64	3.66	0.07	3.58	3.74
100 IC_{50}	3.51	3.59	3.64	3.58	0.05	3.52	3.64
**aicalein** / **Arbidol**	**80 IC_{50}**			**Mean**	**Stand deviation**	**95% Confidence interval of mean**	
	**Test 1**	**Test 2**	**Test 3**			**Lower**	**Upper**
0	3.94	3.91	3.94	3.93	0.01	3.91	3.95
10 IC_{50}	3.88	3.83	3.85	3.85	0.02	3.83	3.88
20 IC_{50}	3.83	3.84	3.75	3.81	0.04	3.76	3.85
40 IC_{50}	3.82	3.74	3.64	3.73	0.07	3.65	3.82
50 IC_{50}	3.8	3.66	3.62	3.69	0.08	3.61	3.78
60 IC_{50}	3.66	3.6	3.59	3.62	0.03	3.58	3.65
80 IC_{50}	3.55	3.58	3.57	3.57	0.01	3.55	3.58
100 IC_{50}	3.55	3.54	3.45	3.51	0.04	3.46	3.56
**aicalein** / **Arbidol**	**100 IC_{50}**			**Mean**	**Stand deviation**	**95% Confidence interval of mean**	
	**Test 1**	**Test 2**	**Test 3**			**Lower**	**Upper**
0	3.81	3.92	3.92	3.88	0.05	3.82	3.94
10 IC_{50}	3.83	3.73	3.8	3.79	0.04	3.74	3.83
20 IC_{50}	3.73	3.69	3.66	3.69	0.03	3.66	3.73
40 IC_{50}	3.61	3.65	3.6	3.62	0.02	3.60	3.64
50 IC_{50}	3.6	3.59	3.5	3.56	0.04	3.51	3.61
60 IC_{50}	3.54	3.56	3.44	3.51	0.05	3.45	3.57
80 IC_{50}	3.44	3.43	3.42	3.43	0.01	3.42	3.44
100 IC_{50}	3.27	3.39	3.39	3.35	0.06	3.29	3.41

Data S1. Data of metabolomics profiling of Toujie Quwen Granules (positive and negetive modes).

Provided in a separate Excel file.

Data S2. The grid box parameters for blind docking.

center_x = -21.5235
center_y = 12.5976
center_z = 47.12
size_x = 59.6911188602
size_y = 66.9736678314
size_z = 81.6870348406
